# Bioactivities of Steroids and Sesquiterpenes from the Branches and Leaves of *Aglaia lawii*

**DOI:** 10.3390/molecules29010039

**Published:** 2023-12-20

**Authors:** Jingjing Dong, Hanfei Liu, Huan Wang, Huayong Lou, Weidong Pan, Jinyu Li

**Affiliations:** 1 State Key Laboratory of Functions and Applications of Medicinal Plants, Guizhou Medical University, Guiyang 550014, China; dongjingjing1994@outlook.com (J.D.); liuhanfei000@163.com (H.L.); whhh5579@163.com (H.W.); 2 Natural Products Research Center of Guizhou Province, Guiyang 550014, China; 3 School of Pharmaceutical Sciences, Guizhou University, Guiyang 550025, China

**Keywords:** *Aglaia lawii*, steroids, sesquiterpenes, cytotoxicity, antibacterial

## Abstract

Five undescribed steroids and one sesquiterpene, named Aglaians A–F, along with sixteen known analogs, have been isolated from the branches and leaves of *Aglaia lawii*. Its structure was elucidated by IR, HRESIMS, 1D and 2D NMR, quantum-chemical calculations, electronic circular dichroism (ECD) calculations, and single-crystal X-ray diffraction analysis. The cytotoxic and antibacterial activities of six human tumor cell lines were evaluated (MDA-MB-231, MCF-7, Ln-cap, A549, HeLa, and HepG-2), and four strains of bacteria (*Bacterium subtilis*, *Phytophthora cinnamomic*, *Acrogenic bacterium*, and *Ralstonia solanacearum*). The bioassay results indicated that compounds **3** and **5** exhibited moderate antitumor activity with IC_50_ values ranging from 16.72 to 36.14 μM. Furthermore, compounds **3**–**5** possess antibacterial activities against four bacteria with MIC values of 25–100 μM.

## 1. Introduction

**Figure 1:**
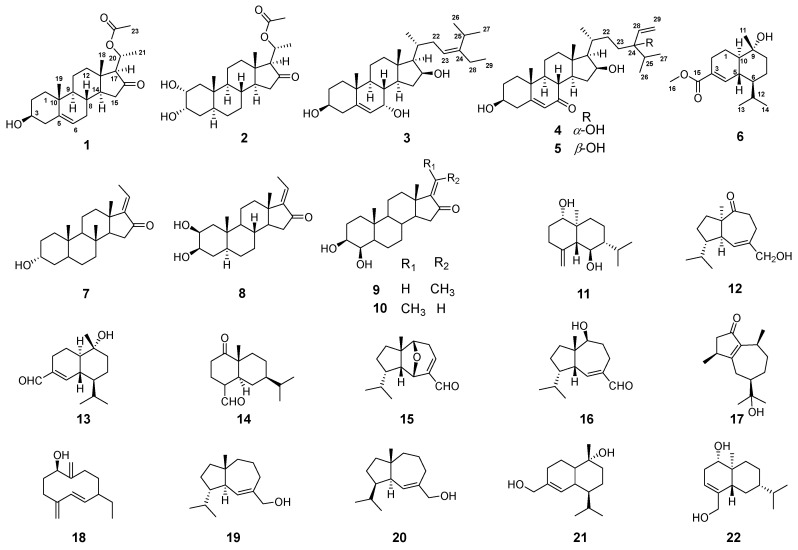
Compounds (**1**–**22**) isolated from the leaves and branches of *A. lawii*.

*Aglaia lawii* (Meliaceae), a perennial tree, is mainly distributed in the tropical and subtropical areas of Asia (such as Guangzhou, Guizhou, and Yunnan Provinces, China). The leaves of *A. lawii* are a traditional folk medicine used as an antibacterial and anti-tumor [1,2,3,4]. Previously, our research group has been devoted to the medicinal plants of the family Meliaceae, from which some limonoids and triterpenoids with novel structures have been found. These compounds exhibit diverse biological activities, such as anti-tumor, anti-inflammatory, and anti-bacterial [5,6]. Currently, existing evidence indicates that *A. lawii* predominantly comprises sesquiterpenes and triterpenes, conferring anti-inflammatory properties. However, fewer studies have reported on *A. lawii*, and its chemical constituents remain to be systematically isolated and identified, with their biological activities not extensively explored [1,2,3,4]. As part of our ongoing work, we extracted structurally novel and biologically active ethanol extract from the branches and leaves of *A. lawii* and discovered six previous undescribed steroids and sesquiterpene (**1**–**6**) (Figure 1), along with sixteen known analogs (**7**–**22**) (Figure 1). Their structures were characterized by comprehensive spectroscopic data analysis, ECD calculations, and X-ray crystallography. Furthermore, their preliminary cytotoxic and antibacterial activities were evaluated through in vitro assay. In the present paper, the isolation, structure elucidation, and biological evaluation of these compounds were presented.

## 2. Results

**Figure 2:**
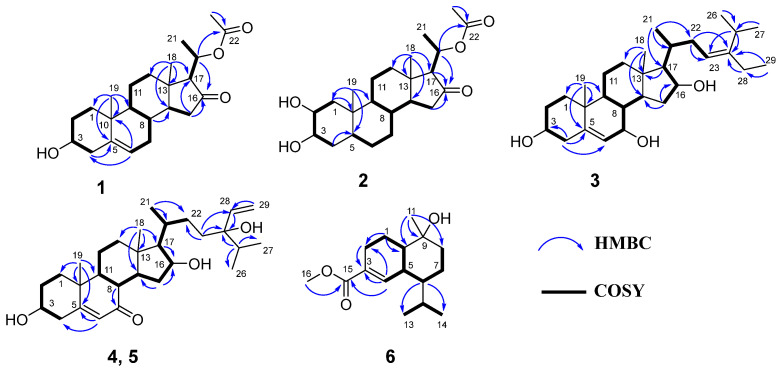
^1^H-^1^H COSY and key HMBC correlations of compounds **1**–**6**.

Aglaian A (**1**) was obtained as a colorless acicular crystal. The high-resolution electrospray ionization mass spectrometry (HRESIMS) data showed that the molecular formula was C_23_H_34_O_4_, and the positive ion peak was *m*/*z* 397.2344 [M + Na]^+^ (calcd for C_23_H_34_O_4_Na 397.2349 Figure S7), indicating six degrees of unsaturation. The infrared radiation (IR Figure S8) absorption bands revealed the existence of hydroxy (3502 cm^−1^) and carbonyl (1737 cm^−1^) groups, respectively. The ^1^H and ^13^C NMR data (Table 1 and Table 2) of **1** displayed the characteristic resonances of four methyls (*δ*_H_ 2.01 (s), 1.42 (d, *J* = 6.3 Hz), 1.02 (s), and 0.79 (s), each 3H; *δ*_C_ 21.6, 20.4, 19.5, and 13.5), seven sp^3^ methylenes, a olefinic methine (*δ*_H_ 5.34, *δ*_C_ 120.9), six sp^3^ methines, including two oxymethines (*δ*_H_ 3.52, 5.06, *δ*_C_ 71.7, 69.4), five quaternary carbons, including one ester carbonyl (*δ*_C_ 170.1) and a keto group (*δ*_C_ 215.5), which are characteristic signals for C21 steroid. Further careful analysis of the nuclear magnetic resonance (NMR) data indicated the structure of **1** was similar to that of 5,6-dehydrotoosendanesterol A [7]. The obvious differences were the presence of an acetyl group (*δ*_C_ 21.6, 170.1) in **1** instead of a methyl group in 5,6-dehydrotoosendanesterol A. This deduction was further verified by the heteronuclear multiple boan correlation (HMBC) (Figure 2) from H-20 (*δ*_H_ 5.06) to *δ*_C_ 170.1, indicating that an acetyl group was attached to C-20.

**Figure 3:**
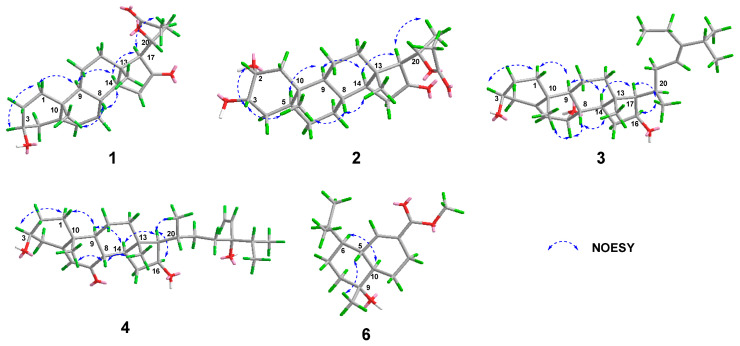
Key NOESY correlations (blue arrow) of compounds **1**–**4**, **6**.

**Figure 4:**
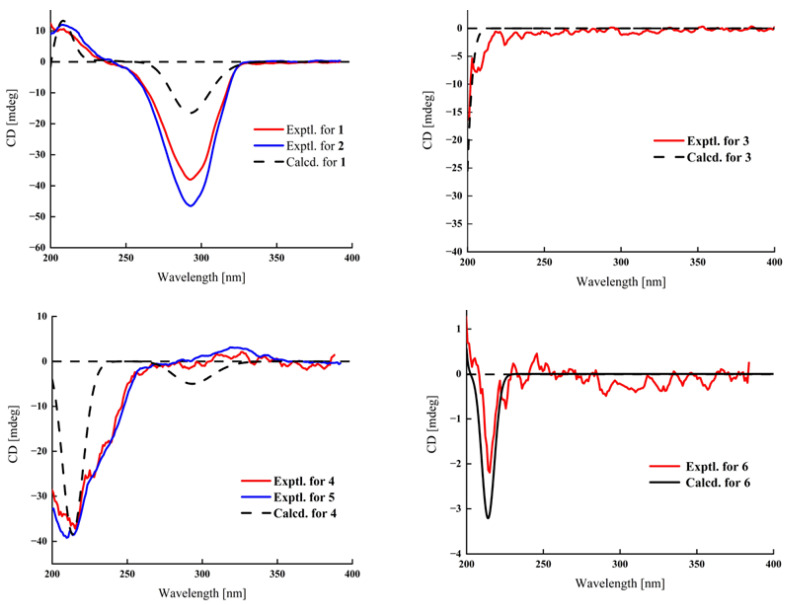
Experimental and calculated ECD spectra of **1**–**6**.

**Figure 5:**
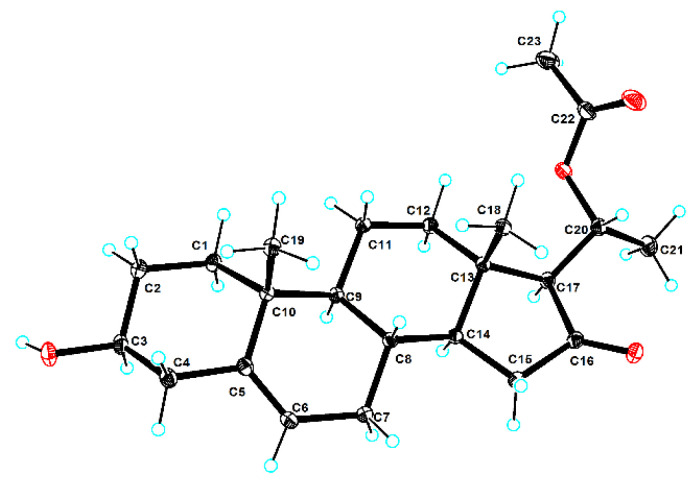
The X-ray ORTEP drawing of **1.** The red circle stands for oxygen atom and the blue circle stands for hydrogen atom.

The relative configuration of **1** was established according to the nuclear overhauser effect spectroscopy (NOESY) (Figure 3). The NOESY correlations of H-1*α*/H-3, H-9, H-9/H-14, H-17/H-14, and H-21 indicated these protons were cofacial and randomly assigned to be *α*-orientations. In turn, the correlations of H-8/H-19 and H-18 demonstrated that H-8, H-18, and H-19 were *β*-oriented. Finally, the ECD calculations (Figure 4) combined with the single-crystal X-ray diffraction experiment (flack parameter: 0.05 (8)) with Cu Kα radiation experiment (Figure 5) further established the absolute configuration of **1** as 3*S*, 8*S*, 9*S*, 10*R*, 13*S*, 14*S*, 17*S*, 20*R*. Thus, compound **1** was defined as shown in Figure 1.

Aglaian B (**2**) is a white amorphous powder, and its molecular formula is established as C_23_H_36_O_5_ by *m*/*z* 415.2452 [M + Na]^+^ (calcd for C_23_H_36_O_5_Na 415.2455), indicating that there are six hydrogen deficiency indexes. The ^1^H and ^13^C NMR data (Table 1 and Table 2) of **2** displayed the characteristic resonances of four methyls (*δ*_H_ 2.01 (s), 1.41 (d, *J* = 6.6 Hz), 0.81 (s), and 0.75 (s), each 3H; *δ*_C_ 21.6, 20.4, 12.7, and 12.5), an acetyl group (*δ*_H_ 2.01 (s, 3H), *δ*_C_ 21.6, 170.2) and a keto group (*δ*_C_ 215.7). Comprehensive analysis of its 2D NMR data (Figure 2) indicated the structure of **2** was similar to that of 2α,3α,16β-trihydroxy-20-acetoxy-20(R)-pregnane [8]. The only difference was the presence of a carbonyl group (*δ*_C_ 215.7) at C-16 in **2** instead of a hydroxyl in 2α,3α,16β-trihydroxy-20-acetoxy-20(R)-pregnane. The HMBC correlation between H-15 (*δ*_H_ 2.25, 1.87), H-17 (*δ*_H_ 2.12), H-20 (*δ*_H_ 5.04), and C-16 (*δ*_C_ 215.7) (Figure 2) further validates this inference, indicating that there is a carbon base on C-16. Analysis of the NOESY correlations of H-2/H-3, H-19; H-8/H-19, and H-18 suggested that H-2, H-3, H-19, H-8, and H-18 were *β*-oriented. The remaining stereocenters were the same as those of **1**. Furthermore, the similarity of the experimental ECD spectra (Figure 4) of **2** and **1** established the absolute configuration of **2** as 2*R*, 3*S*, 5*S*, 8*R*, 9*S*, 10*S*, 13*S*, 14*S*, 17*S*, 20*R*. Therefore, we describe the structure of **2**, as shown in Figure 1.

Aglaian C (**3**) was obtained as a white amorphous powder. The positive HRESIMS ion at *m*/*z* 467.3491 [M + Na]^+^ (calcd for C_29_H_48_O_3_Na 467.3495) determined the molecular formula of C_29_H_48_O_3_, implying six degrees of unsaturation. The IR absorption bands revealed the presence of a hydroxy group (3533 cm^−1^) and double bonds (1654 cm^−1^), respectively. The ^1^H and ^13^C NMR data (Table 1 and Table 2) of **3** displayed the characteristic resonances of six methyls (*δ*_H_ 1.59 (m), 1.02 (d, *J* = 6.6 Hz), 1.00 (s), 0.98 (d, *J* = 6.9 Hz), 0.98 (d, *J* = 6.9 Hz), and 0.89 (s), each 3H; *δ*_C_ 21.2, 21.1, 18.6, 18.4, 12.9, and 12.8), two olefinic methines (*δ*_H_ 5.15 (m), 5.60 (d, *J* = 5.4 Hz), *δ*_C_ 146.5, 146.1, 123.9, and 117.0), respectively. After careful analysis of its HMBC and ^1^H-^1^H correlated spectroscopy (COSY) (Figure 2), the structure of **3** was similar to that of 3β,7α,16β-trihydroxy-stigmast-5-ene [9]. The obvious difference between them was observation of an additional double bond (*δ*_H_ 5.15 (H-23), *δ*_C_ 117.0 (C-23), 146.5 (C-24)) in **3**, in place of those for the sp3 methylene (*δ*_C_ 117.0 (C-23)) and methine (*δ*_C_ 146.5 (C-24)) groups in 3β,7α,16β-trihydroxy-stigmast-5-ene. This deduction was further certified by the HMBC correlations (Figure 2) from H-22 (*δ*_H_ 1.63), H-25 (*δ*_H_ 2.84), H-28 (*δ*_H_ 1.85), and H-29 (*δ*_H_ 1.59) to C-24, and H-22 (*δ*_H_ 1.63), H-25 (*δ*_H_ 2.84), and H-28 (*δ*_H_ 1.85) to C-23. The Z-configuration for H-23 and H-24 was determined based on their NOESY correlations (Figure 3) of H-23 and H-28. The relative configuration of **3** as verified by the NOESY correlations of H-1*α*/H-3, H-9; H-14/H-9, 16, and H-17; H-17/H-21 indicated H-3, H-9, H-14, H-16, H-17, and CH_3_-21 were cofacial and arbitrarily assigned as *α*-orientations, in contrast to the *β*-oriented of H-8/H-7, H-19, and H-18 correlations. Finally, the absolute configuration of **3** (3*S*, 7*S*, 8*S*, 9*S*, 10*R*, 13*S*, 14*S*, 16*S*, 17*R*, 20*R*) was defined by the well-matched experimental and calculated ECD spectra (Figure 4). Thus, the structure of **3** was determined, as shown in Figure 1.

Aglaian D (**4**) was obtained as a colorless oil and possessed the molecular formula of C_29_H_46_O_4_, with seven degrees of unsaturation, as assigned by HRESIMS ions at *m*/*z* 481.3287 [M + Na]^+^ (calcd for C_29_H_46_O_4_Na 481.3288). The IR absorption bands revealed the existence of hydroxy (3446 cm^−1^) and double bonds (1660 cm^−1^), respectively. The ^1^H and ^13^C NMR data (Table 1 and Table 2) of **4** displayed the characteristic resonances of five methyls (*δ*_H_ 1.20 (s), 0.96 (d, *J* = 6.6 Hz), 0.87 (d, *J* = 5.9 Hz), 0.87 (d, *J* = 5.9 Hz), and 0.85 (s), each 3H; *δ*_C_ 18.6, 17.8, 17.4, 16.7, and 13.1), a keto group (*δ*_C_ 202.2), respectively. Further careful analysis of the HMBC and ^1^H-^1^H COSY spectra (Figure 2) indicated the structure of **4** was similar to that of (24*R*)-5, 28-stigmastadiene-3*β*, 24-diol-7-one [10], and the main difference the hydrogen on C-16 in (24*R*)-5, 28-stigmastadiene-3*β*, 24-diol-7-one was replaced by a hydroxyl group in **4**. The chemical shift of C-16 (*δ*_C_ 72.7) in **4** was obviously downfield. Along with the HMBC correlations (Figure 2) from H-15 (*δ*_H_ 3.06, 1.33), H-17 (*δ*_H_ 0.96), and H-20 (*δ*_H_ 1.76) to C-16, indicating that a hydroxyl group was attached to C-16. The NOESY cross-peaks of H-1*α*/H-3, H-9; H-14/H-9, H-17, H-17/H-16, and CH_3_-21 indicated that their relative configurations were *α*-oriented. However, the lack of NOESY correlations to 24-OH resulted in the unknown stereochemistry of C-24. The stereochemistry of **4** at C-24 was established to be *R*, through the chemical shift difference between H-27 (0.873) and H-26 (0.863) was 0.010 ppm, whereas the chemical shift difference between H-27 (0.873) and H-21 (0.963) was 0.110 ppm, which was in accordance with those of (24*R*)-5,28-stigmastadiene-3*β*,24-diol-7-one [10,11]. The absolute configuration of **4** was defined as 3*S*, 8*S*, 9*S*, 10*R*, 13*S*, 14*S*, 16*S*, 17*R*, 20*R*, 24*R* via the comparison of the experimental ECD spectrum with calculated (Figure 4). Thus, the structure of **4** was elucidated, as shown in Figure 1.

Aglaian E (**5**) was obtained as a colorless oil and possessed the same molecular formula as **4** based on its HRESIMS ion peak at *m*/*z* 481.3289 [M + Na]^+^ (C_29_H_46_O_4_Na, calcd. 481.3288). The NMR and IR spectra of **5** showed very resemblances to those of **4**, except for the differences in ^13^C NMR data (Table 1 and Table 2) between C-23, C-24, C-25, and C-28, which suggested that **5** and **4** were epimers. The difference is that C24 of **5** was *S*. The chemical shift difference between H-27 (0.888) and H-26 (0.877) of **5** was 0.011 ppm, whereas the chemical shift difference between H-27 (0.888) and H-21 (0.966) of compound **5** was 0.078 ppm, which was in accordance with those of (24*S*)-5, 28-stigmastadiene-3*β*, 24-diol-7-one [10,11]. Consequently, the absolute configuration of **5** was defined as 3*R*, 8*S*, 9*S*, 10*R*, 13*S*, 14*S*, 16*S*, 17*R*, 20*R*, 24*S* by comparing its experimental ECD spectrum with that of **4** (Figure 4). Therefore, ignoring C-24, compound **5** possessed the same absolute configuration as **4**. Thus, the structure of **5** was characterized as shown in Figure 1.

Aglaian F (**6**) was obtained as a colorless oil and presented a molecular formula of C_16_H_26_O_3_ according to the HRESIMS ion at *m*/*z* 289.1768 [M + Na]^+^ (calcd for C_16_H_26_O_3_Na 289.1774), with four degrees of unsaturation. Its IR absorption suggested the presence of hydroxy (3533 cm^−1^) and carbonyl (1726 cm^−1^) functionalities. The NMR data (Table 1 and Table 2) of **6** displayed the characteristic resonances of three methyls (*δ*_H_ 3.73 (s), 0.95 (d, *J* = 6.9 Hz), and 0.81 (d, *J* = 6.9 Hz), each 3H; *δ*_C_ 51.8, 21.6, and 15.3) and methyl formate (*δ*_C_ 168.3, 51.8). Further careful analysis of the HMBC and ^1^H-^1^H COSY (Figure 2) indicated the structure of **6** was similar to that of dysodensiol D [12]. The obvious differences were the presence of a methyl formate group (*δ*_C_ 51.8, 168.3) at C-3 in **6**, instead of a carboxyl in dysodensiol D. This was verified by the ^1^H-^1^H COSY (Figure 2) cross-peaks of H-1 (*δ*_H_ 2.12)/H-2 (*δ*_H_ 2.49, 2.22), H-4 (*δ*_H_ 7.06)/H-5 (*δ*_H_ 1.88), combined with HMBCs from H-4 (*δ*_H_ 7.06) and H-16 (*δ*_H_ 3.73) to C-15 (*δ*_C_168.3). On the basis of the NOESY spectrum (Figure 3), the relative configuration of compound **6** was established. The NOESY cross-peaks of H-6/H-10 and H-5/H-11 suggested that H-6 and H-10 were *α*-oriented, while the H-5 and H-11 were of *β*-orientation. In addition, its calculated ECD curves were matched well with the experimental ECD curves (Figure 4), which further corroborated the absolute configuration of **6** (5*R*, 6*S*, 9*R*, 10*R*). Thus, the structure of **6** was determined, as shown in Figure 1.

Furthermore, Sixteen known components from *A. lawii* were identified to be (*E*)-aglawone (**7**) [13], 2*β*,3*β*-oihydroxy-5*α*- pregn-l7(20)-(*Z*)-en-16-one (**8**) [14], (*Z*)-toonasterone C (**9**) [15], (*E*)-toonasterone C (**10**) [15], ent-4(15)-eudesmene-1β,6α-diol **(11)** [16], (+)-aphanamol I (**12**) [17], 10a-hydroxycadin-4-en-15-al (**13**) [18], 1-oxo-5*α*,7*α*H-eudesma-3-en-15-al (**14**) [19], amouanglienoid A (**15**) [2], aphanamol II (**16**) [20], pancherio-ne (**17**) [21], 1*β*-hydroxy-4(15),5E,10(14)-germacratriene (**18**) [21], isodauc-6-ene-10*β*,14-diol (**19**) [22], 4-epi-isodauc-6-ene-10*β*,14-diol (**20**) [22], 15-hydroxy-*α*-cadinol (**21**) [23], and commiphorane I (**22**) [24], by comparing their spectroscopic data with those reported.

In accordance with the ethnomedical indication of this plant in Chinese folk medicine, all the isolates were tested in vitro for their cytotoxicity against six human cancer cell lines and four antibacterial activities. The novel compounds **3** and **5** displayed moderate cytotoxicity, with IC_50_ values ranging from 16.72 to 36.14 μM (Table 3). Compounds **3**, **4**, and **5** have weak antibacterial activities against four bacteria with MIC values of 25–100 μM (Table 4).

## 3. Discussion

An in-depth study on the petroleum ether (PE) fraction of the EtOH extraction of the branches and leaves of *A. lawii* led to the isolation of six previous undescribed steroids and sesquiterpenes (**1**–**6**), along with sixteen known analogs (**7**–**22**). The new structures were elucidated by IR, HRESIMS, 1D and 2D NMR, ECD calculations, and single-crystal X-ray diffraction analysis. All compounds were evaluated for their cytotoxicity against six human cancer cell lines (MDA-MB-231, MCF-7, Ln-cap, A549, HeLa, and HepG-2) and antibacterial activity against four strains (*B. subtilis, P. cinnamomi, A. bacterium*, and *R. solanacearum*). The results showed that compounds **3** and **5** exhibited moderate cell inhibitory activity with IC_50_ values ranging from 16.72 to 36.14 μM. Meanwhile, compounds **3**–**5** have weak antibacterial activities with MIC values of 25–100 μM. These results enrich the structure and bioactivity diversity of *A. lawii* from the Meliaceae family.

## 4. Materials and Methods

### 4.1. General Experimental Procedures

Nuclear magnetic resonance (NMR) spectroscopy was performed on a Bruker Avance NEO (600 MHz for ^1^H and 150 MHz for ^13^C) spectrometer (Bruker, Karlsruhe, Germany). The chemical shifts (*δ*) are given in ppm and coupling constants (*J*) are given in hertz (Hz). HRESIMS data were obtained on an Agilent 6210 ESI/TOF mass spectrometer (Agilent, Santa Clara, CA, USA). UV spectra were recorded on an Agilent UV-Vis Cary 60 spectrometer (Agilent Corporation, Santa Clara, CA, USA). IR spectra were recorded on a Nicolet iS5 FT-IR spectrometer. ECD spectra were obtained by an Applied Photophysics Chirascan circular dichroism spectrometer. Optical rotations were recorded on a Horiba SEPA-300 polarimeter. Semi-preparative HPLC was performed on an Agilent 1100 (Agilent Technologies, Santa Clara, CA, USA). The columns were a Waters C18 (5 µm, 10 mm × 250 mm) column (Made in Ireland) and a Hungpu phenyl (5 µm, 10 mm × 250 mm) column (Guangzhou Hungpu Technology Co., Ltd., Guangzhou, China). Silica gel (60–80, 200–300, and 300–400 mesh, Qingdao Marine Chemical Co. Ltd., Qingdao, China) and Sephadex LH-20 (25–100 µm, Amersham Biosciences, Uppsala, Sweden) were used for column chromatography (CC). TLC was conducted on pre-coated silica gel GF254 plates (Qingdao Haiyang Chemical Co., Ltd., Qingdao, China), and spots were detected by spraying with 5% H_2_SO_4_ in EtOH followed by heating.

### 4.2. Plant Material

The stem bark of *A. lawii* was collected in March 2022 from Wangmo Guizhou Province of China, People’s Republic of China, and identified by Prof. Chao-yi Deng, Qianxinan Karst Regional Development Institute of Guizhou. A voucher specimen (no. 20220301) was deposited at the Natural Products Research Center of Guizhou Province.

### 4.3. Extraction and Isolation

The air-dried branches and leaves (15.0 kg) of *A. lawii* were powder and extracted with 95% EtOH. The filtrate was evaporated under reduced pressure to give crude extract (1 kg), which was suspended in H_2_O (2 L). Then, the mixture was extracted with petroleum ether (PE) (5 L × 6) and EtOAc (5 L × 5) to afford the PE (530.0 g). Eight fractions (A-H) were obtained by elution of PE fractions with MeOH-H_2_O gradient (*v*/*v*, 40–100%) phase C18 vacuum column. Fraction E (40.0 g) was separated by petroleum ethyl acetate (*v*/*v*, 10:1/1:1) to obtain 10 fractions (E1–E10). Fr.E6 (512.0 mg) precipitated and crystallized to obtain **1** (70.0 mg). E2 subfraction (788 mg) was separated on a Sephadex LH-20 with CHCl_3_/CH_3_OH (1:1, *v*/*v*) and then separated by preparative HPLC (MeOH-H_2_O, *v*/*v*, 75:25) to obtain compounds **12** (28 mg, *t*_R_ = 56 min), and **13** (9 mg, *t*_R_ = 52 min). Fr. E3 (1.2 g) was further purified by repeated Sephadex LH-20 column and HPLC (MeCN-H_2_O, *v/v*, 65:35) to obtain compounds **22** (23 mg, *t*_R_ = 26 min), **8** (5 mg, *t*_R_ = 41 min), **9** (8 mg, *t*_R_ = 32 min), and **10** (8 mg, t_R_ = 32 min). Thirteen fractions (D1–D13) were obtained by silica gel column separation with petroleum ethyl acetate (*v*/*v*, 10:1/1:1). Fr.D4 (235 mg) was separated on a Sephadex LH-20 column (CHCl_3_/CH_3_OH 1:1, *v*/*v*), and then separated by preparative HPLC (MeCN-H_2_O, *v*/*v*, 40:60), giving **11** (20 mg, *t*_R_ = 22 min). Compounds **19** (16 mg, *t*_R_ = 38 min) and **20** (20 mg, *t*_R_ = 44 min) were obtained by preparative HPLC (MeCN-H_2_O, *v*/*v*, 70:30). Fr.D6 (166 mg) was by preparative HPLC (MeCN-H_2_O, *v*/*v*, 75:25) to provide **17** (9 mg, *t*_R_ = 11 min). Fr. D8 (261 mg) was by preparative HPLC (MeCN-H_2_O, *v*/*v*, 70:20) to yield **21** (16 mg, *t*_R_ = 28 min). Fraction F (32.0 g) was separated by a silica gel column with petroleum–EtOAc (*v*/*v*, 20:1–1:1) to generate 13 fractions (F1–F13). Fr.F2 (421 mg) was by preparative HPLC (MeCN-H_2_O, *v/v*, 50:50) to yield **14** (42 mg, *t*_R_ = 26 min). Fr.F4 (248 mg) was by preparative HPLC (MeCN-H_2_O, *v*/*v*, 65:35) to obtain **15** (5 mg, *t*_R_ = 36 min). Fr.F7 (328 mg) was by preparative HPLC (MeCN-H_2_O, *v*/*v*, 75:25) to provide **6** (4 mg, *t*_R_ = 26 min) and **16** (6 mg, *t*_R_ = 32 min). Fraction G (50.0 g) was separated by a silica gel column with petroleum–EtOAc (*v*/*v*, 40:1–1:1) to generate 11 fractions (G1–G11). Fr.G3 (823 mg) was given **3** (54 mg, *t*_R_ = 36 min) by preparative HPLC (MeCN-H_2_O, *v*/*v*, 60:40). Fr.G4 (126 mg) produced **18** (12 mg, *t*_R_ = 46 min) by preparative HPLC (MeCN-H_2_O, *v*/*v*, 70:30). Fr.G7 (372 mg) was supplied **7** (12 mg, t_R_ = 16 min) by preparative HPLC (MeCN-H_2_O, *v*/*v*, 85:15). Fr.G10 (211 mg) was given **2** (13 mg, *t*_R_ = 28 min) by preparative HPLC (MeCN-H_2_O, *v*/*v*, 75:25). G11 (188 mg) was separated by a Sephadex LH-20 column (CHCl_3_/CH_3_OH 1:1, *v*/*v*), and then separated by preparative HPLC (MeCN-H_2_O, *v*/*v*, 70:30) to obtain compounds **4** (8 mg, *t*_R_ = 18 min) and **5** (6 mg, *t*_R_ = 21 min). (See the Supporting Information for compounds separation flowchart).

### 4.4. Spectroscopic Data of the New Compounds

Aglaian A (**1**): colorless acicular crystal; mp 211.1–213.2 °C;${\left[\alpha \right]}_{D}^{25}\;$−30.3 (c 0.33 CH_3_OH); UV (MeOH) *λ*_max_ (log *ε*) 200 (3.26) nm; IR *v*_max_ (Microscope) 3502, 2944, 1737,1246 cm^−1^; HR-ESI-MS *m*/*z* 397.2344 [M + Na]^+^ (C_23_H_34_O_4_Na, calcd. 397.2349); ^1^H and ^13^C NMR data see Table 1 and Table 2.

Aglaian B (**2**): white amorphous powder; ${\left[\alpha \right]}_{D}^{25}\;$−18.9 (c 0.53 CH_3_OH); UV (MeOH) *λ*_max_ (log *ε*) 200 (1.89) nm; IR *v*_max_ (Microscope) 3441, 1743 cm^−1^; HR-ESI-MS *m*/*z* 415.2452 [M + Na]^+^ (C_23_H_36_O_5_Na, calcd. 415.2455); ^1^H and ^13^C NMR data see Table 1 and Table 2.

Aglaian C (**3**): white amorphous powder; ${\left[\alpha \right]}_{D}^{25}\;$−3.2 (c 0.85 CH_3_OH); UV (MeOH) *λ*_max_ (log *ε*) 200 (2.06) nm; IR *v*_max_ (Microscope) 3533, 2958, 1655 cm^−1^; HR-ESI-MS *m*/*z* 467.3492 [M + Na]^+^ (C_29_H_48_O_3_Na, calcd. 467.3496); ^1^H and ^13^C NMR data see Table 1 and Table 2.

Aglaian D (**4**): colorless oil; ${\left[\alpha \right]}_{D}^{25}$ −23.1 (c 0.42 CH_3_OH); UV (MeOH) *λ*_max_ (log *ε*) 238 (2.16) nm; IR *v*_max_ (Microscope) 3446, 2948, 1660 cm^−1^; HR-ESI-MS *m*/*z* 481.3287 [M + Na] (C_29_H_46_O_4_Na, calcd. 481.3288); ^1^H and ^13^C NMR data see Table 1 and Table 2.

Aglaian E (**5**): colorless oil; ${\left[\alpha \right]}_{D}^{25}\;$−23.0 (c 0.61 CH_3_OH); UV (MeOH) *λ*_max_ (log *ε*) 237 (2.53) nm; IR *v*_max_ (Microscope) 3418, 2947, 1652 cm^−1^; HR-ESI-MS *m*/*z* 481.3289 [M + Na] (C_29_H_46_O_4_Na, calcd. 481.3288); ^1^H and ^13^C NMR data see Table 1 and Table 2.

Aglaian F (**6**): colorless oil; ${\left[\alpha \right]}_{D}^{25}\;$−28.2 (c 0.45 CH_3_OH); UV (MeOH) *λ*_max_ (log *ε*) 221 (2.47) nm; IR *v*_max_ (Microscope) 3433, 2957, 1715 cm^−1^; HR-ESI-MS *m*/*z* 289.1768 [M + Na]^+^ (C_16_H_26_O_3_Na, calcd. 289.1774); ^1^H and ^13^C NMR data see Table 1 and Table 2.

### 4.5. Cytotoxicity Assay

The cytotoxic activity of the compounds against human tumor cell lines (231, MCF-*7*, Ln-Cap, A549, HeLa, and HepG-2) was analyzed through the MTT method [25]. Briefly, cells were treated with different concentrations of compounds and incubated for 48 h after being seeded in 96-well plates for 24 h at 37 °C with 5% CO_2_. Untreated cells were incubated in a medium, and the medium served as control and blank, respectively. Meanwhile, doxorubicin was used as a positive control. The supernatant was removed, and the MTT solution (5 mg/mL) was prepared in a medium, which was added to each well until the formazan crystal was fully dissolved. The absorbance of each well was detected by an enzyme-labeled instrument at a wavelength of 490 nm [25].

### 4.6. Antibacterial Assay

The compounds were assessed against four Gram-positive bacteria: *B. subtilis*, *P. cinnamomi*, *A. bacterium*, and *R. solanacearum*. The minimum inhibitory concentrations (MICs) were determined in 96-well culture plates by a serial dilution of each compound with concentrations ranging from 12.5–100 µm and repeated three times according to the standard microdilution method [26]. Each concentration of samples was added to three bacterial culture wells to ensure the repeatability of the experiment. After incubation at 37 °C for 24 h, the lowest concentration of antibiotics without visible bacterial growth were the MICs. Ofloxacin was used as a positive control.

### 4.7. X-Ray Diffraction Analysis

The colorless acicular crystal of Aglaian A (**1**) was obtained from acetone at a temperature of 4 °C. The X-ray diffraction data were selected and performed on an XtaLAB AFC12 (RINC): Kappa single diffractometer. The crystal was kept at 99.98(11) K, 99.99(10) K, 99.99(10) K, and 100.00(10) K, respectively, during data collection. The structure was solved with the SHELX structure solution program using Intrinsic Phasing, and all non-hydrogen atoms were refined with the SHELX refinement package using least squares minimization. The crystallographic data of the reported structure were deposited in the Cambridge Crystallographic Data Center (CCDC) with a deposition number of 2303496.

Crystal Data for C_23_H_34_O_4_ (*M* = 374.50 g/mol): orthorhombic, space group *P*212121 (no. 19), a = 5.70310 (10) Å, b = 10.8236 (2) Å, c = 33.1051 (4) Å, V = 2043.51 (6) Å^3^, Z = 4, T = 99.96 (13) K, *μ*(Cu Kα) = 0.647 mm^−1^, Dcalc = 1.217 g/cm^3^, 19,741 reflections measured (5.338 ≤ 2Θ ≤ 148.786), 4094 unique (Rint = 0.0454, Rsigma = 0.0289), which were used in all calculations. The final R_1_ was 0.0328 (*I* > 2σ(*I*)), and wR_2_ was 0.0850 (all data). The flack parameter was 0.05 (8).

### 4.8. ECD Calculations

The conformational search was carried out by the SYBYL-X 2.0 software at the MMFF94s force field. The relative configurations were reoptimized by using the time-dependent density functional theory (TDDFT) at the B3LYP/6-31G(d) level in the gas phase via the Gaussian 16 program, and the frequency was calculated at the same level of theory [27]. The optimized stable conformers without imaginary frequencies were performed at the B3LYP-SCRF (PCM)/6-31+G(d) level in the PCM MeOH model using the TDDFT method [27]. The absolute configurations of compounds **1**–**6** were analyzed with SpecDis software 1.71 by comparing the experimental ECD spectra.

## Figures and Tables

**Table 1:** ^1^H NMR (600 MHz) data of compounds **1**–**6** in CDCl_3_ (*δ* in ppm and *J* in Hz).

No.	1	2	3	4	5	6
1	*α* 1.86, m *β* 1.09, m	*α* 1.74, m *β* 1.26, m	*α* 1.85, m *β* 1.11, m	*α* 1.95, m *β* 1.26, m	*α* 1.95, m *β* 1.20, m	2.12, m
2	*α* 1.95, m *β* 1.60, m	3.76, m	*α* 1.50, m *β* 1.85, m	*α* 1.61, m *β* 1.92, m	*α* 1.68, m *β* 1.92, m	*α* 2.49, m *β* 2.22, m
3	3.52, m	3.96, m	3.58, m	3.66, m	3.69, m	
4	*α* 2.30, (d, 13.2) *β* 2.27, (d, 7.8)	*α* 1.51, m *β* 1.49, m	*α* 2.33, (d, 12.0) *β* 2.28, (d, 13.2)	*α* 2.40, (d, 11.4) *β* 2.51, (d, 13.8)	*α* 2.39, (d, 13.8) *β* 2.50, (d, 14.1)	7.06, (d, 3.3)
5		1.53, m				1.88, m
6	5.34, m	*α* 1.31, m *β* 1.17, m	5.60, (d, 5.4)	5.68, s	5.68, s	1.17, m
7	*α* 1.84, m *β* 1.50, m	*α* 1.63, m *β* 1.01, m	3.84, m			*α* 1.66, m *β* 1.21, m
8	1.64, m	1.58, m	1.59, m	2.32, m	2.32, m	*α* 1.83, m *β* 1.44, m
9	1.12, m	0.96, m	1.21, m	1.49, m	1.56, m	
10						1.27, m
11	*α* 1.53, m *β* 1.64, m	*α* 1.36, m *β* 1.70, m	1.54, m	1.56, m	1.61, m	1.12, (d, 0.9)
12	*α* 2.24, m *β* 1.53, m	*α* 1.99, m *β* 1.44, m	*α* 2.14, m *β* 2.00, m	*α* 1.08, m *β* 2.01, m	*α* 1.08, m *β* 2.01, m	2.18, m
13						0.81, (d, 6.9)
14	1.47, m	1.46, m	1.32, m	1.15, m	1.21, m	0.95, (d, 6.9)
15	*α* 1.88, (d, 13.5) *β* 2.04, (d, 8.7)	*α* 2.25, (d, 18.6) *β* 1.87, (d, 22.5)	*α* 2.36, m *β* 1.21, m	*α* 3.06, m *β* 1.33, m	*α* 3.08, m *β* 1.30, m	
16			4.42, m	4.41, m	4.41, m	3.73, s
17	2.09, (d, 9.6)	2.12, (d, 9.6)	1.08, m	0.96, (d, 6.6)	0.96, (d, 6.6)	
18	0.79, s	0.75, s	0.89, s	0.85, s	0.88, s	
19	1.02, s	0.81, s	1.00, s	1.20, s	1.23, s	
20	5.06, m	5.04, m	1.85, m	1.76, m	1.71, m	
21	1.42, (d, 6.3)	1.41, (d, 6.6)	1.02, (d, 6.6)	0.963, (d, 6.6)	0.966, (d, 6.6)	
22			1.63, m	a 1.67, m b 1.04, m	a 1.68, m b 1.05, m	
23	2.01, s	2.01, s	5.15, m	1.87, m	1.82, m	
25			2.84, m	a 1.76, m b 1.44, m	a 1.82, m b 1.48, m	
26			0.98, (d, 6.9)	0.863, (d, 5.9)	0.877, (d, 3.6)	
27			0.98, (d, 6.9)	0.873, (d, 5.9)	0.888, (d, 3.9)	
28			1.85, m	5.85, m	5.73, m	
29			1.59, m	a 5.21, (d, 1.5) b 5.14, (d, 1.5)	a 5.21, (d, 1.5) b 5.17, (d, 1.5)	

**Table 2:** ^13^C NMR (150 MHz) data of compounds **1**–**6** in CDCl_3_.

No.	1	2	3	4	5	6
1	37.1	40.7	37.1	36.4	36.4	22.2
2	31.9	69.1	31.4	31.3	31.6	25.4
3	71.7	69.3	71.5	70.6	70.6	130.9
4	42.3	33.7	42.1	42.0	42.0	140.3
5	141.1	38.2	146.1	165.5	165.5	40.7
6	120.9	27.5	123.9	126.1	126.1	46.0
7	31.6	32.0	65.4	202.2	202.2	22.2
8	30.9	343	37.5	45.1	45.1	42.8
9	49.9	540	42.4	50.0	50.0	72.3
10	36.7	37.1	37.2	38.5	38.5	49.2
11	20.7	20.6	20.5	20.9	20.9	20.7
12	39.1	39.1	39.4	38.9	38.8	26.2
13	42.7	43.0	42.1	43.0	43.0	15.3
14	50.4	50.2	47.5	48.0	48.1	21.6
15	39.0	39.1	36.4	38.4	38.3	168.3
16	215.5	215.7	72.7	72.7	72.8	51.8
17	67.0	67.2	61.1	60.2	60.5	
18	13.5	12.7	12.9	13.1	13.2	
19	19.5	12.5	18.4	17.4	17.5	
20	69.4	69.4	30.2	36.4	37.6	
21	20.4	20.4	18.6	18.6	19.0	
22	170.1	170.2	36.1	29.4	29.6	
23	21.6	21.6	117.0	30.5	31.3	
24			146.5	78.0	79.1	
25			28.8	35.1	34.1	
26			21.1	16.7	16.8	
27			21.2	17.8	17.8	
28			28.0	146.2	142.2	
29			12.8	113.2	113.9	

**Table 3:** Antitumor activity of compounds **1**–**6** (IC_50,_ μM).

Compounds	MDA-MB-231	MCF-7	Ln-cap	A549	HeLa	HepG-2
**1**	>50	>50	>50	>50	>50	>50
**2**	>50	>50	>50	>50	>50	>50
**3**	36.14 ± 1.32	22.10 ± 0.16	17.85 ± 0.42	21.08 ± 1.14	22.56 ± 1.32	23.22 ± 1.33
**4**	>50	>50	>50	>50	>50	>50
**5**	24.31 ± 1.13	24.99 ± 1.31	16.72 ± 1.40	19.67 ± 1.68	23.54 ± 1.33	29.59 ± 1.05
**6**	>50	>50	>50	>50	>50	>50
doxorubicin	1.31 ± 0.48	1.51 ± 0.32	0.57 ± 0.04	0.57 ± 0.08	1.29 ± 0.11	1.75 ± 0.11

**Table 4:** Antibacterial activities of compounds **1**–**6** (MIC, μM).

Compounds	*B. subtilis*	*P. cinnamomi*	*A. bacterium*	*R. solanacearum*
**1**	≥100	≥100	≥100	≥100
**2**	≥100	≥100	≥100	≥100
**3**	≥50	≥100	≥100	≥100
**4**	≥100	≥100	≥100	≥100
**5**	≥25	≥50	≥100	≥25
**6**	≥100	≥100	≥100	≥100
ofloxacin	0.41	1.69	1.69	0.41

## Data Availability

Data are contained within the article and supplementary materials.

## Supplementary Materials

The following supporting information can be downloaded at: https://www.mdpi.com/article/10.3390/molecules29010039/s1. Figures S1–S61: The 1D and 2D NMR spectra, UV, CD, IR spectra, as well as HRESIMS data of compounds **1**–**6**. Tables S1 and S2: ECD calculation details of of **1**–**6**. Table S3: Crystal data and structure refinement for compound **1**.

## References

[1] Li J.-F., Ji K.-L., Sun P., Cai Q., Zheng X.-L., Xiao Y.-D., Cao D.-H., Xiao C.-F., Zhang Z.-Y., Li X.-N. (2021). Structurally diverse steroids with nitric oxide inhibitory activities from *Aglaia lawii* leaves. Phytochemistry.

[2] Xia M.-J., Zhang M., Li S.-W., Cai Z.-F., Zhao T.-S., Liu A.-H., Luo J., Zhang H.-Y., Li J., Guo Y.-W. (2022). Anti-inflammatory and PTP1B inhibitory sesquiterpenoids from the twigs and leaves of *Aglaia lawii*. Fitoterapia.

[3] Li J.-F., Xu Y.-K. (2018). Constituents from the leaves and twigs of *Amoora ouangliensis* and their anti-inflammatory activities. Nat. Prod. Res. Dev..

[4] Yang S.-M., Wu D.-G., Liu X.-K. (2010). Anticancer activity of diterpenoids from *Amoora ouangliensis* and *Amoora stellato-squamosa*. Z. Naturforsch. C. J. Biosci..

[5] Zhang D.-Y., Lou H.-Y., Chen C., Liu H.-F., Deng C.-Y., Li J.-Y., Pan W.-D. (2022). Cipacinerasins A–K, structurally diverse limonoids from Cipadessa baccifera. Phytochemistry.

[6] Shao L.-L., Liu H.-F., Lou H.-Y., Ma F.-W., Chen C., Li J.-Y., Pan W.-D. (2022). Dammarane and apotirucallane triterpenoids from the stem bark of *Melia toosendan* and their antibacterial activities. Tetrahedron.

[7] Yuan C.-M., Tang G.-H., Wang X.-Y., Zhang Y., Guo F., Liao J.-H., Zou T., Zuo G.-Y., Hua H.-M., He H.-P. (2013). Two new compounds from *Khaya senegalensis*. J. Asian. Nat. Prod. Res..

[8] Pan X., Matsumoto M., Nishimoto Y., Ogihara E., Zhang J., Ukiya M., Tokuda H., Koike K., Akihisa M., Akihisa T. (2014). Cytotoxic and nitric oxide production-inhibitory activities of limonoids and other compounds from the leaves and bark of *Melia azedarach*. Chen. Biodivers..

[9] Lou X.-D., Wu S.-H., Ma Y.-B., Wu D.-G. (2001). The chemical constituents of *Amoora yunnanensis*. J. Integr. Plant Biol..

[10] Li G.-L., Guo W.-J., Wang G.-B., Wang R.-R., Hou Y.-X., Liu K., Liu Y., Wang W. (2017). Sterols from the green alga *Ulva australis*. Mar. Drugs.

[11] Chen Z., Liu J., Fu Z., Ye C., Zhang R., Song Y., Zhang Y., Li H., Ying H., Liu H. (2014). 24 (S)-Saringosterol from edible marine seaweed *Sargassum fusiforme* is a novel selective LXRβ agonist. J. Agr. Food Chem..

[12] Xie B.-J., Yang S.-P., Yue J.-M. (2008). Terpenoids from *Dysoxylum densiflorum*. Phytochemistry.

[13] Qiu S.-X., Hung N.-V., Gu J.-Q., Lobkovsky E., Khanh T.C., Soejarto D.D., Clardy J., Pezzuto J.M., Dong Y., Tri M.V. (2001). A pregnane steroid from *Aglaia lawii* and structure confirmation of cabraleadiol monoacetate by X-ray crystallography. Phytochemistry.

[14] Inada A., Murata H., Inatomi Y., Nakanishi T., Darnaedi D. (1997). Pregnanes and triterpenoid hydroperoxides from the leaves of *Aglaia grandis*. Phytochemistry.

[15] Wang J.-R., Shen Q., Fang L., Peng S.-Y., Yang Y.-M., Li J., Liu H.-L., Guo Y.-W. (2011). Structural and stereochemical studies of five new pregnane steroids from the stem bark of *Toona ciliata* var. pubescens. Steroids.

[16] Xie Y.-T., Xiong S.-H., Bian Y., Wang Y., Guan R.-Q., Suo X.-Y., Du M.-R., Liu Y.-P., Fu Y.-H. (2022). Chemical constituents from *Artocarpus incisus* and their inhibitory effects on proliferation of synoviocytes in vitro. Chin. J. Chin. Mater Med..

[17] Das J., Jha D., Policegoudra R., Mazumder A.H., Das M., Chattopadhyay P., Singh L. (2012). Isolation and characterization of antidermatophytic bioactive molecules from *Piper longum* L. leaves. Chin. J. Chin. Mater Med..

[18] Iijima T., Yaoita Y., Kikuchi M. (2003). Five New Sesquiterpenoids and a New Diterpenoid from *Erigeron annuus* (L.) P ERS., *Erigeron philadelphicus* L. and *Erigeron sumatrensis* R ETZ. Chem. Pharm. Bull..

[19] Li J., Wang F.-Q., Ding N., Zhao M., Wang J.-L., Zhang S.-J. (2018). Chemical constituents from *Syneilesis aconitifolia*. Chin. Tradit. Herbal. Drugs.

[20] Larock R., Harrison L., Hsu M. (1984). Heteroannulation via intramolecular (.pi.-allyl) palladium displacement. J. Org. Chem..

[21] Raharivelomanana P., Bianchini J.P., Faure R., Cambon A., Azzaro M. (1996). Two guaiane and eudesmane-type sesquiterpenoids from *Neocallitropsis pancheri*. Phytochemistry.

[22] Liu H.-B., Zhang C.-R., Dong S.-H., Yang S.-P., Sun Q., Geng M.-Y., Yue J.-M. (2012). Sesquiterpenes from *Dysoxylum oliganthum* and *Dysoxylum excelsum*. J. Asian Nat. Prod. Res..

[23] Kuo Y.-H., Chen C.-H., Chien S.-C., Lin Y.-L. (2002). Five new cadinane-type sesquiterpenes from the heartwood of *Chamaecyparis obtusa* var. formosana. J. Nat. Prod..

[24] Zhu S.-S., Qin D.-P., Wang S.-X., Yang C., Li G.-P., Cheng Y.-X. (2019). Commipholactam A, a cytotoxic sesquiterpenoidal lactam from *Resina Commiphora*. Fitoterapia.

[25] Kumar P., Nagarajan A., Uchil P.-D. (2018). Analysis of cell viability by the MTT assay. Cold. Spring. Harb. Protoc..

[26] Andrews J.M. (2001). Determination of minimum inhibitory concentrations. J. Antimicrob. Chemoth..

[27] Yang L.-J., Peng X.-Y., Zhang Y.-H., Liu Z.-Q., Li X., Gu Y.-C., Shao C.-L., Han Z., Wang C.-Y. (2020). Antimicrobial and antioxidant polyketides from a deep-sea-derived fungus Aspergillus versicolor SH0105. Mar. Drugs..

