# Supplementary material for: Bioactivities of Steroids and Sesquiterpenes from the Branches and Leaves of Aglaia lawii
Source: Molecules. 2023 Dec 20;29(1):39. doi: 10.3390/molecules29010039 (PMC10779827; doi:10.3390/molecules29010039)
Supplement: Supplementary file 1 [file molecules-29-00039-s001.zip › molecules-2730426-supplementary.pdf]

Bioactivities of steroids and sesquiterpenes from the branches and leaves of *Aglaia lawii*

Jing-Jing Dong[3], Han-Fei Liu[1, 2], Huan Wang[1], Hua-Yong Lou[1, 2, *], Wei-Dong Pan[3, *], Jin-Yu Li[1, 2, *]

[1] State Key Laboratory of Functions and Applications of Medicinal Plants, Guizhou Medical University, Guiyang 550014, PR China

[2] Natural Products Research Center of Guizhou Province, Guiyang 550014, PR China

[3] School of Pharmaceutical Sciences, Guizhou University, Guiyang 550025, PR China; 2840427380@qq.com

[*] Correspondence: lijinyu20080@126.com (J.-Y. Li), wdpan@163.com (W.-D. Pan), loouhy@126.com (H.-Y. Lou).

Tables of Contents

Table S1 ECD analysis parameters of **1–6**. 4
Table S2 Important thermodynamic parameters for 1–6 at B3LYP/6-31G(d) level of theory..........4
Table S3 Crystal data and structure refinement for compound 1....................5
Figure S1. ^{1}H NMR spectrum of compound **1** in $CDCl_3$ (600 MHz)....................6
Figure S2. ^{13}C NMR spectrum of compound **1** in $CDCl_3$ (150 MHz) 6
Figure S3. HSQC spectrum of compound **1** in $CDCl_3$....................7
Figure S4. HMBC spectrum of compound **1** in $CDCl_3$....................7
Figure S5. ^{1}H-^{1}H COSY spectrum of compound **1** in $CDCl_3$ 8
Figure S6. NOESY spectrum of compound **1** in $CDCl_3$ 8
Figure S7. HRESIMS spectrum of compound **1**....................9
Figure S8. IR spectrum of compound **1** 9
Figure S9. UV spectrum of compound **1** 10
Figure S10. CD spectrum of compound **1** 10
Figure S11. ^{1}H NMR spectrum of compound **2** in $CDCl_3$ (600 MHz)....................11
Figure S12. ^{13}C NMR spectrum of compound **2** in $CDCl_3$ (150 MHz) 11
Figure S13. HSQC spectrum of compound **2** in $CDCl_3$....................12
Figure S14. HMBC spectrum of compound **2** in $CDCl_3$....................12
Figure S15. ^{1}H-^{1}H COSY spectrum of compound **2** in $CDCl_3$ 13
Figure S16. NOESY spectrum of compound **2** in $CDCl_3$ 13
Figure S17. HRESIMS spectrum of compound **2**....................14
Figure S18. IR spectrum of compound **2**....................14
Figure S19. UV spectrum of compound **2** 15
Figure S20. CD spectrum of compound **2** 15
Figure S21. ^{1}H NMR spectrum of compound **3** in $CDCl_3$ (600 MHz)....................16
Figure S22. ^{13}C NMR spectrum of compound **3** in $CDCl_3$ (150 MHz) 16
Figure S23. HSQC spectrum of compound **3** in $CDCl_3$....................17
Figure S24. HMBC spectrum of compound **3** in $CDCl_3$....................17
Figure S25. ^{1}H-^{1}H COSY spectrum of compound **3** in $CDCl_3$ 18
Figure S26. NOESY spectrum of compound **3** in $CDCl_3$ 18
Figure S27. HRESIMS spectrum of compound **3**....................19
Figure S28. IR spectrum of compound **3**....................19
Figure S29. UV spectrum of compound **3** 20
Figure S30. CD spectrum of compound **3** 20
Figure S31. ^{1}H NMR spectrum of compound **4** in $CDCl_3$ (600 MHz)....................21
Figure S32. ^{13}C NMR spectrum of compound **4** in $CDCl_3$ (150 MHz) 21
Figure S33. HSQC spectrum of compound **4** in $CDCl_3$....................22
Figure S34. HMBC spectrum of compound **4** in $CDCl_3$....................22
Figure S35. ^{1}H-^{1}H COSY spectrum of compound **4** in $CDCl_3$ 23
Figure S36. NOESY spectrum of compound **4** in $CDCl_3$ 23
Figure S37. HRESIMS spectrum of compound **4**....................24
Figure S38. IR spectrum of compound **4**....................24

Figure S39. UV spectrum of compound **4** 25
Figure S40. CD spectrum of compound **4** 25
Figure S41. ^{1}H NMR spectrum of compound **5** in $CDCl_3$ (600 MHz)....26
Figure S42. ^{13}C NMR spectrum of compound **5** in $CDCl_3$ (150 MHz) 26
Figure S43. HSQC spectrum of compound **5** in $CDCl_3$....27
Figure S44. HMBC spectrum of compound **5** in $CDCl_3$....27
Figure S45. ^{1}H-^{1}H COSY spectrum of compound **5** in $CDCl_3$ 28
Figure S46. NOESY spectrum of compound **5** in $CDCl_3$ 28
Figure S47. HRESIMS spectrum of compound **5**....29
Figure S48. IR spectrum of compound **5** 29
Figure S49. UV spectrum of compound **5** 30
Figure S50. CD spectrum of compound **5** 30
Figure S51. ^{1}H NMR spectrum of compound **6** in $CDCl_3$ (600 MHz)....31
Figure S52. ^{13}C NMR spectrum of compound **6** in $CDCl_3$ (150 MHz) 31
Figure S53. HSQC spectrum of compound **6** in $CDCl_3$....32
Figure S54. HMBC spectrum of compound **6** in $CDCl_3$....32
Figure S55. ^{1}H-^{1}H COSY spectrum of compound **6** in $CDCl_3$ 33
Figure S56. NOESY spectrum of compound **6** in $CDCl_3$ 33
Figure S57. IR spectrum of compound **6**....34
Figure S58. HRESIMS spectrum of compound **6**....34
Figure S59. UV spectrum of compound **6** 35
Figure S60. CD spectrum of compound **6** 35
Figure S61. Compounds separation flowchart....36

ECD calculation details of 1–6

Table S1 ECD analysis parameters of **1–6**.

No.	half-bandwidth (eV)	UV correction (nm)
**1**	0.16	0
**2**	0.16	0
**3**	0.16	0
**4**	0.16	0
**5**	0.16	0
**6**	0.16	0

Table S2 Important thermodynamic parameters for **1–6** at B3LYP/6-31G(d) level of theory.

NO.	E[a] (Hartree)	C[b] (Hartree)	G[c] (kcal/mol)
**1**	-1197.831564	0.484682	-751347.141924
**2**	-1273.563122	0.510408	-798853.308562
**3**	-1359.620929	0.666449	-852757.525745
**4**	-1433.643746	0.647647	-898703.503488
**5**	-1433.631309	0.648259	-898695.319807
**6**	-851.037260	0.352475	-533813.209435

a Electronic energy; b Thermal correction to Gibbs free energy obtained; c Gibbs free energy (E + C).

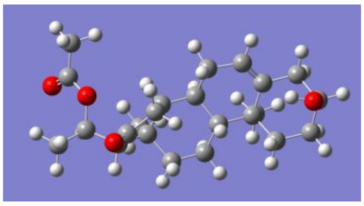

1

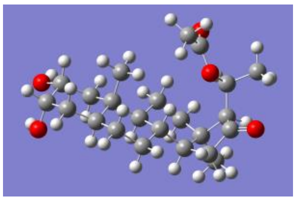

2

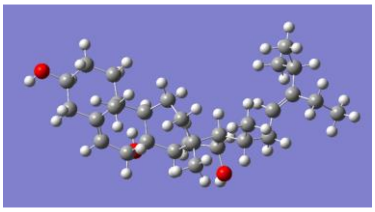

3

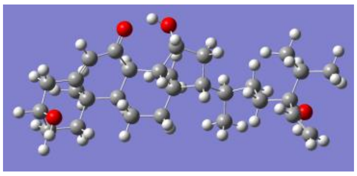

4

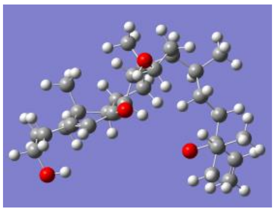

5

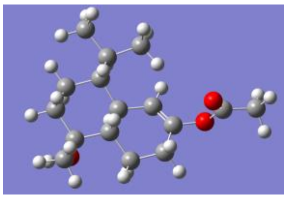

6

Table S3 Crystal data and structure refinement for compound **1**

Identification code	**1**
Empirical formula	$C_{23}H_{34}O_4$
Formula weight	374.50
Temperature/K	99.96 (13)
Crystal system	orthorhombic
Space group	$P2_12_12_1$
a/Å	5.70310(10)
b/Å	10.8236(2)
c/Å	33.1051(4)
α/°	90
β/°	90
γ/°	90
Volume/Å^3	2043.51(6)
Z	4
ρ_{calc}g/cm^3	1.217
μ/mm^{-1}	0.647
F(000)	816.0
Crystal size/mm^3	0.6 × 0.06 × 0.04
Radiation	Cu Kα (λ = 1.54184)
2Θ range for data collection/	5.338 to 148.786
Index ranges	$-6 \leq h \leq 6$, $-12 \leq k \leq 13$, $-40 \leq l \leq 41$
Reflections collected	19741
Independent reflections	4094 [R_{int} = 0.0454, R_{sigma} = 0.0289]
Data/restraints/parameters	4094/0/249
Goodness-of-fit on F^2	1.044
Final R indexes [I>=2σ (I)]	R_1 = 0.0328, wR_2 = 0.0838
Final R indexes [all data]	R_1 = 0.0347, wR_2 = 0.0850
Largest diff. peak/hole / e Å^{-3}	0.23/-0.23
Flack parameter	0.05(8)

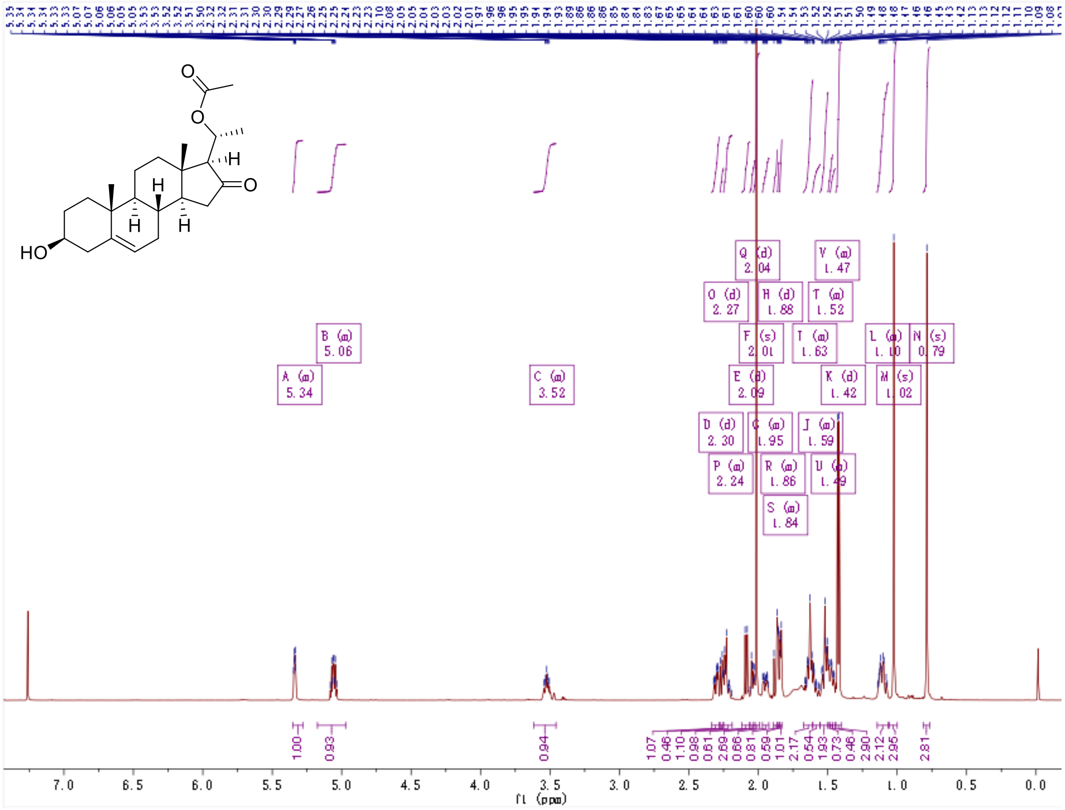

Figure S1. ^{1}H NMR spectrum of compound **1** in $CDCl_3$ (600 MHz)

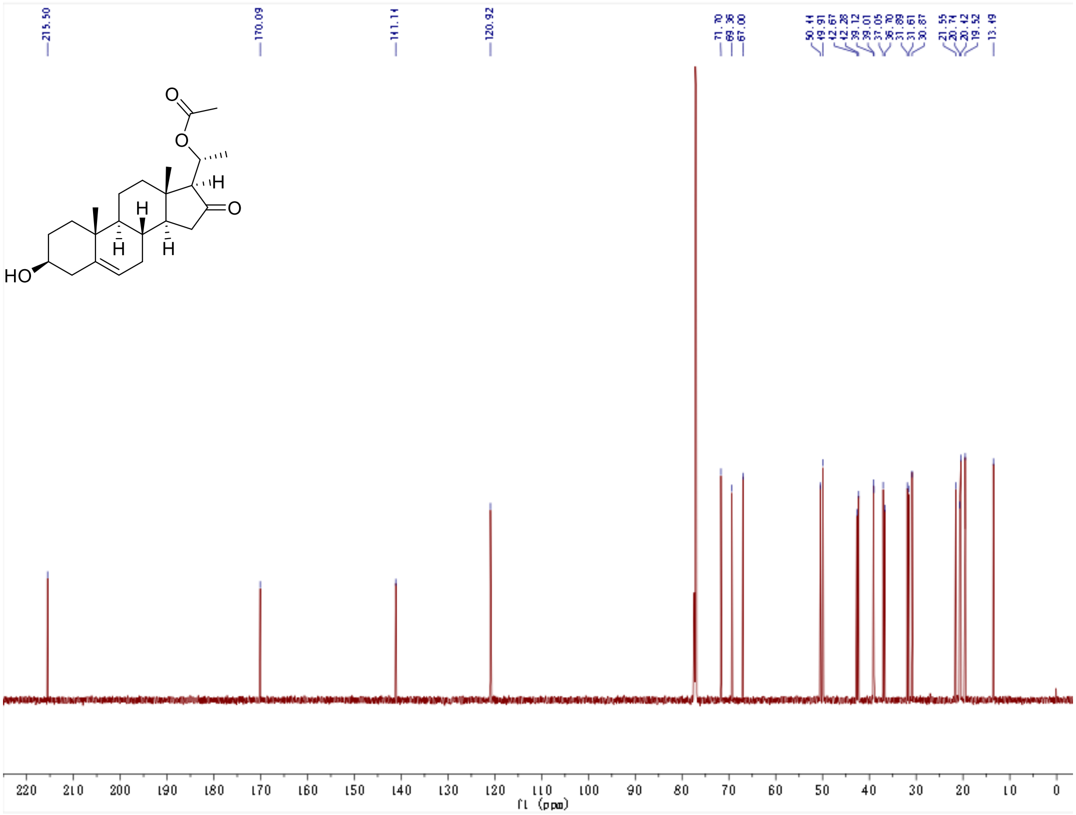

Figure S2. ^{13}C NMR spectrum of compound **1** in $CDCl_3$ (150 MHz)

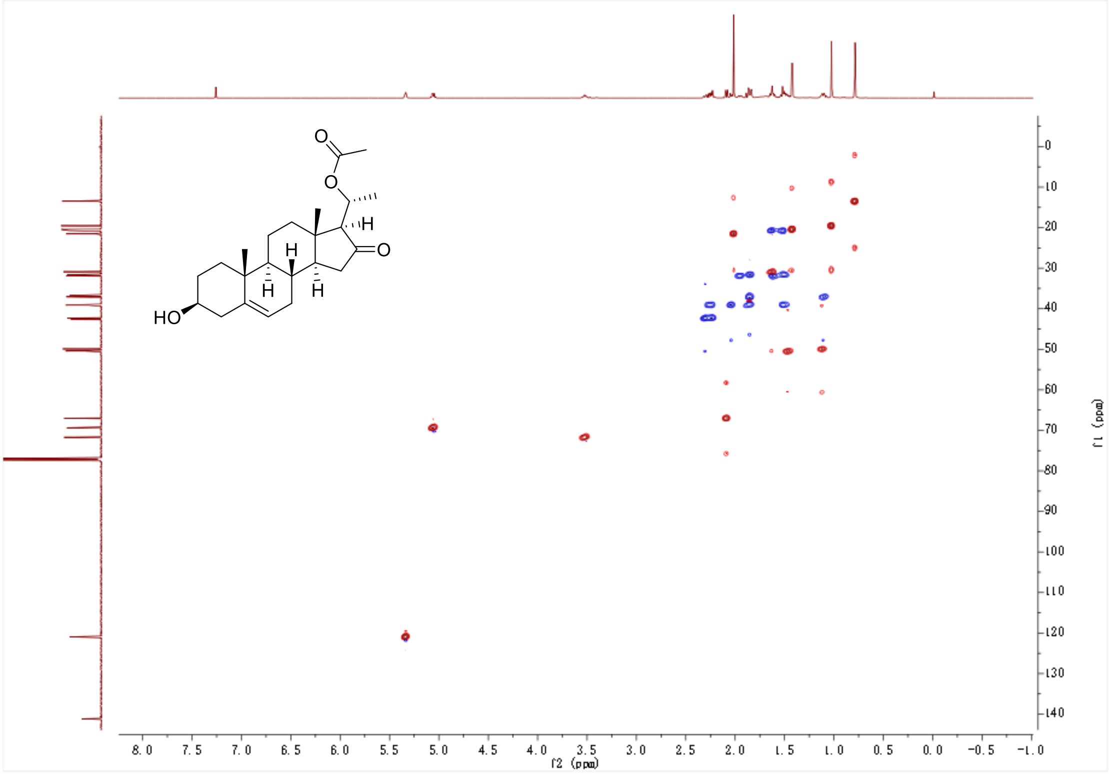

Figure S3. HSQC spectrum of compound **1** in $CDCl_3$

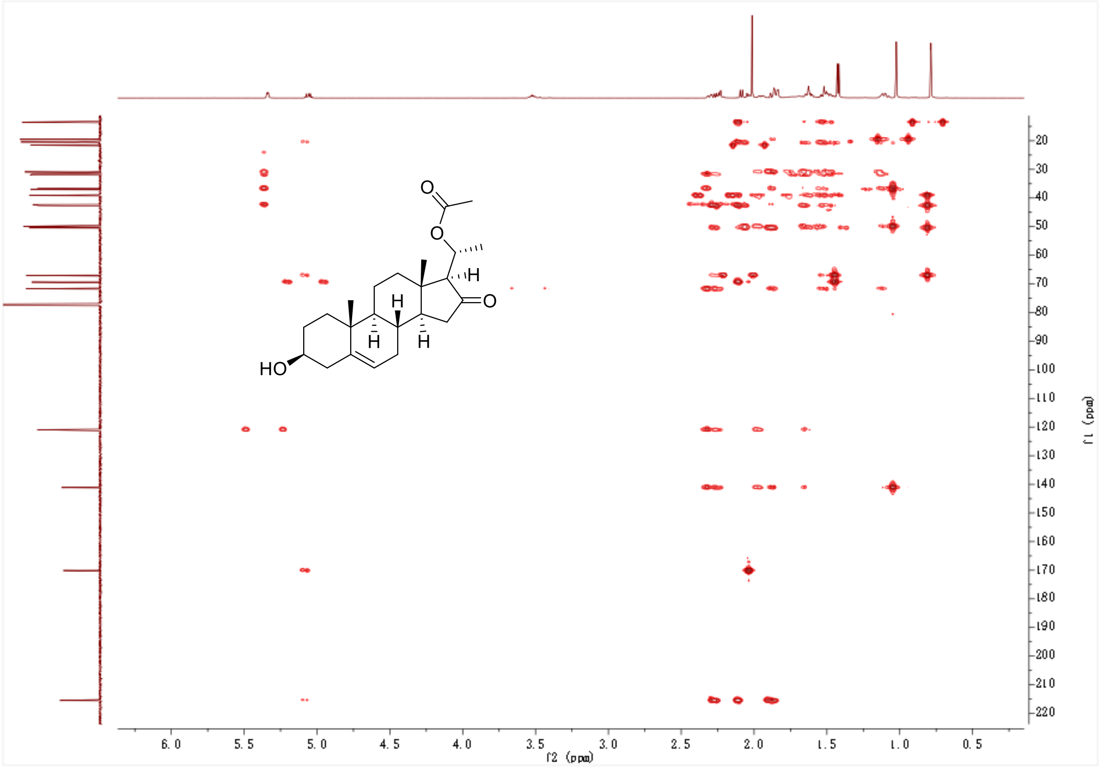

Figure S4. HMBC spectrum of compound **1** in $CDCl_3$

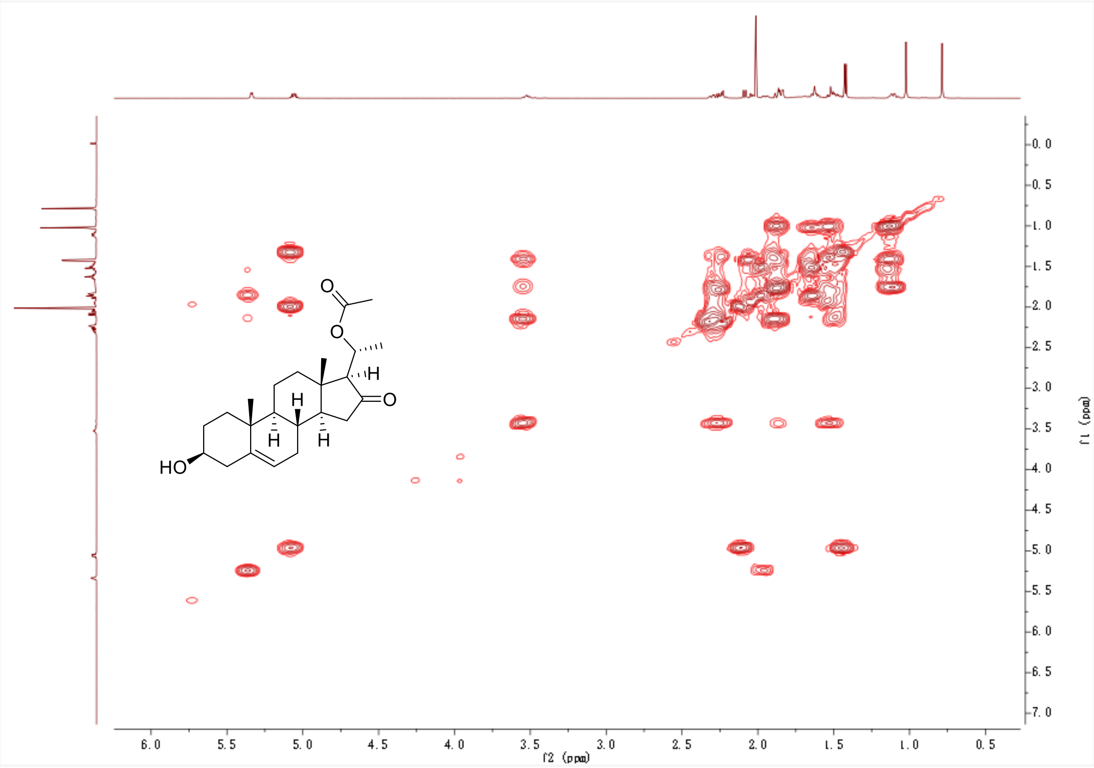

Figure S5. ^{1}H-^{1}H COSY spectrum of compound **1** in $CDCl_3$

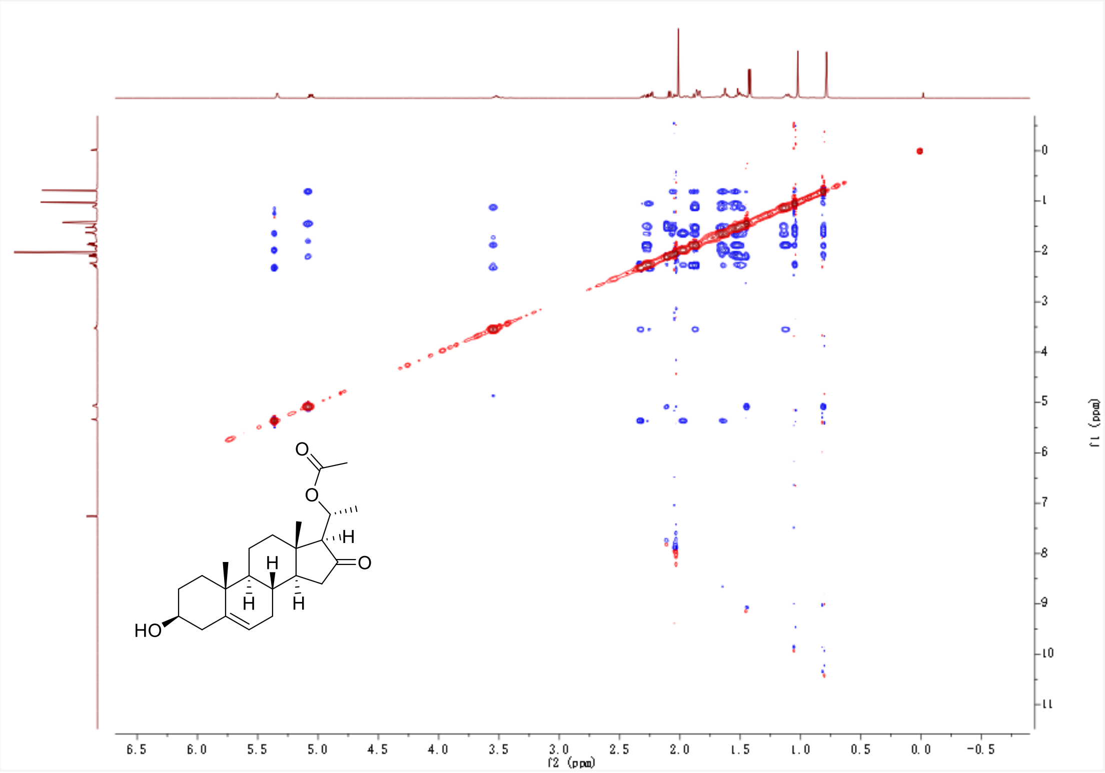

Figure S6. NOESY spectrum of compound **1** in $CDCl_3$

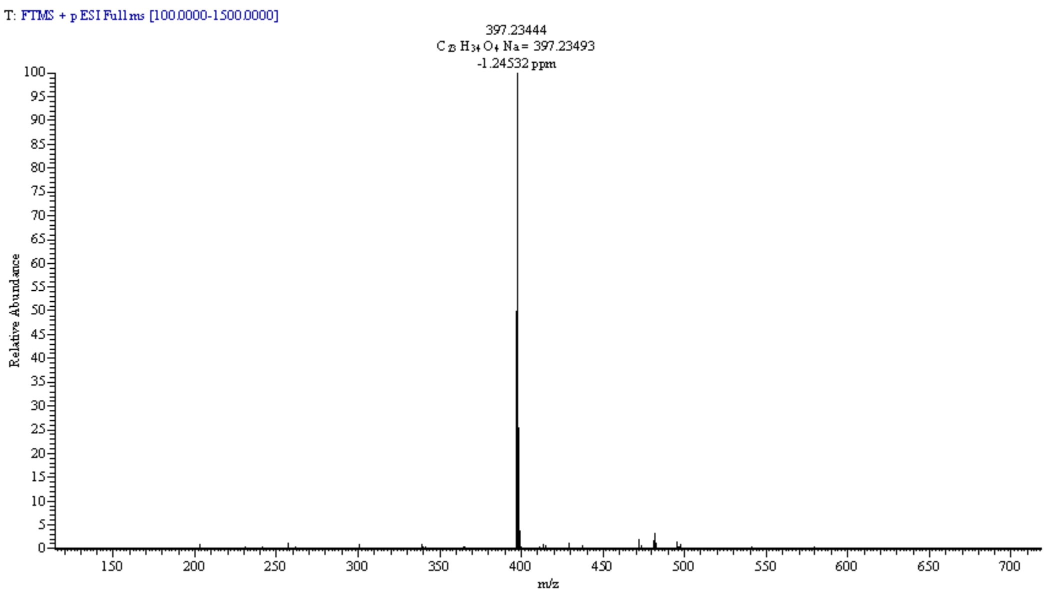

Figure S7. HRESIMS spectrum of compound **1**

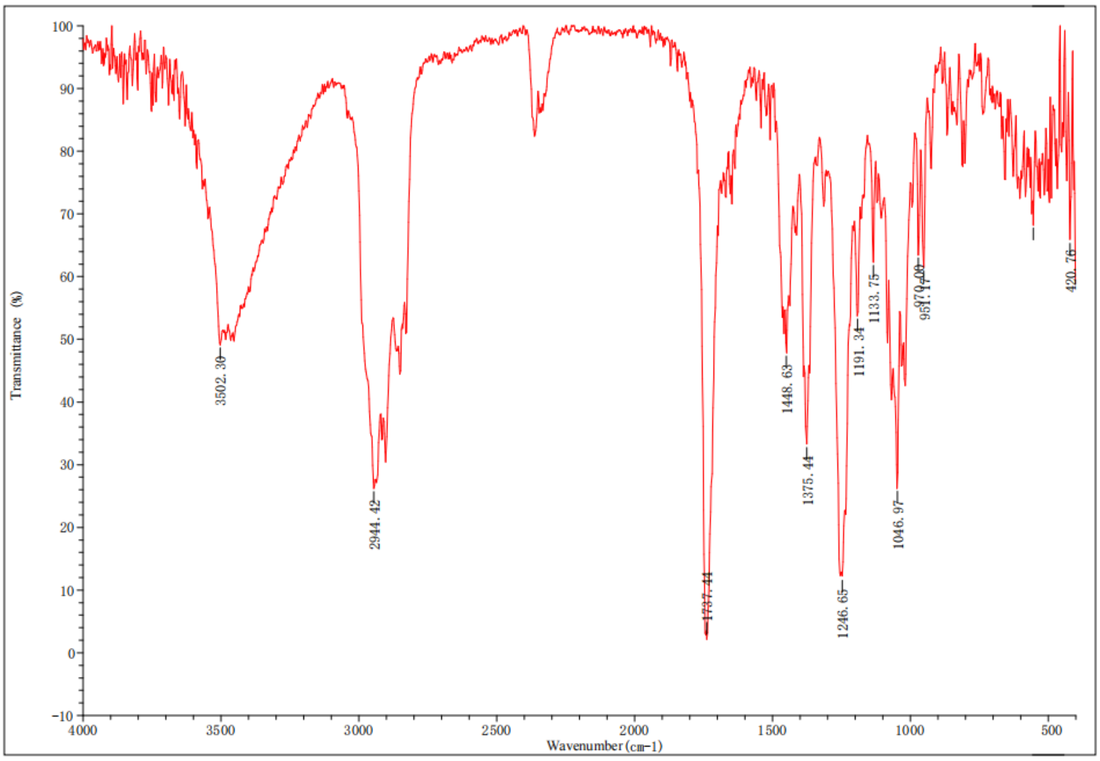

Figure S8. IR spectrum of compound **1**

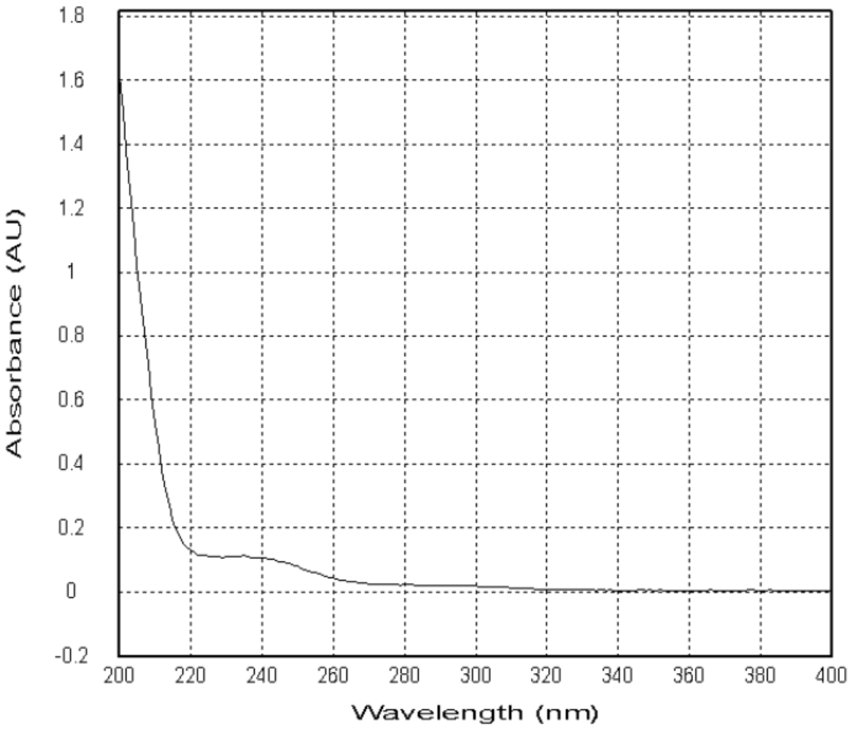

Figure S9. UV spectrum of compound **1**

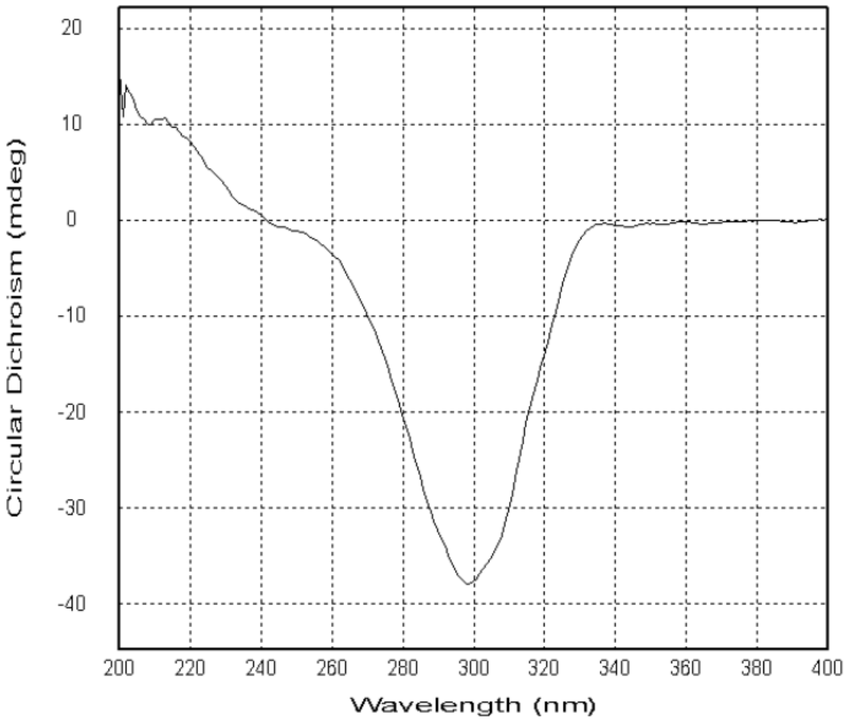

Figure S10. CD spectrum of compound **1**

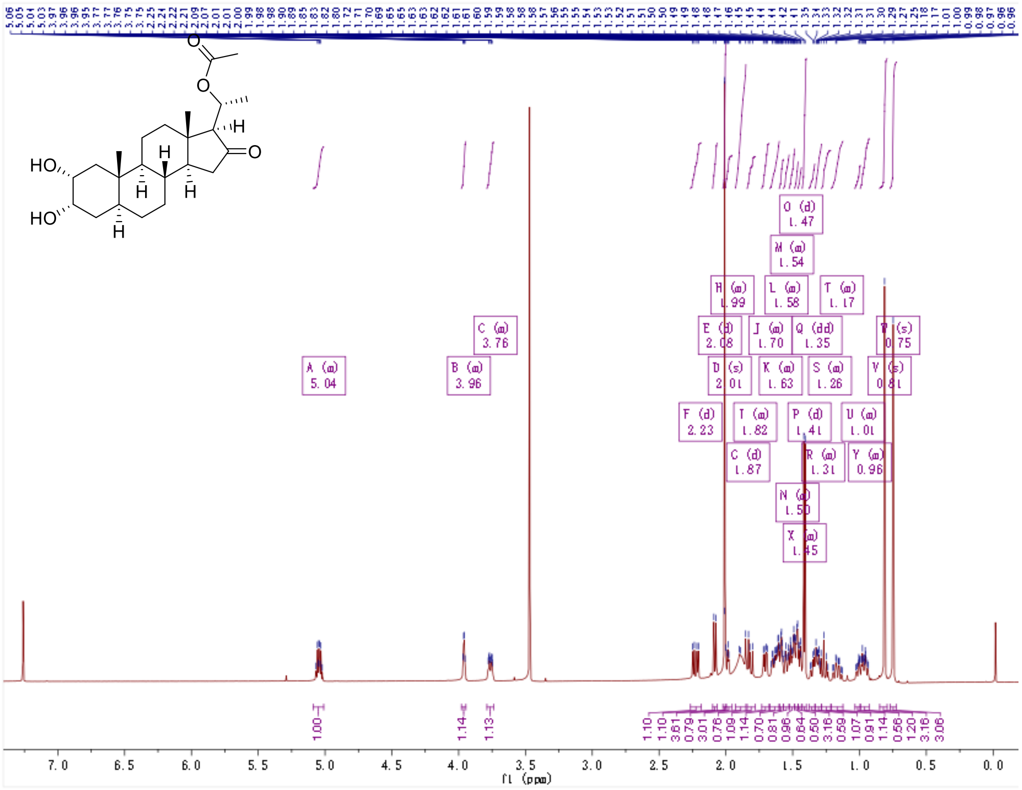

Figure S11. ^{1}H NMR spectrum of compound **2** in $CDCl_3$ (600 MHz)

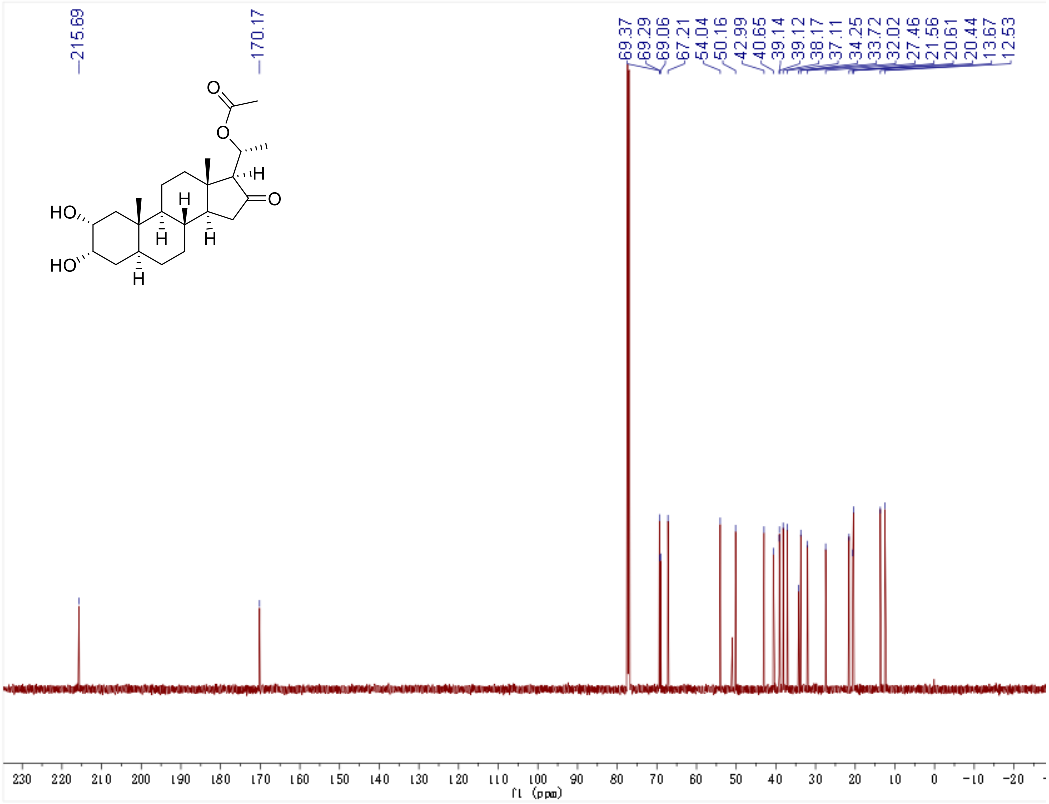

Figure S12. ^{13}C NMR spectrum of compound **2** in $CDCl_3$ (150 MHz)

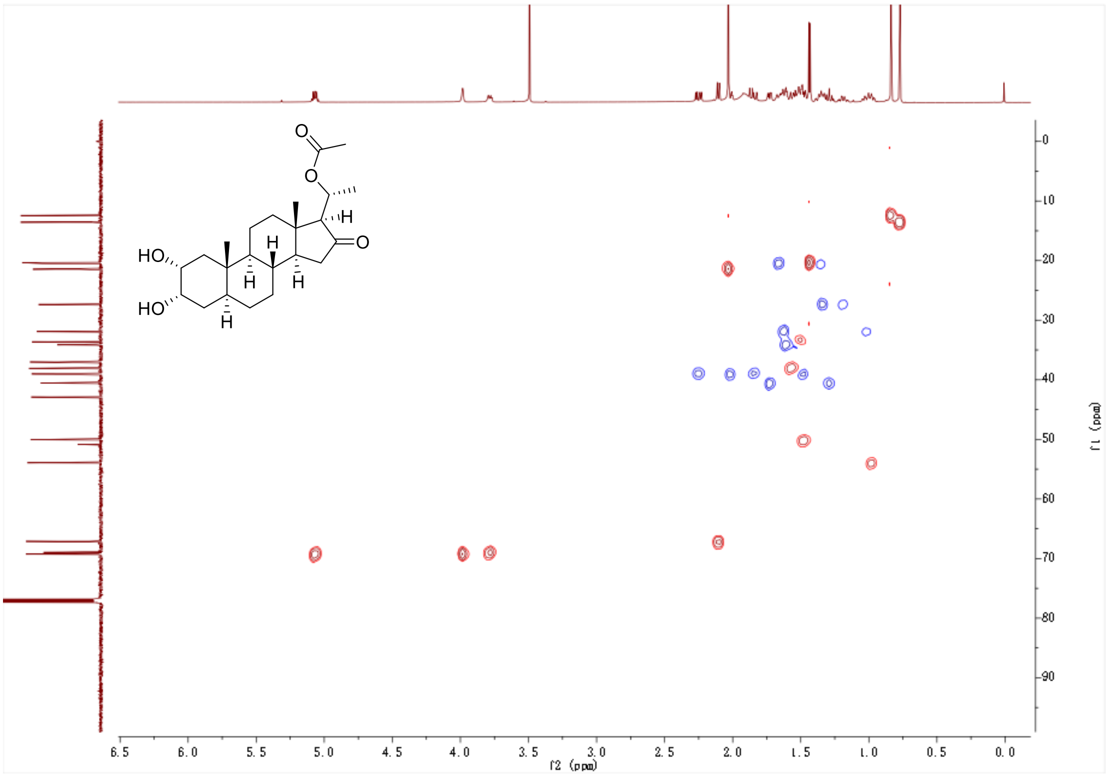

Figure S13. HSQC spectrum of compound **2** in $CDCl_3$

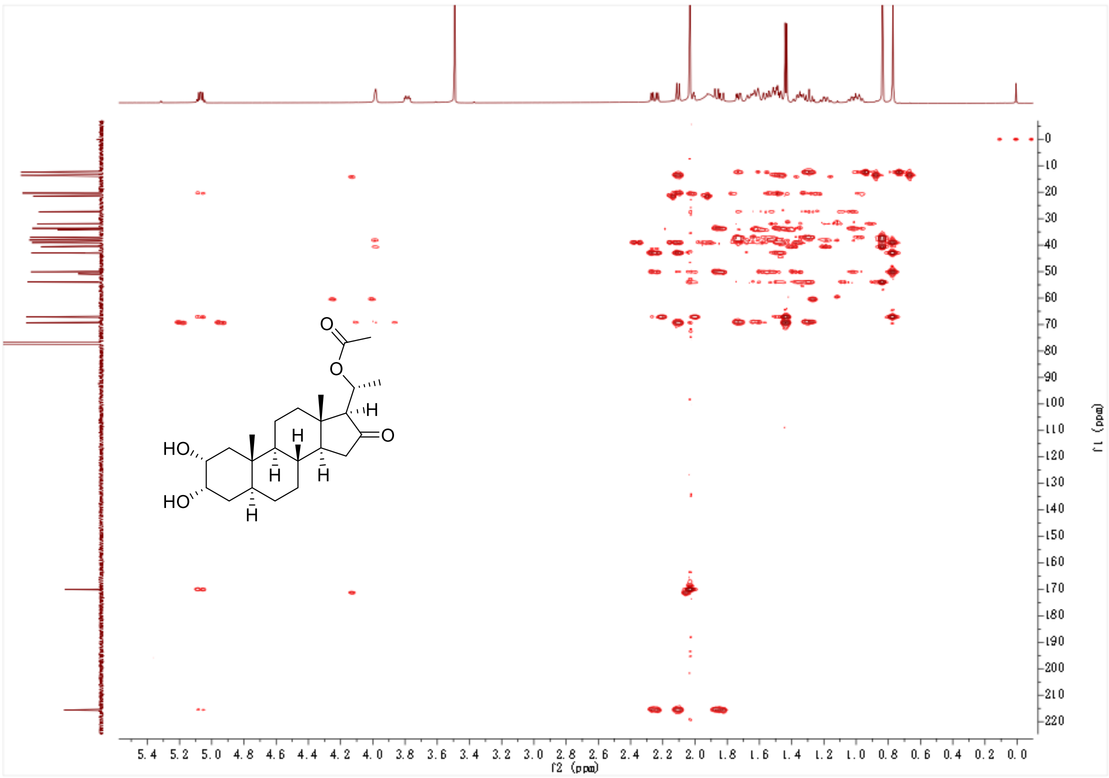

Figure S14. HMBC spectrum of compound **2** in $CDCl_3$

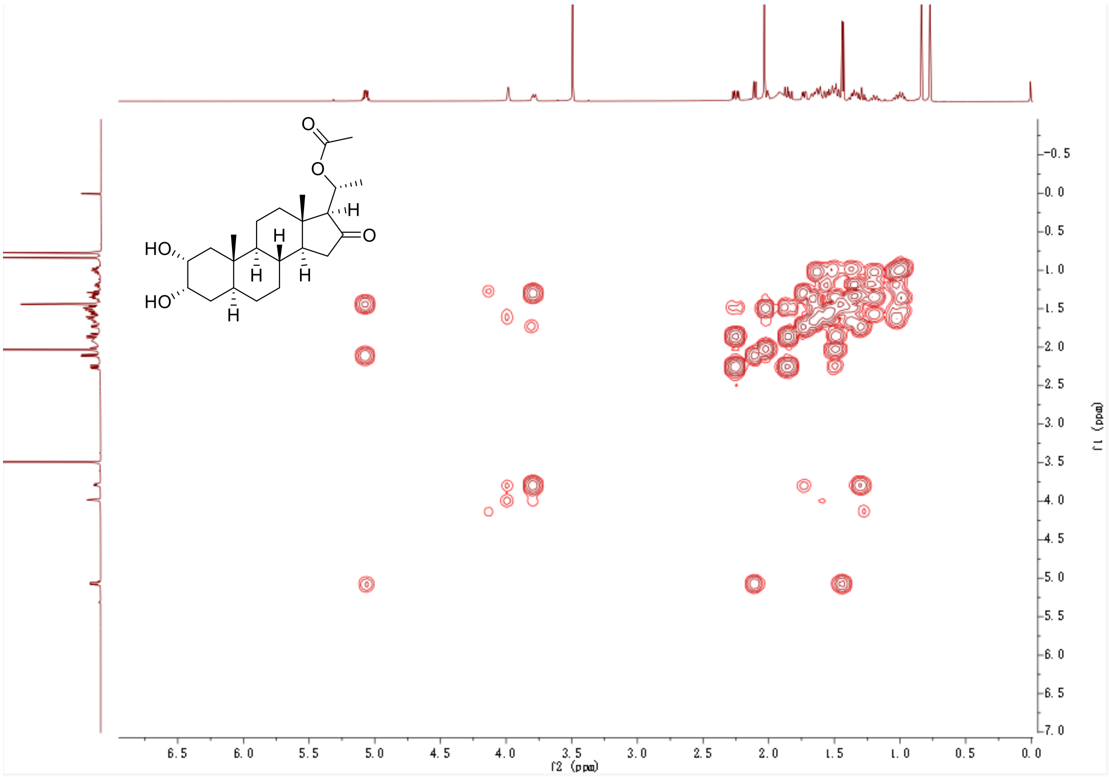

Figure S15. ^{1}H-^{1}H COSY spectrum of compound **2** in $CDCl_3$

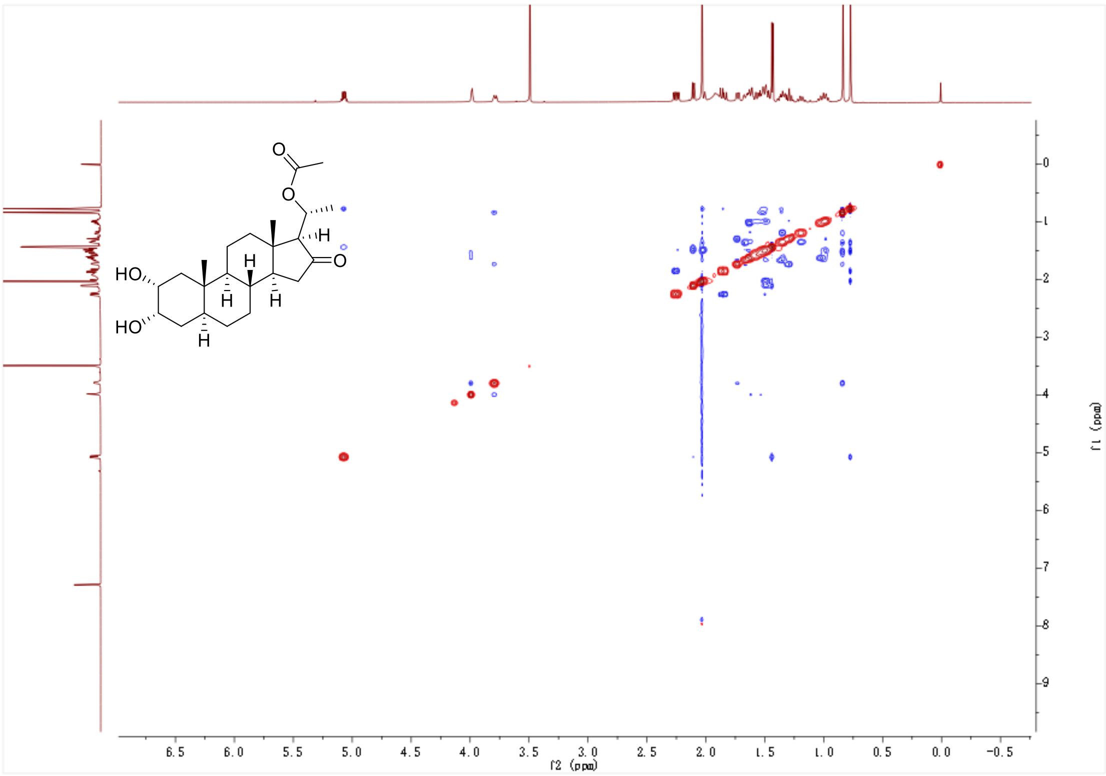

Figure S16. NOESY spectrum of compound **2** in $CDCl_3$

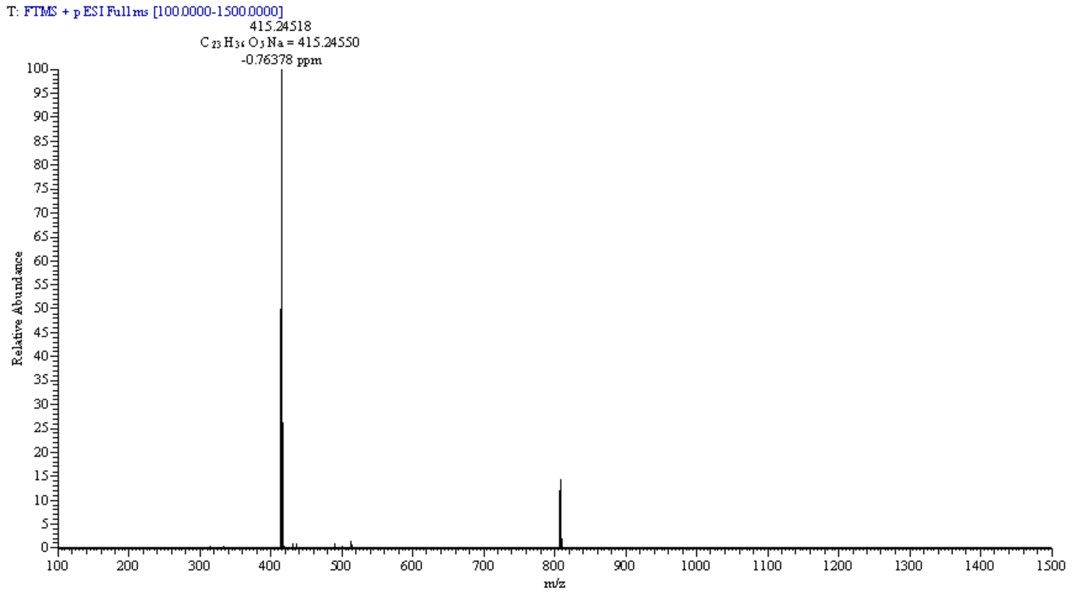

Figure S17. HRESIMS spectrum of compound **2**

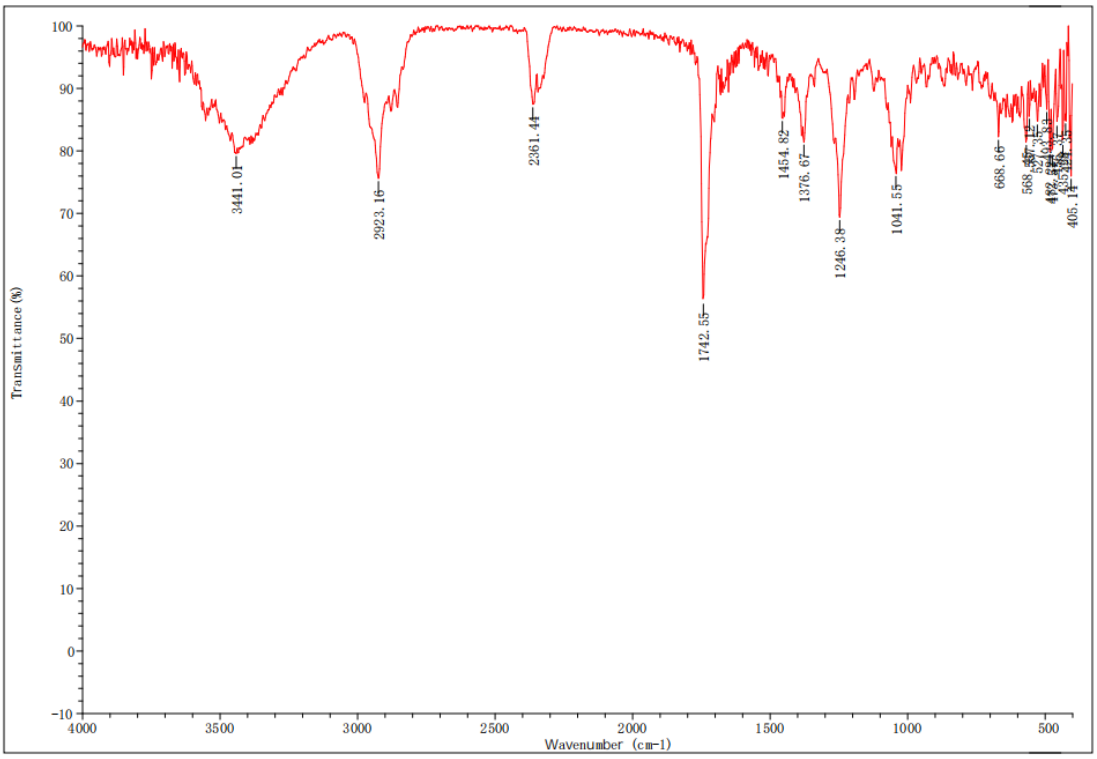

Figure S18. IR spectrum of compound **2**

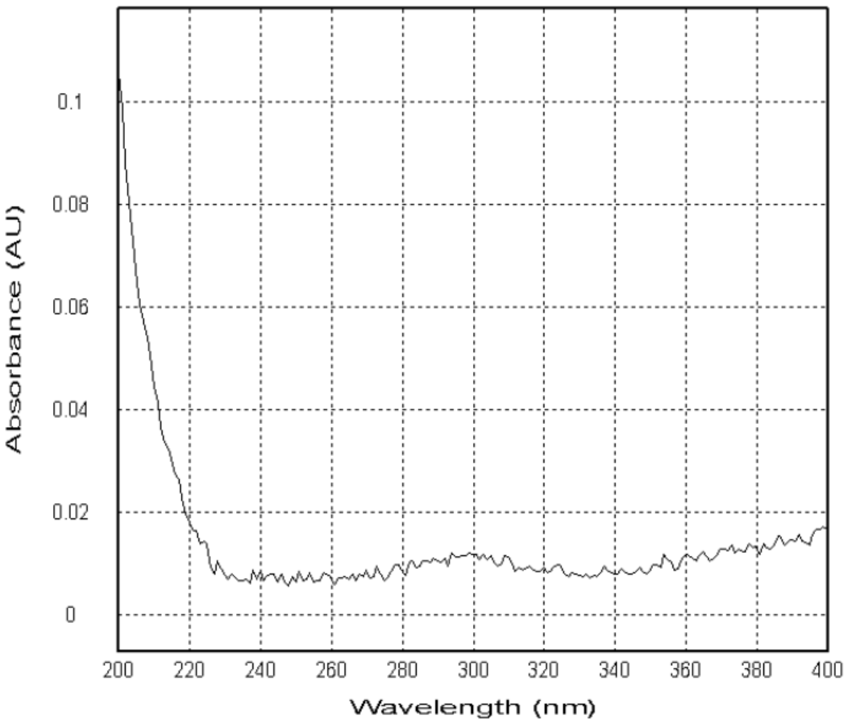

Figure S19. UV spectrum of compound **2**

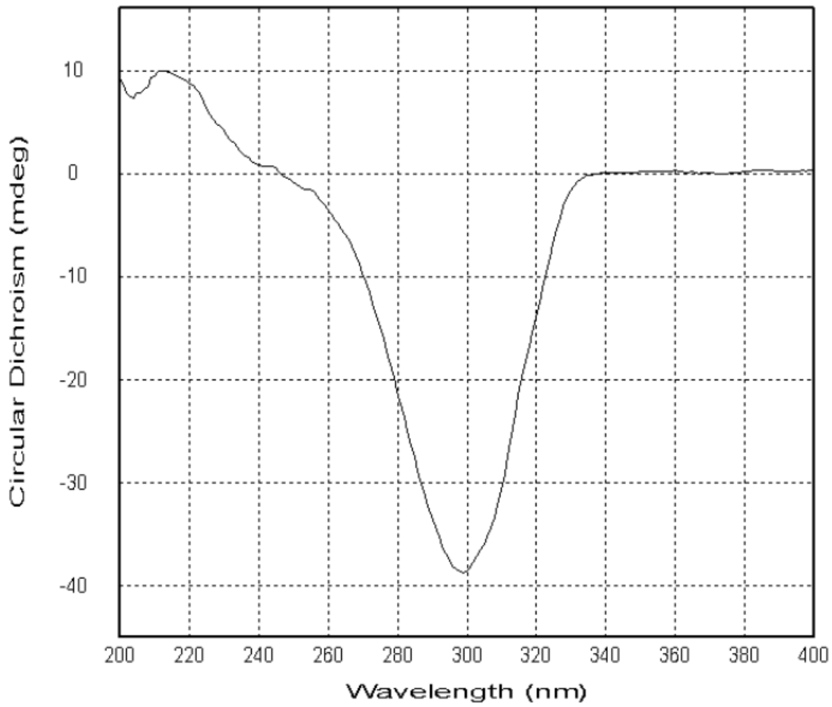

Figure S20. CD spectrum of compound **2**

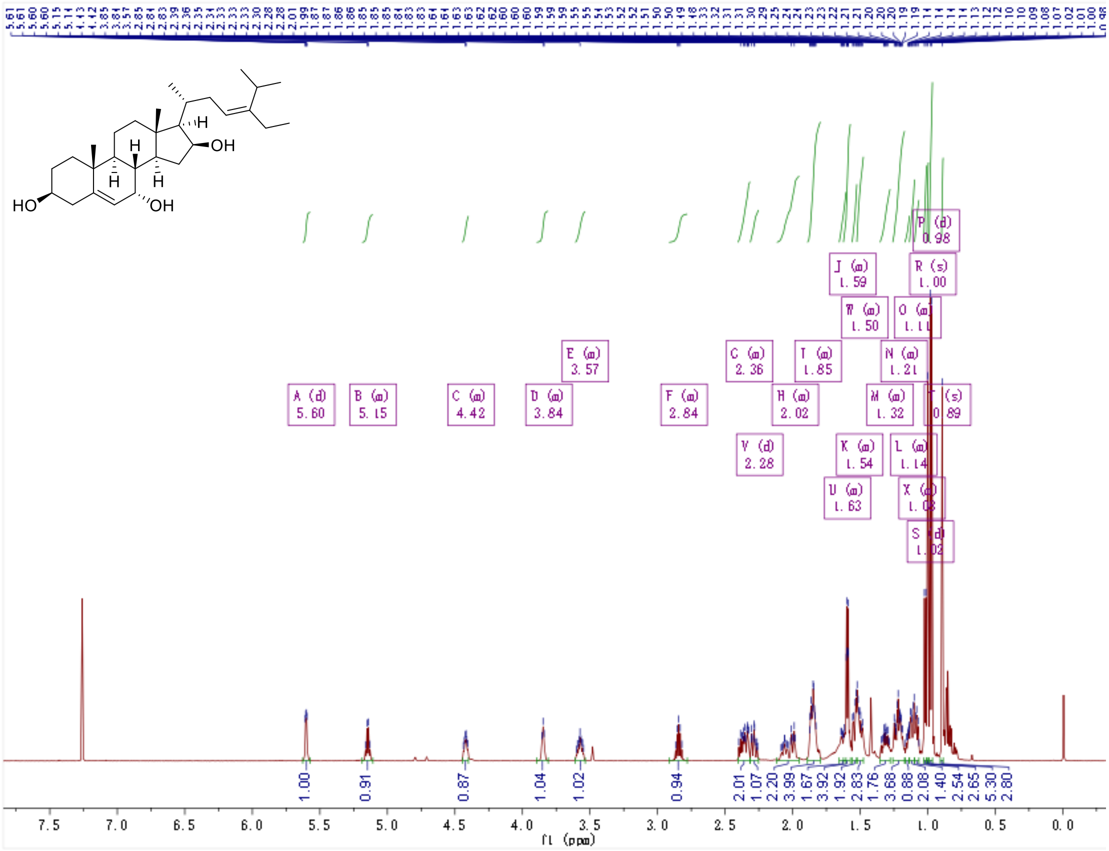

Figure S21. ^{1}H NMR spectrum of compound **3** in $CDCl_3$ (600 MHz)

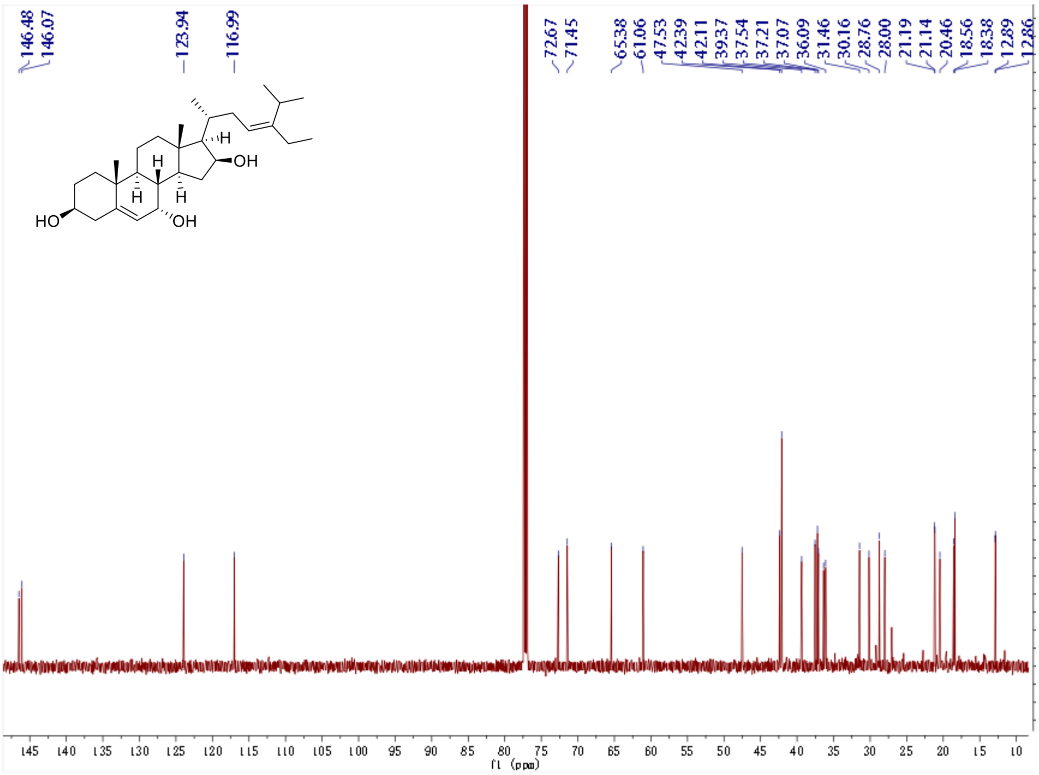

Figure S22. ^{13}C NMR spectrum of compound **3** in $CDCl_3$ (150 MHz)

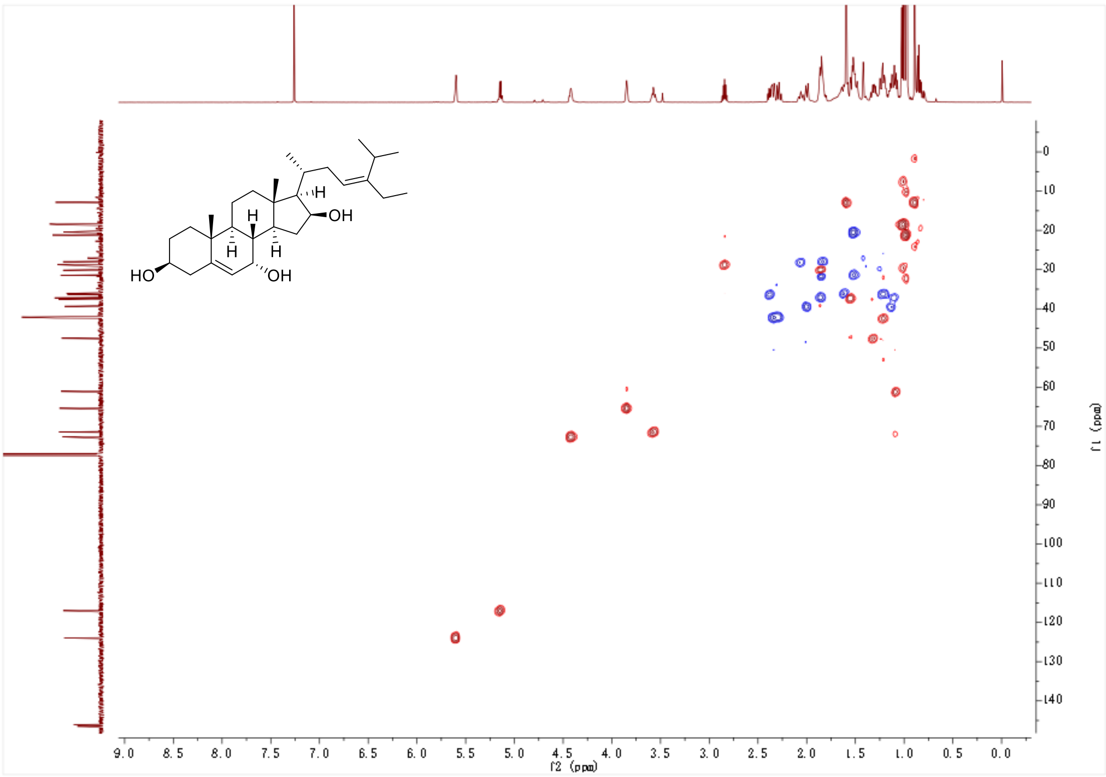

Figure S23. HSQC spectrum of compound **3** in $CDCl_3$

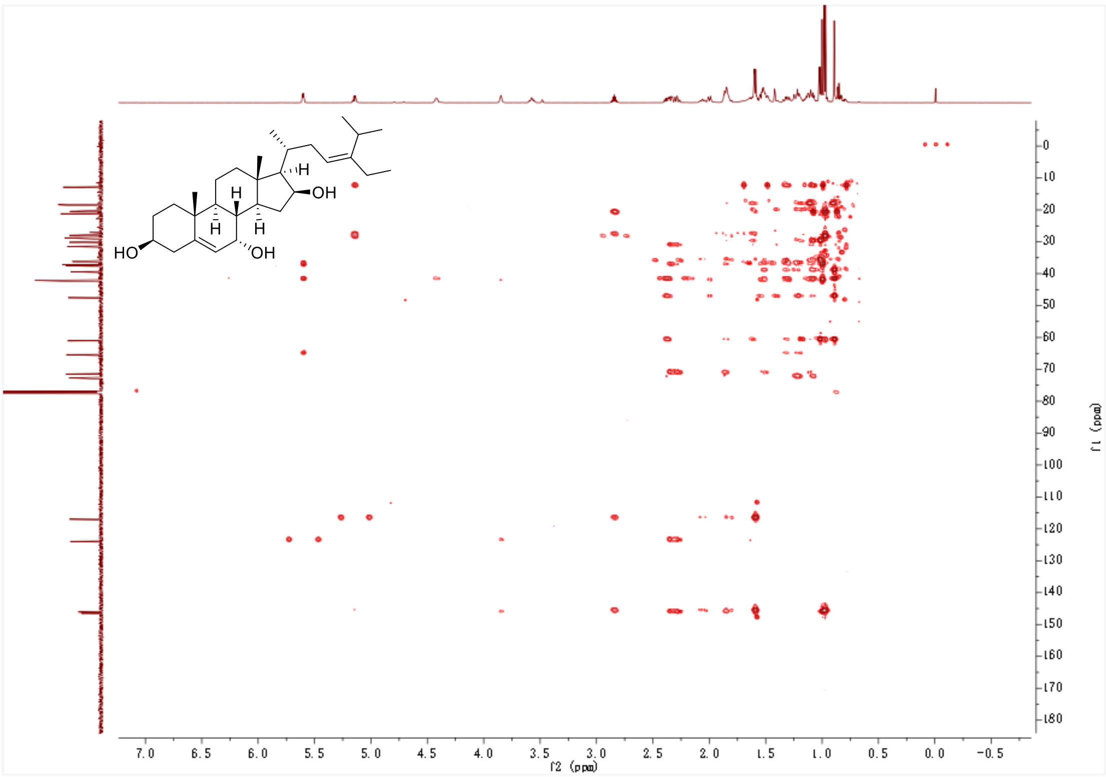

Figure S24. HMBC spectrum of compound **3** in $CDCl_3$

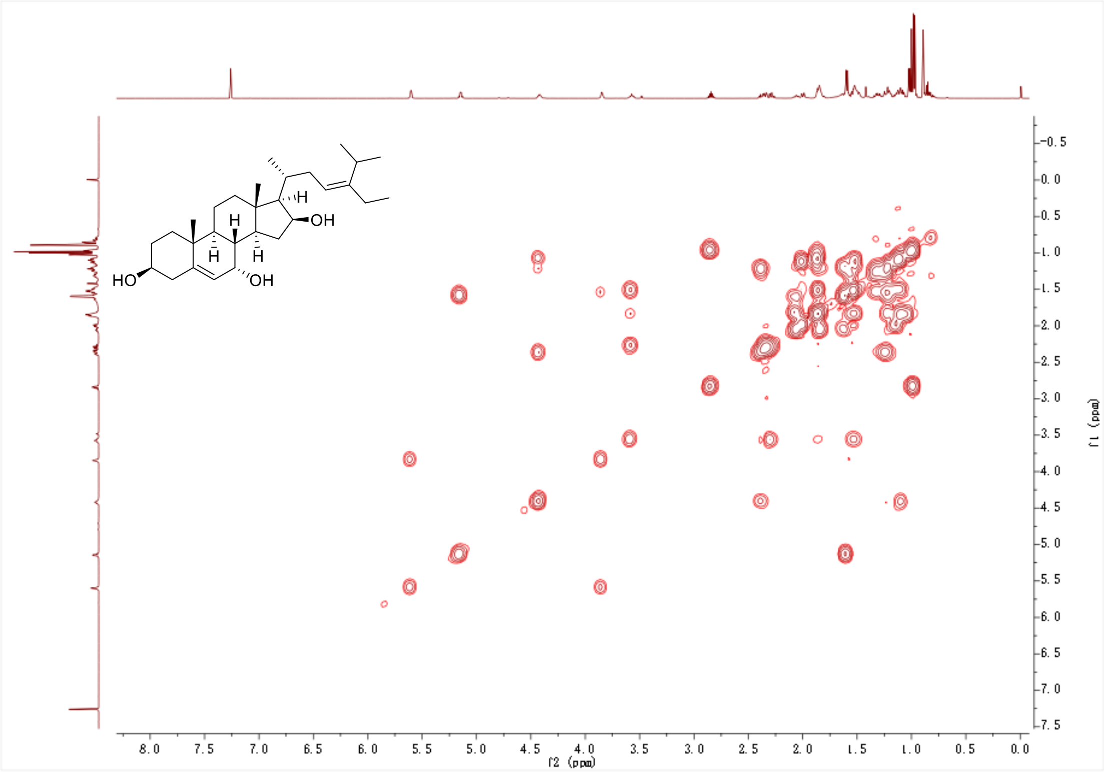

Figure S25. ^{1}H-^{1}H COSY spectrum of compound **3** in $CDCl_3$

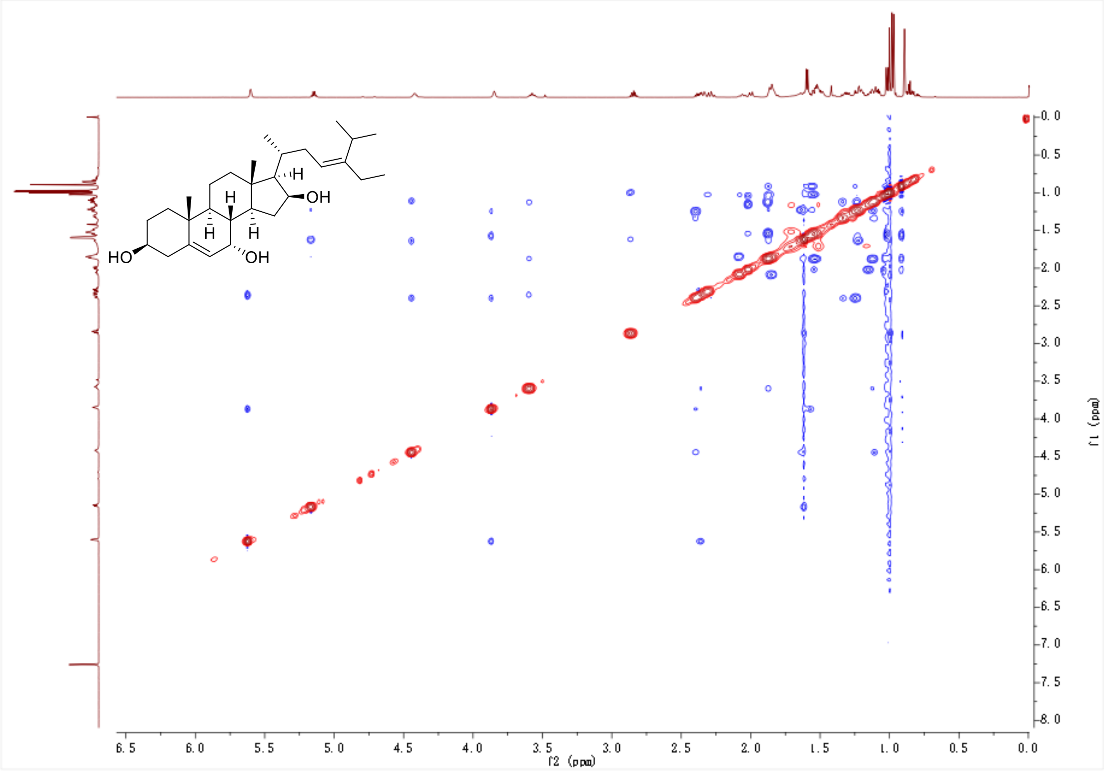

Figure S26. NOESY spectrum of compound **3** in $CDCl_3$

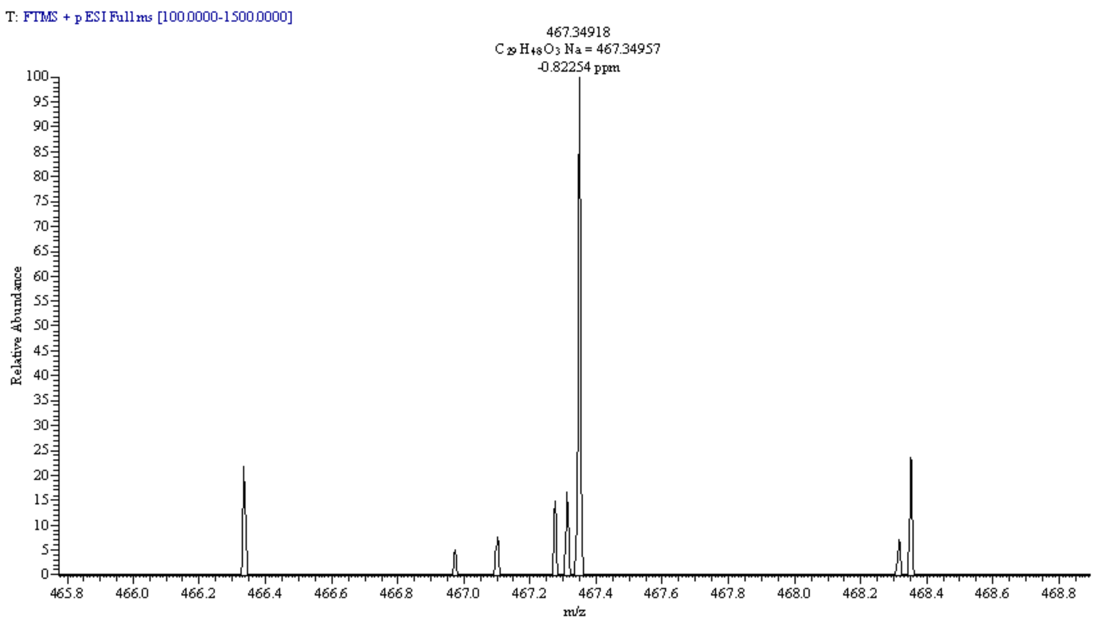

Figure S27. HRESIMS spectrum of compound **3**

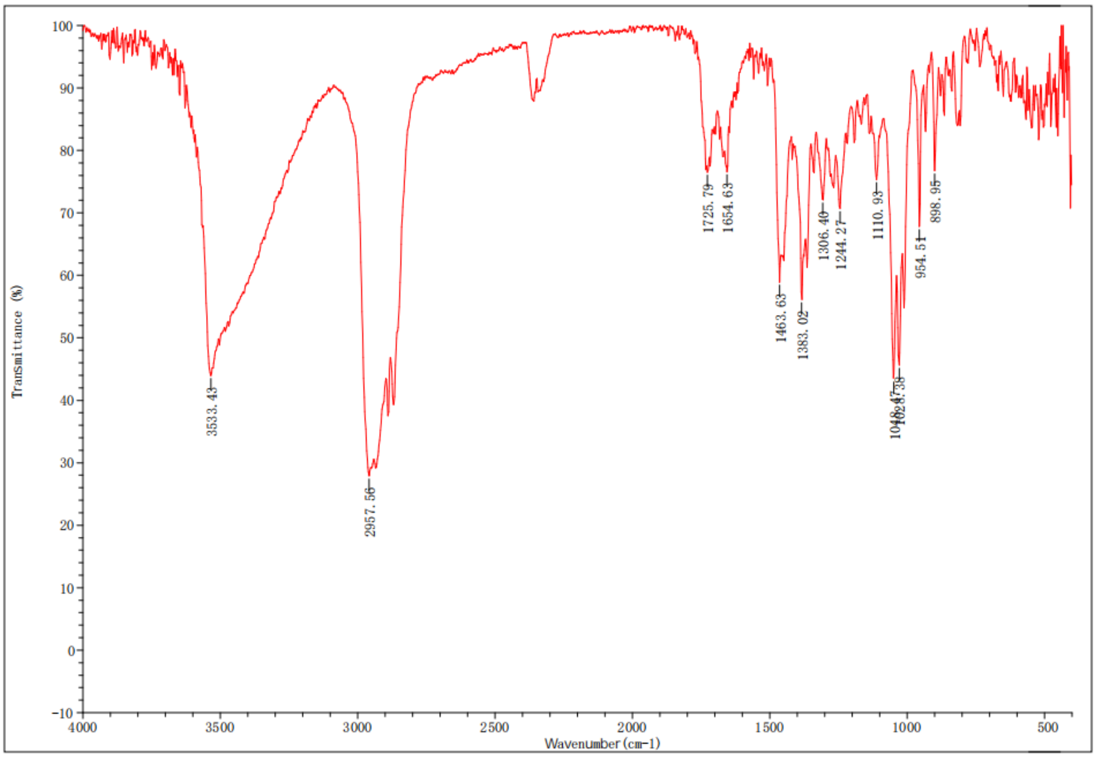

Figure S28. IR spectrum of compound **3**

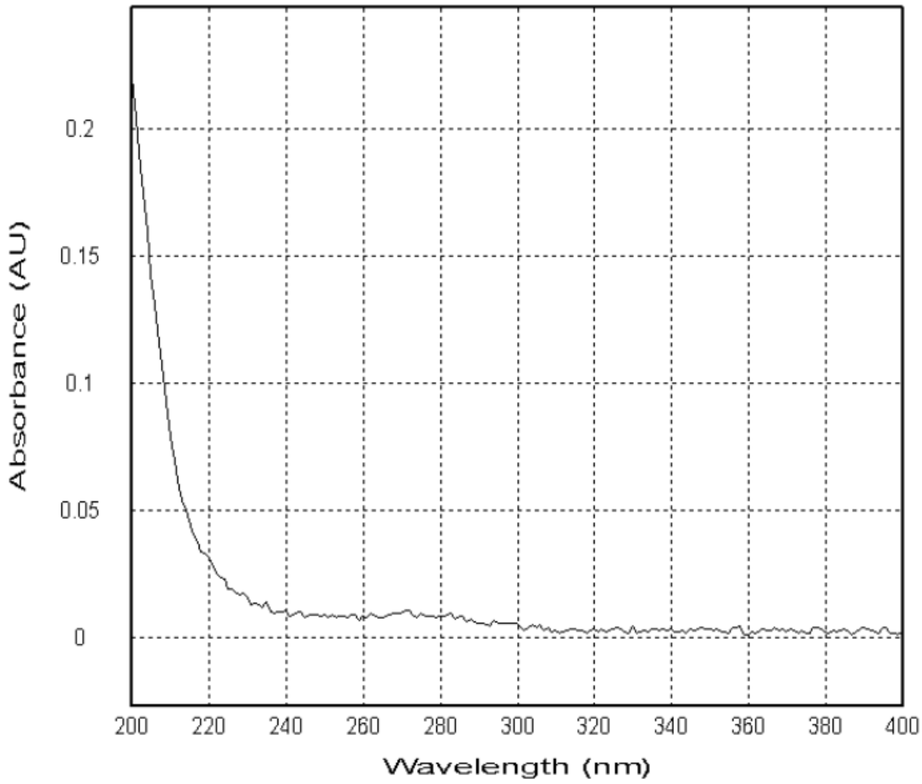

Figure S29. UV spectrum of compound **3**

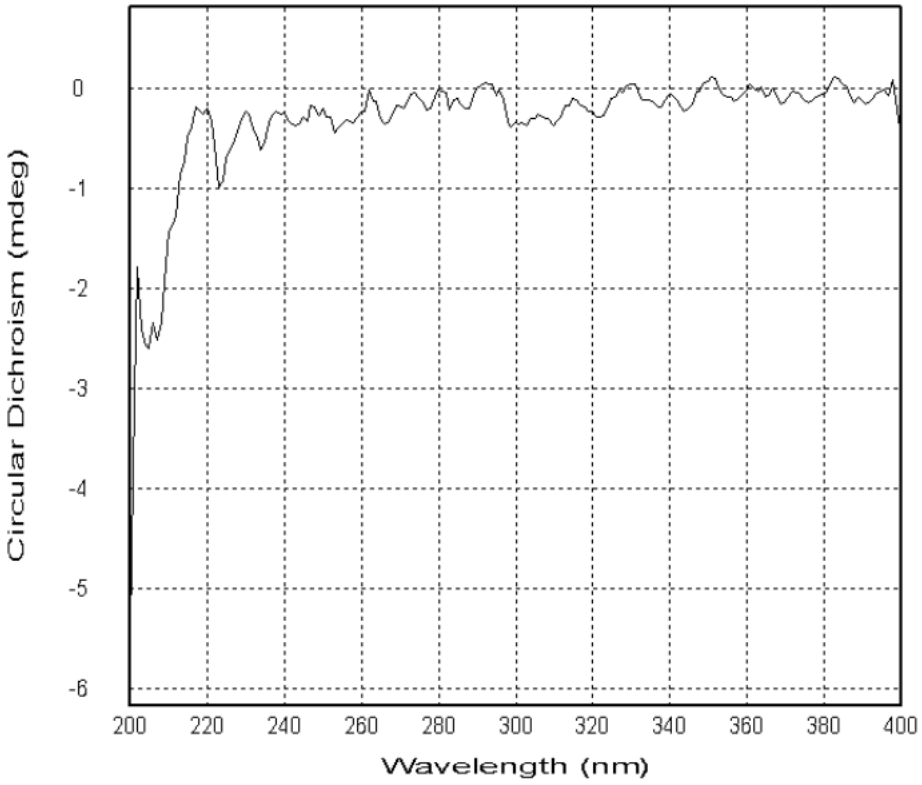

Figure S30. CD spectrum of compound **3**

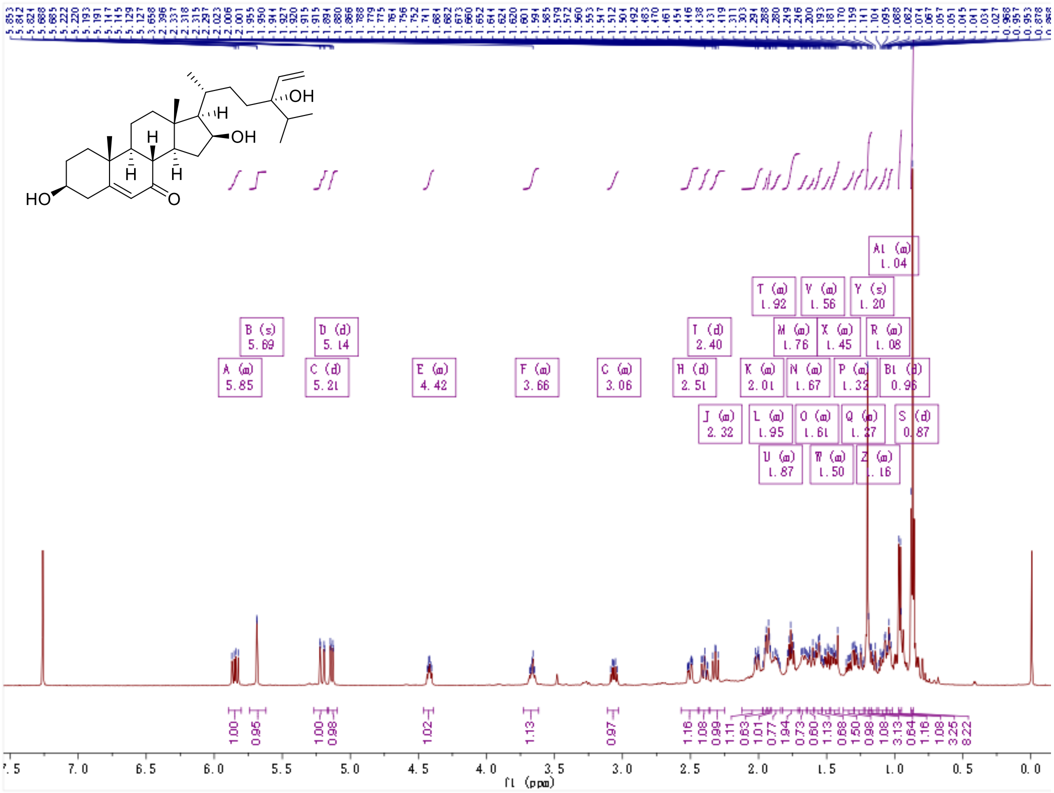

Figure S31. ^{1}H NMR spectrum of compound **4** in $CDCl_3$ (600 MHz)

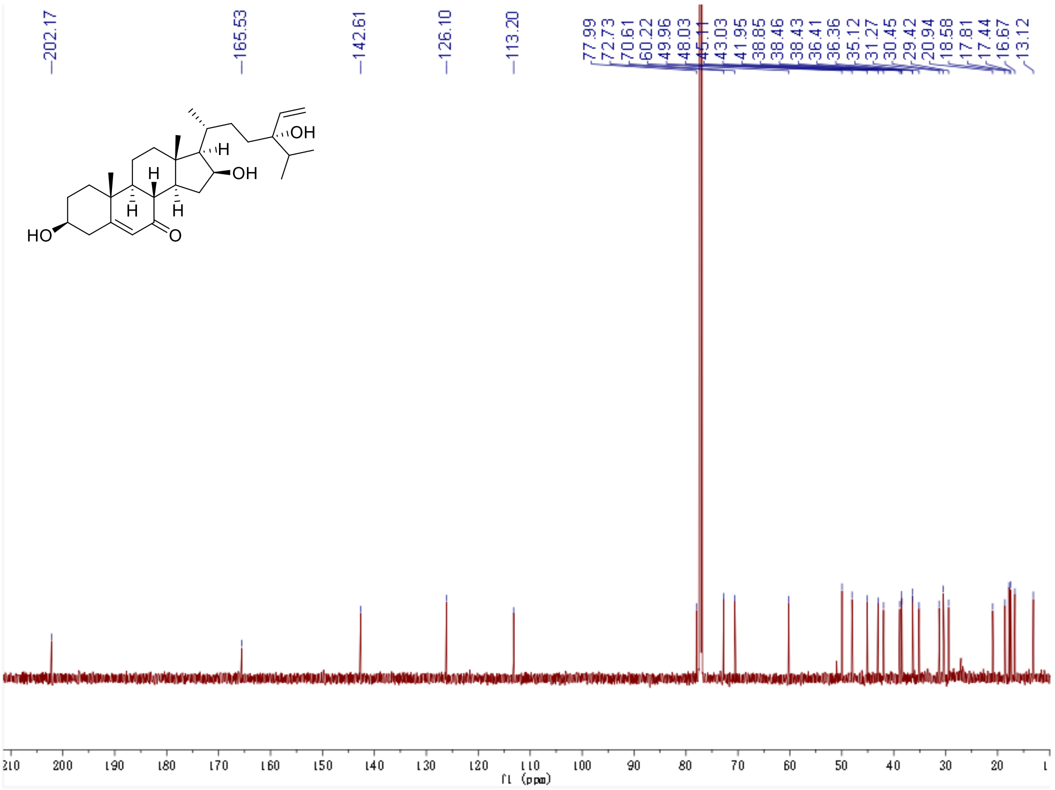

Figure S32. ^{13}C NMR spectrum of compound **4** in $CDCl_3$ (150 MHz)

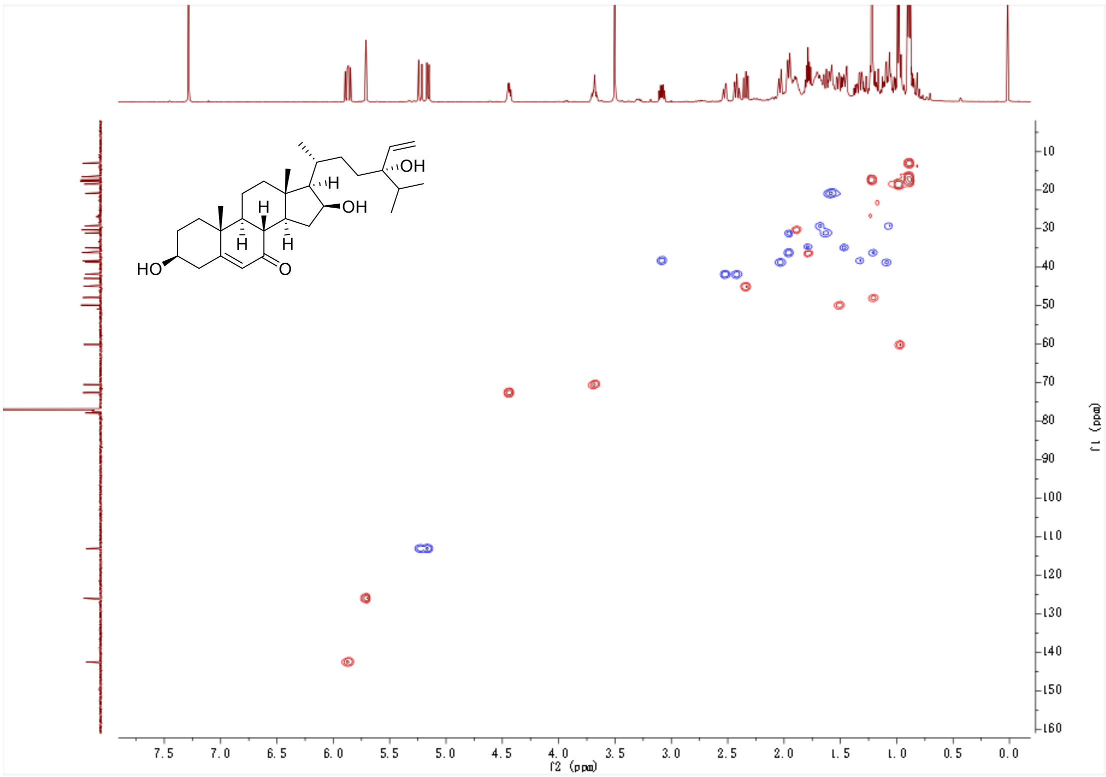

Figure S33. HSQC spectrum of compound **4** in $CDCl_3$

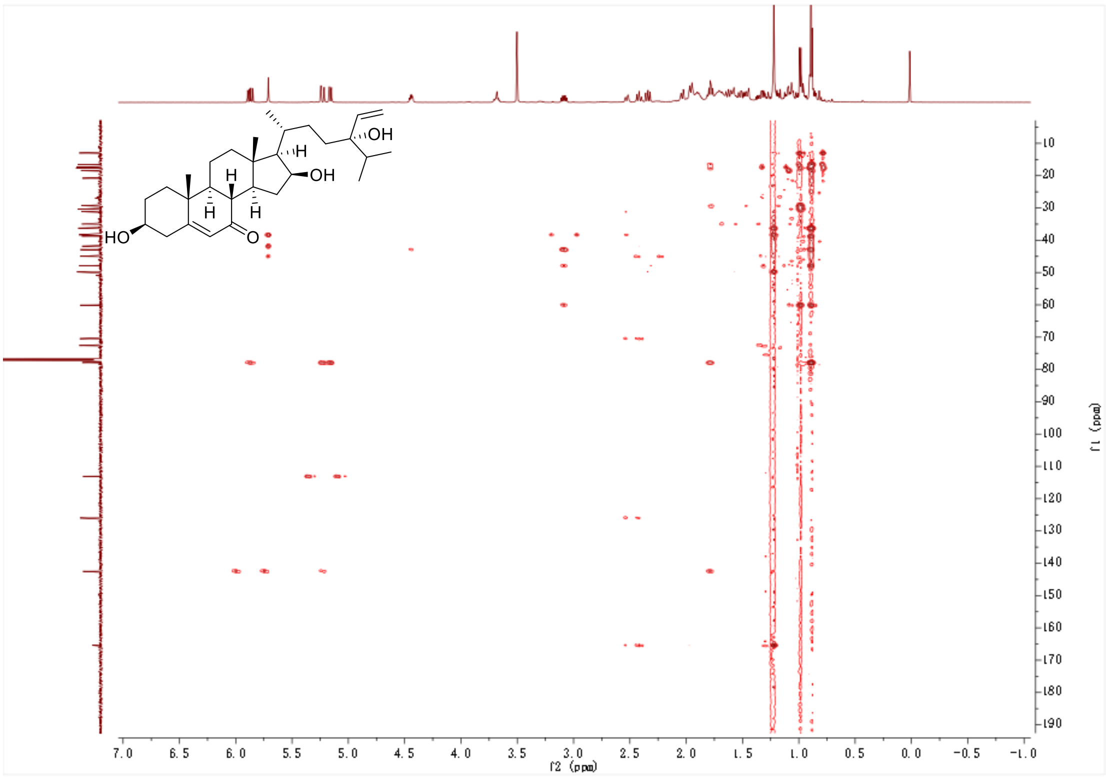

Figure S34. HMBC spectrum of compound **4** in $CDCl_3$

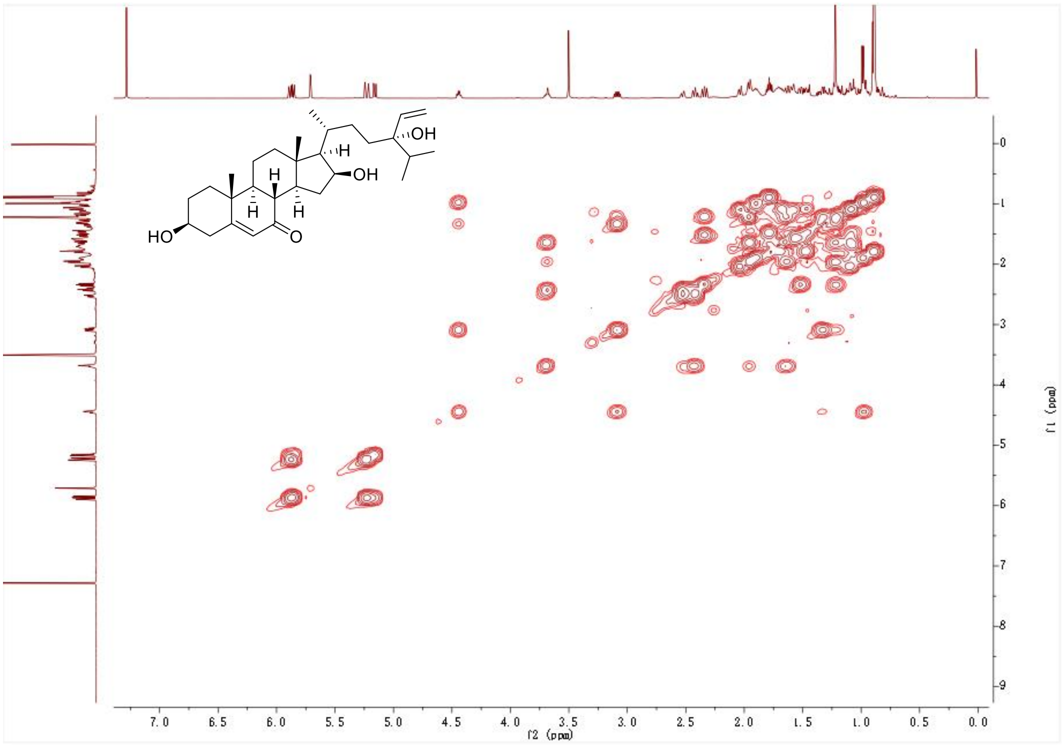

Figure S35. ^{1}H-^{1}H COSY spectrum of compound **4** in $CDCl_3$

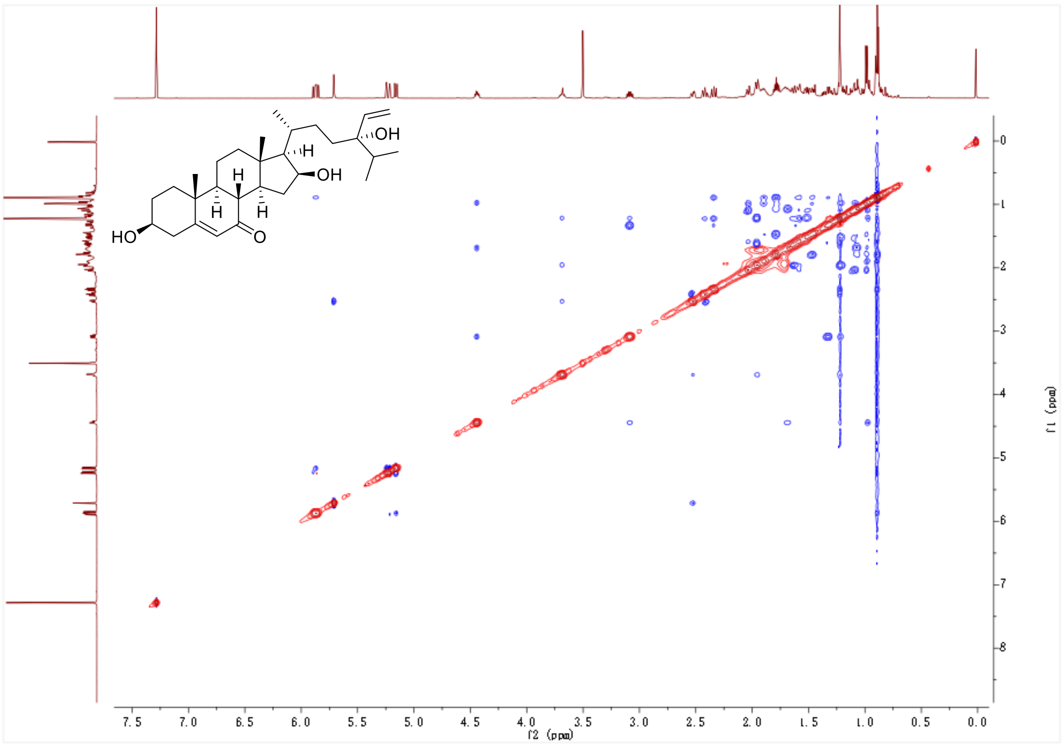

Figure S36. NOESY spectrum of compound **4** in $CDCl_3$

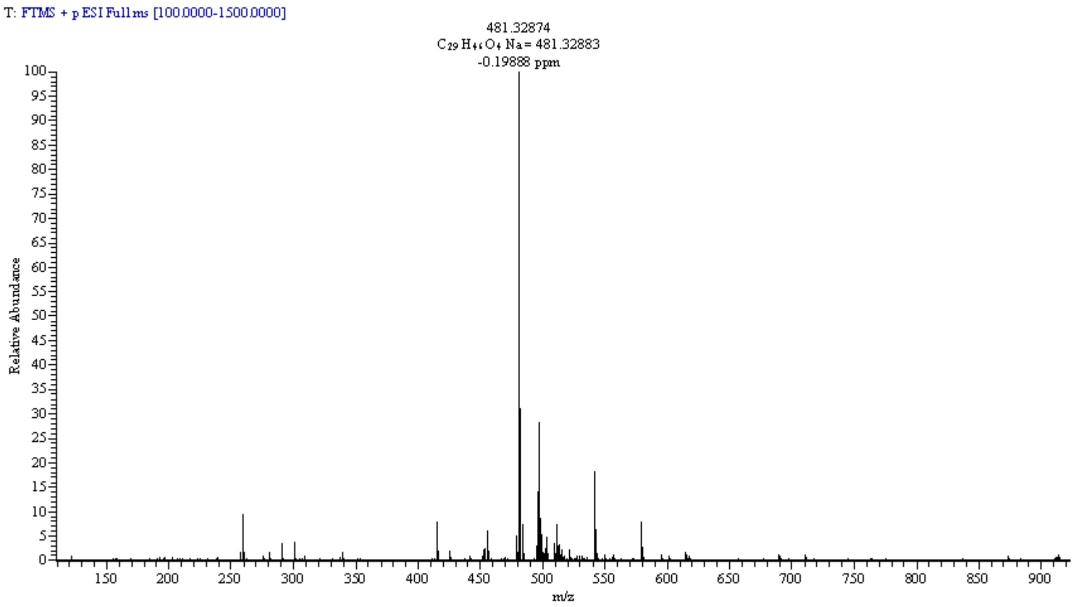

Figure S37. HRESIMS spectrum of compound **4**

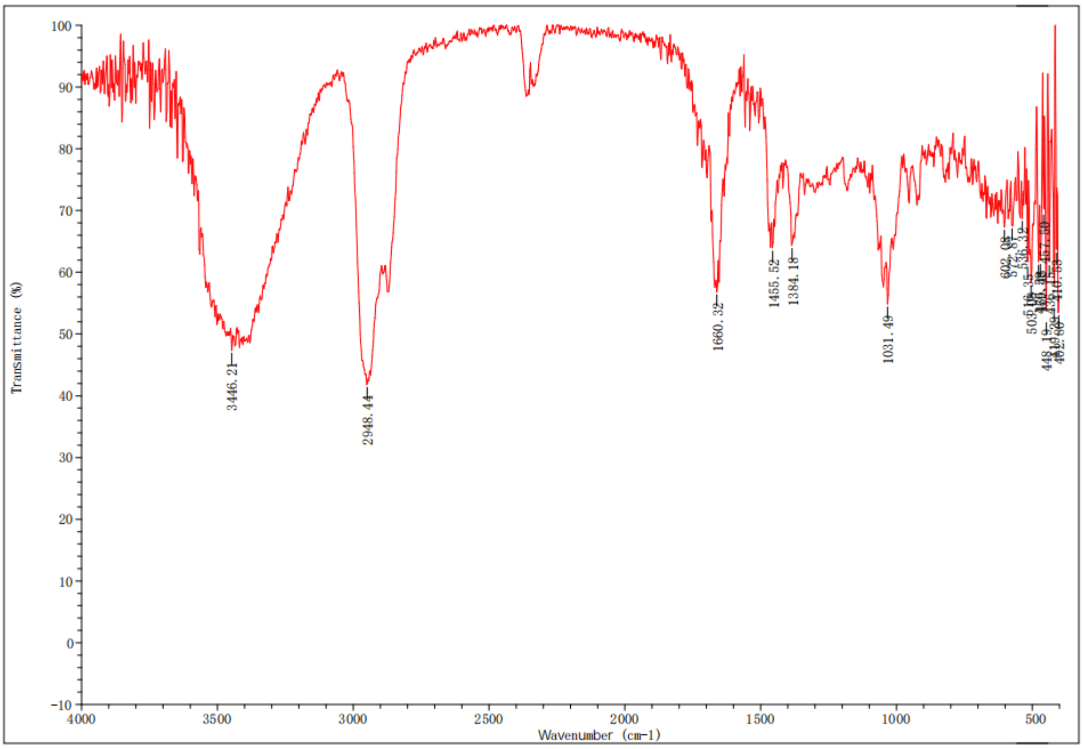

Figure S38. IR spectrum of compound **4**

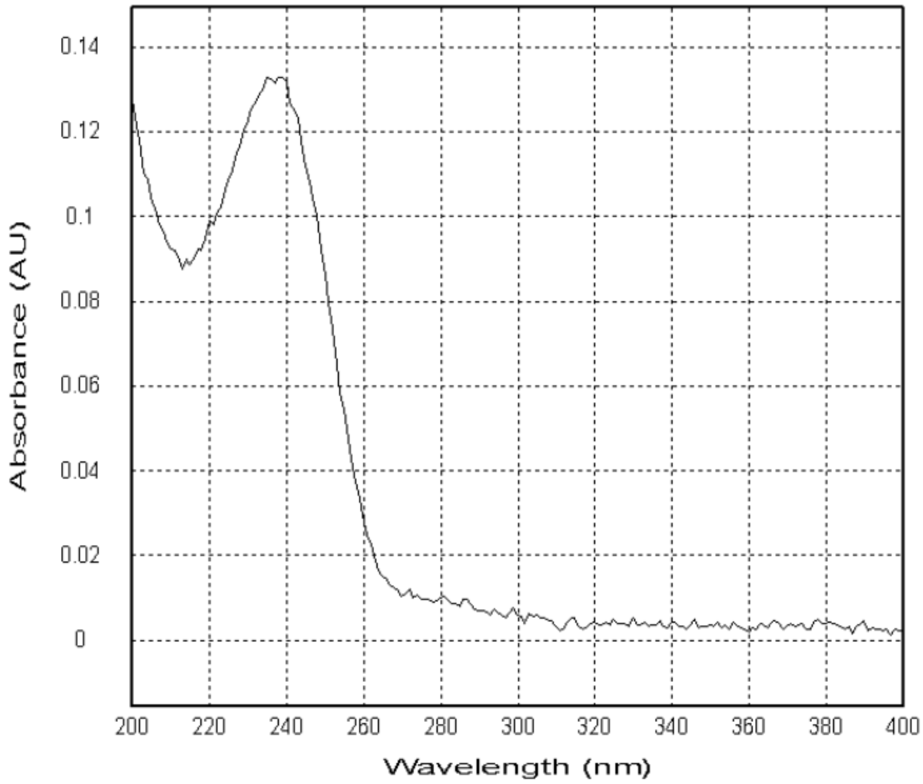

Figure S39. UV spectrum of compound **4**

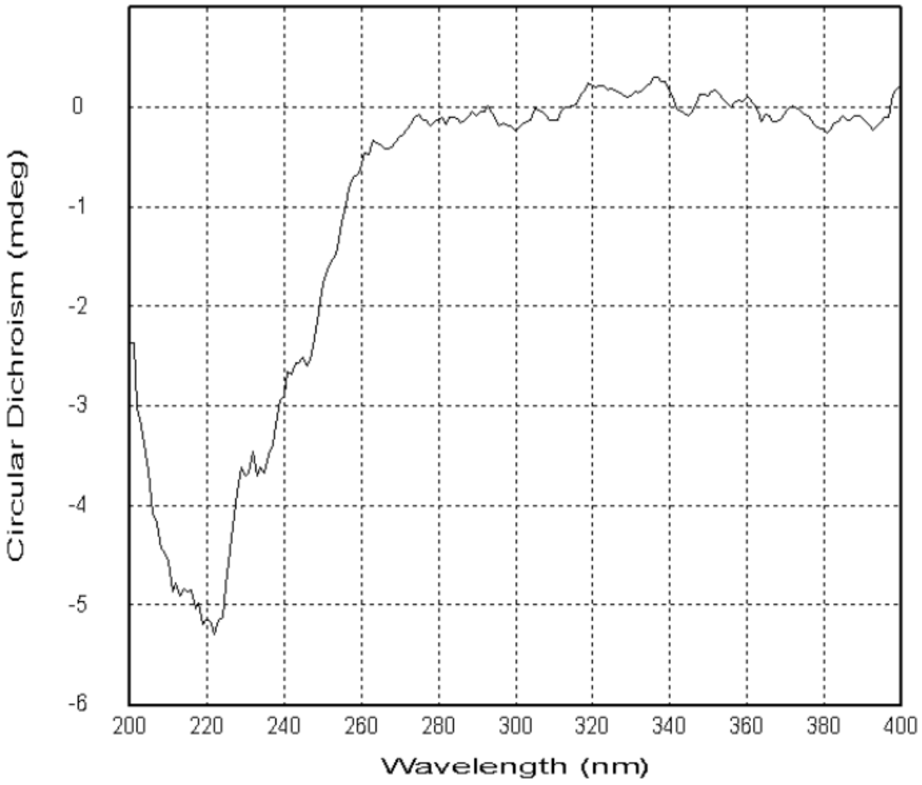

Figure S40. CD spectrum of compound **4**

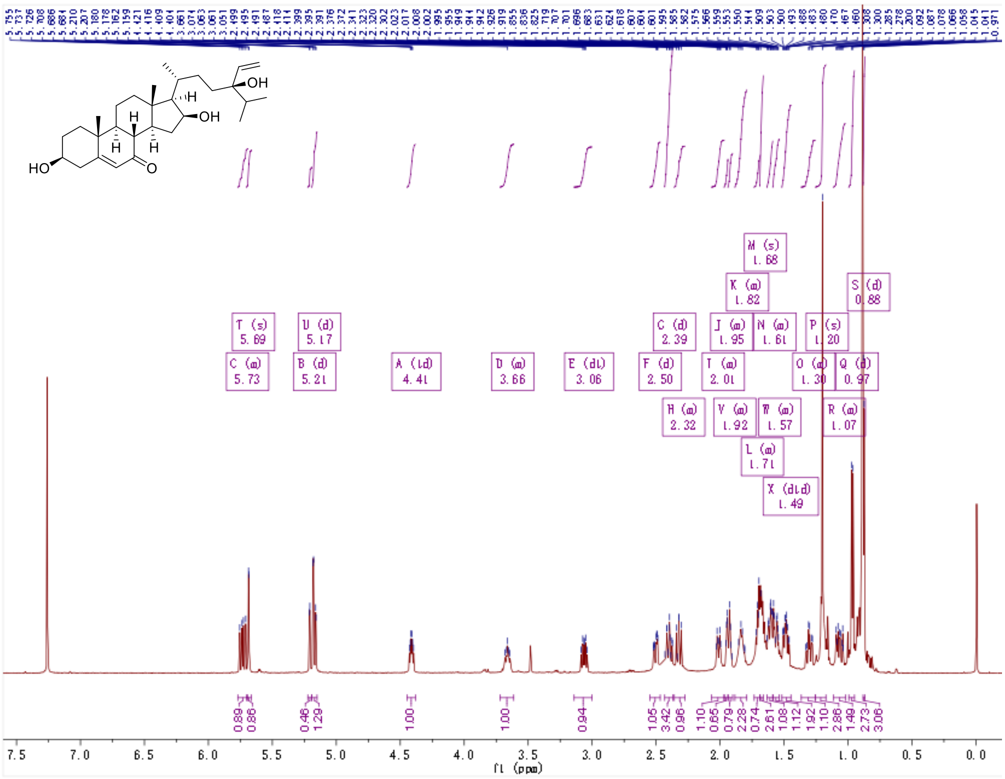

Figure S41. ^{1}H NMR spectrum of compound **5** in $CDCl_3$ (600 MHz)

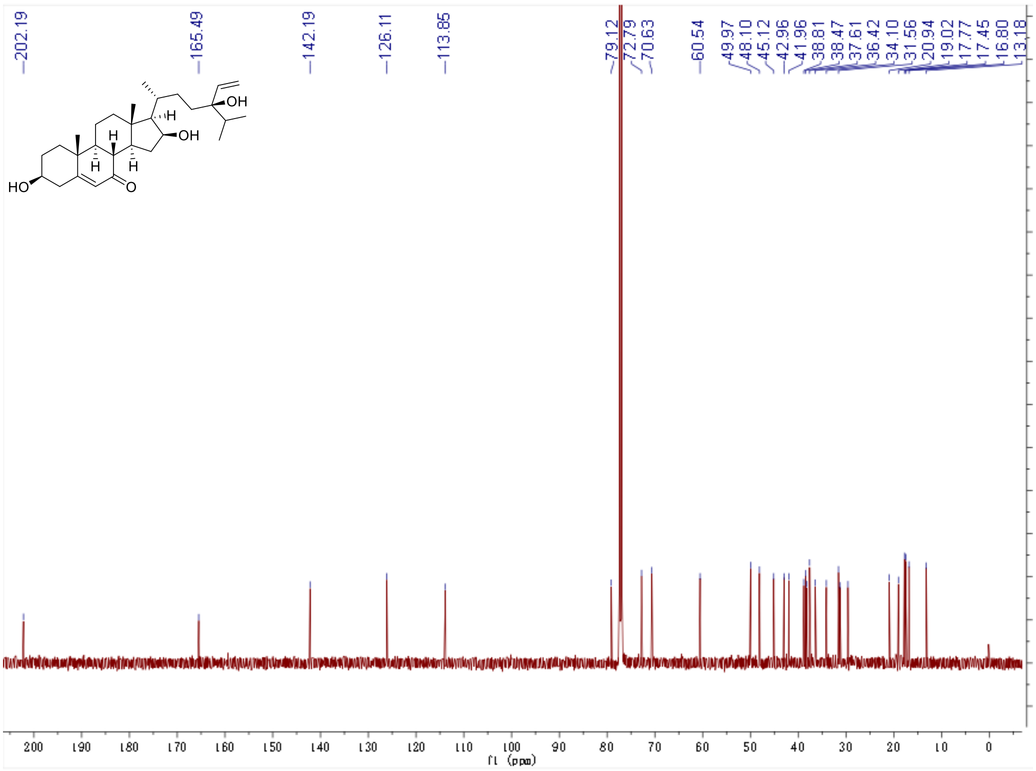

Figure S42. ^{13}C NMR spectrum of compound **5** in $CDCl_3$ (150 MHz)

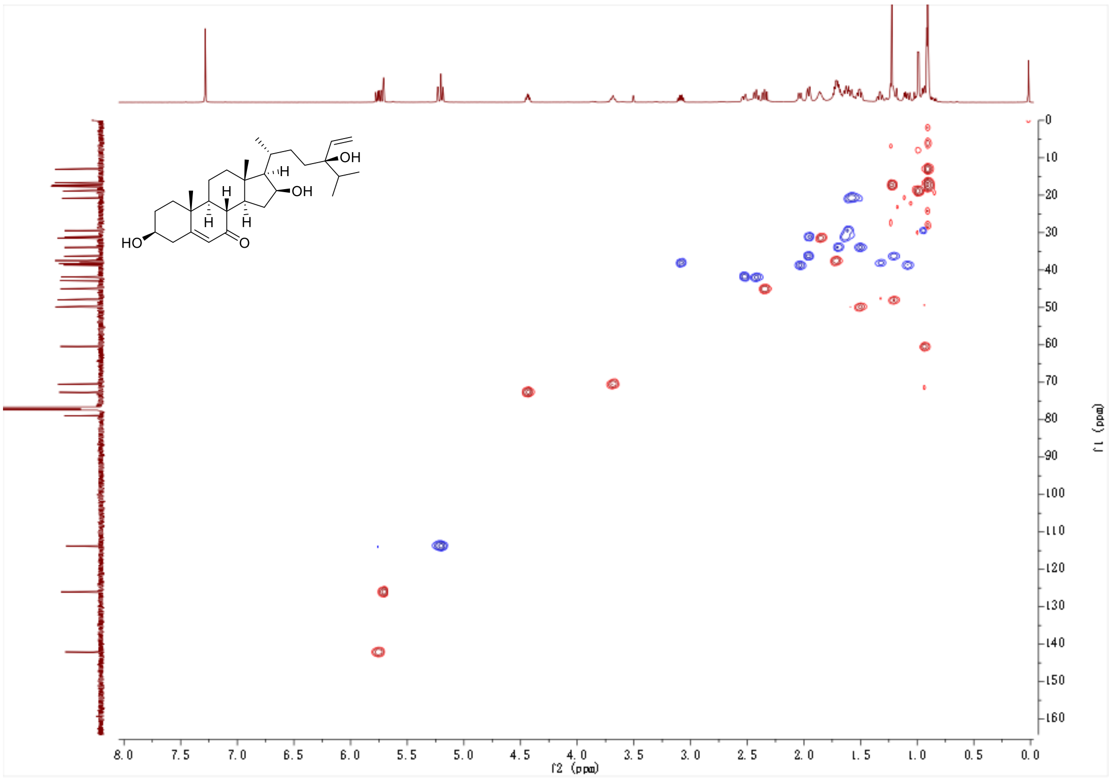

Figure S43. HSQC spectrum of compound **5** in $CDCl_3$

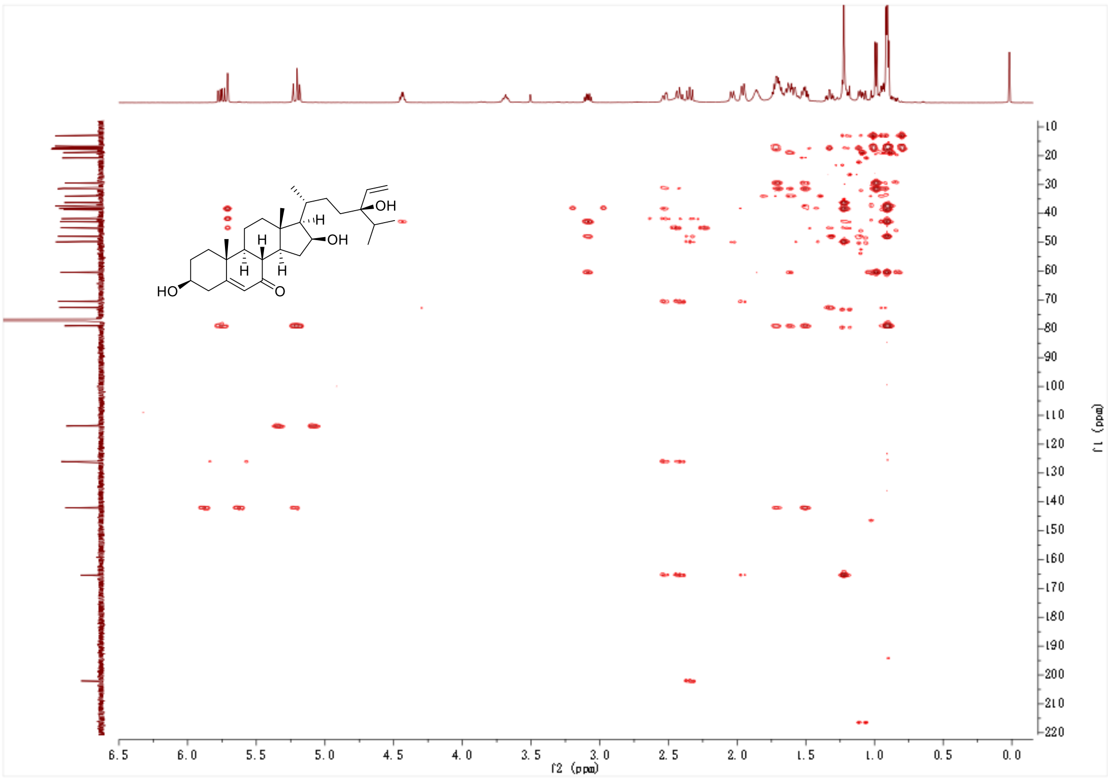

Figure S44. HMBC spectrum of compound **5** in $CDCl_3$

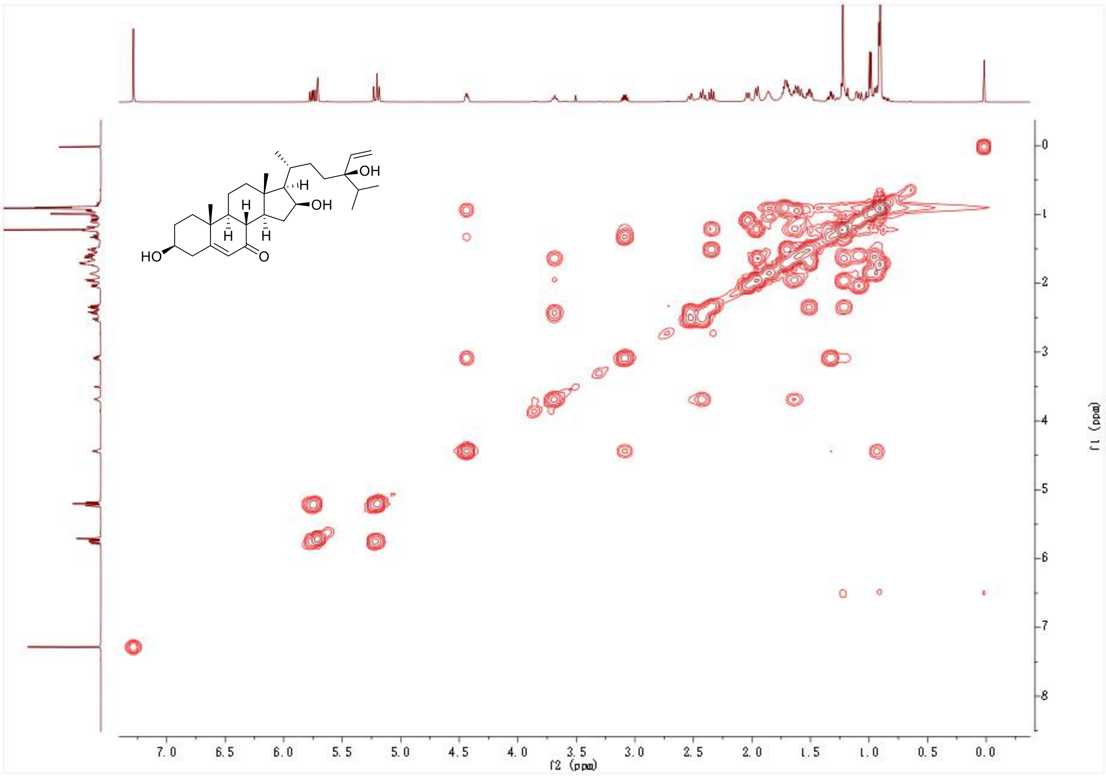

Figure S45. ^{1}H-^{1}H COSY spectrum of compound **5** in $CDCl_3$

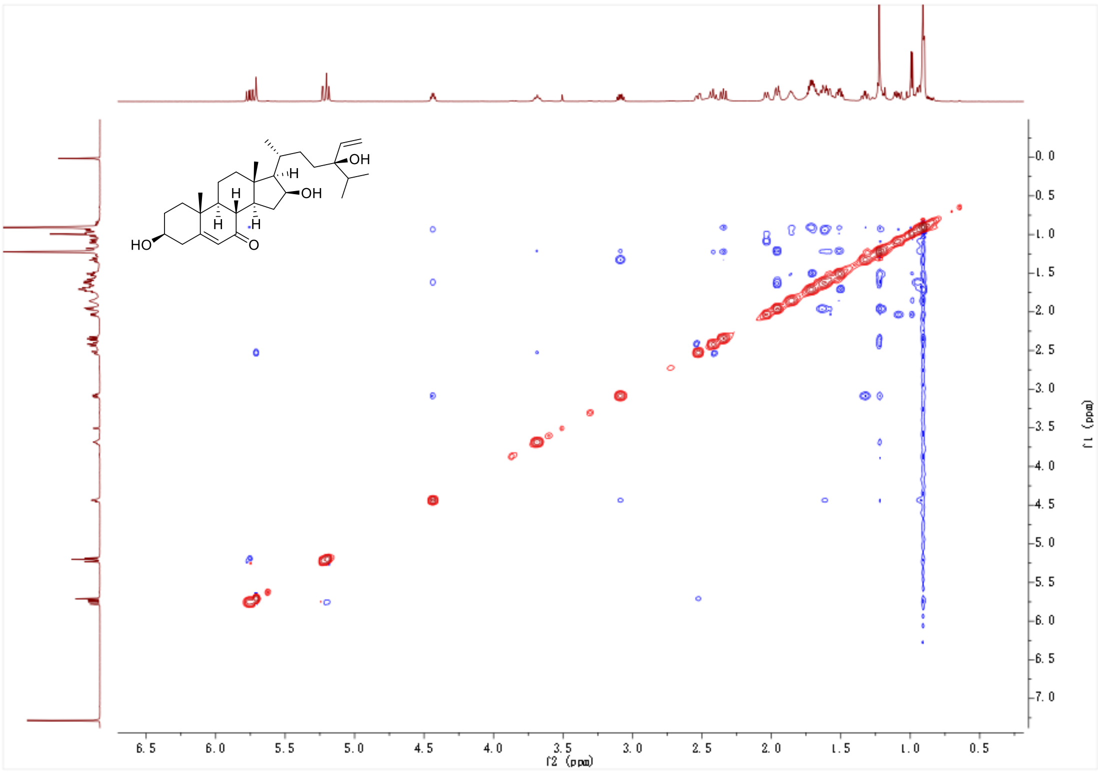

Figure S46. NOESY spectrum of compound **5** in $CDCl_3$

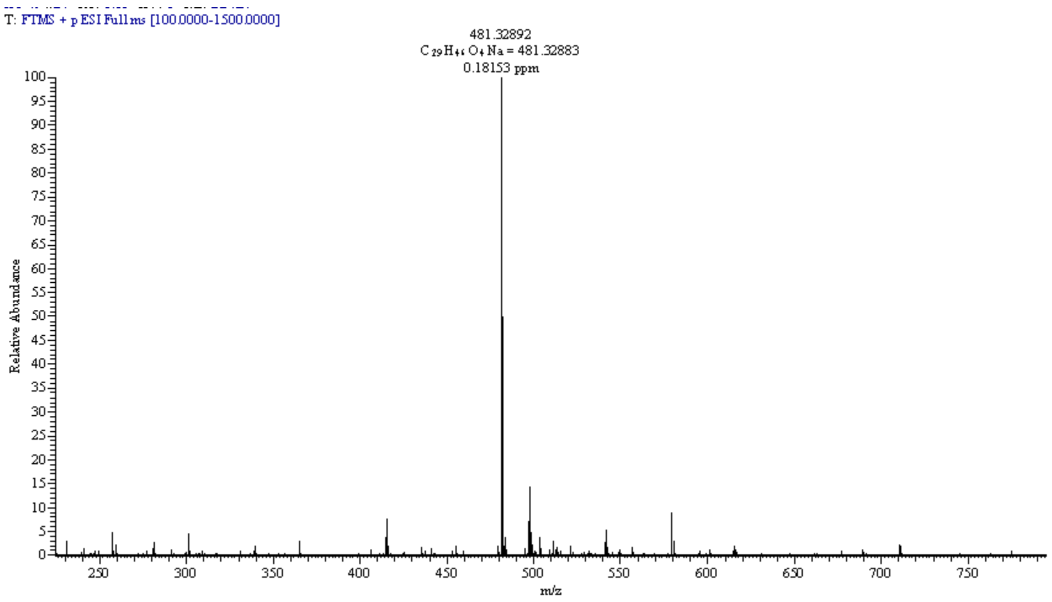

Figure S47. HRESIMS spectrum of compound **5**

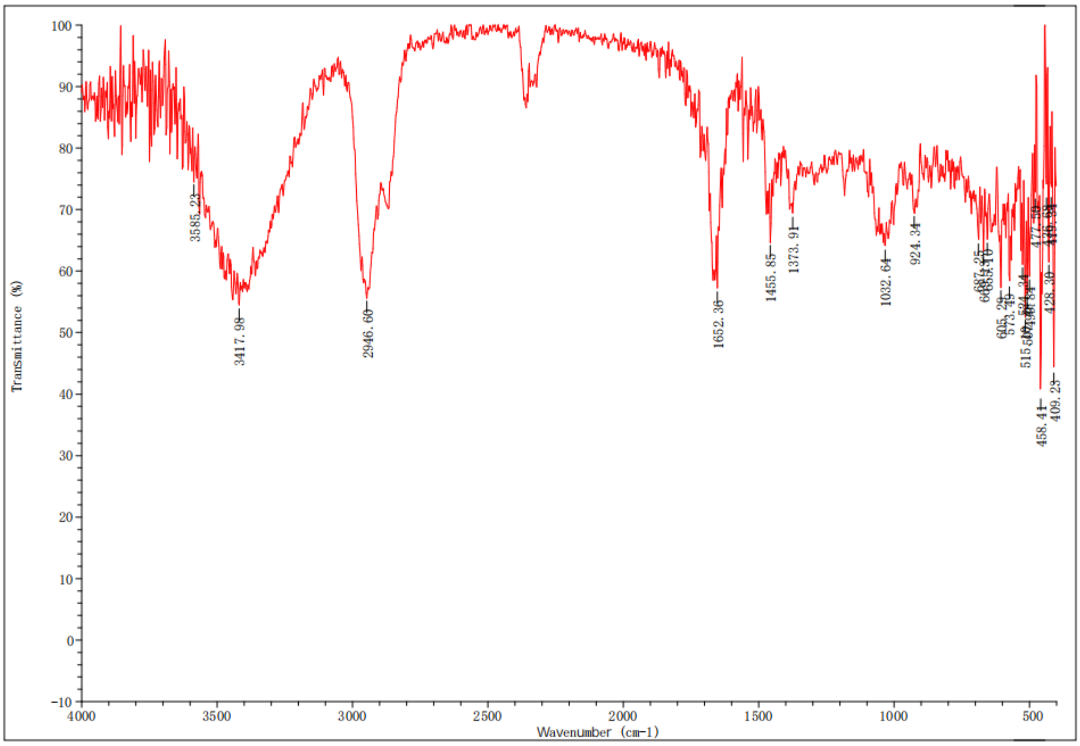

Figure S48. IR spectrum of compound **5**

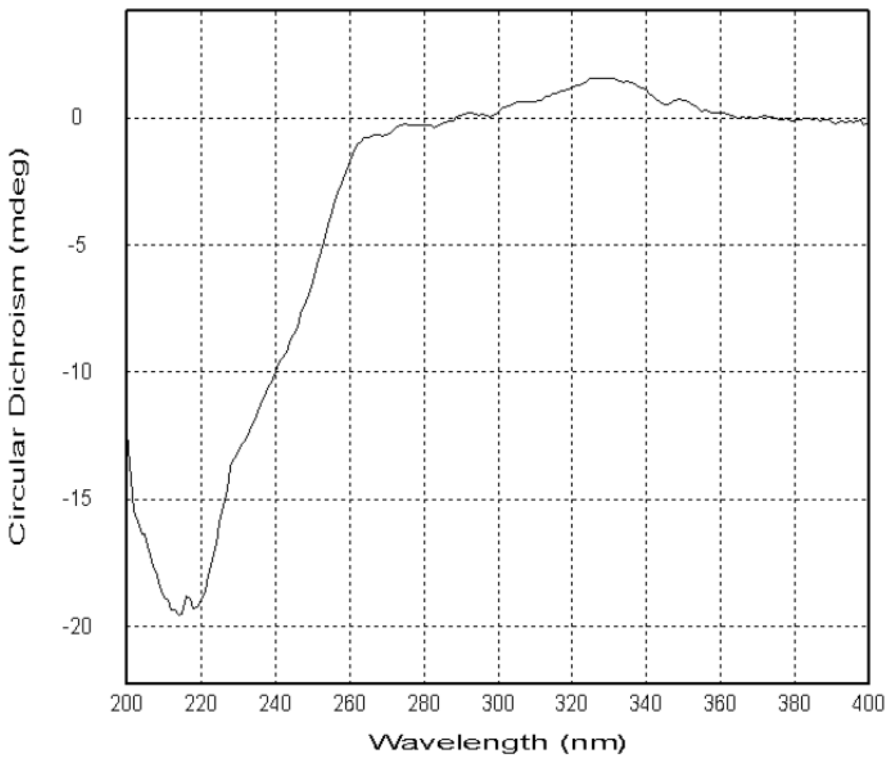

Figure S49. UV spectrum of compound **5**

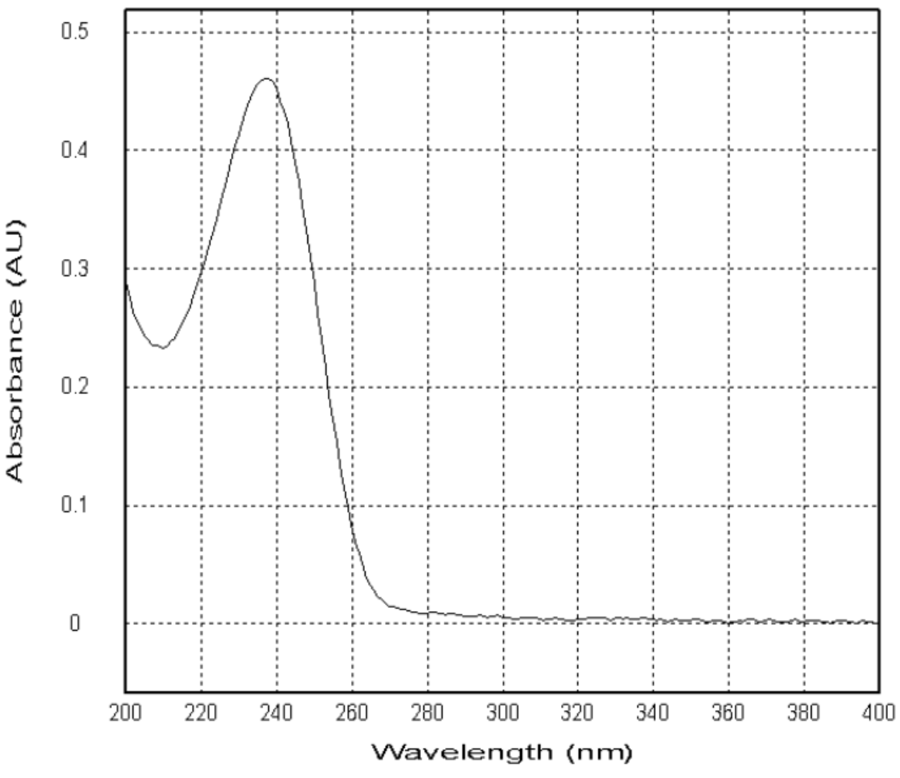

Figure S50. CD spectrum of compound **5**

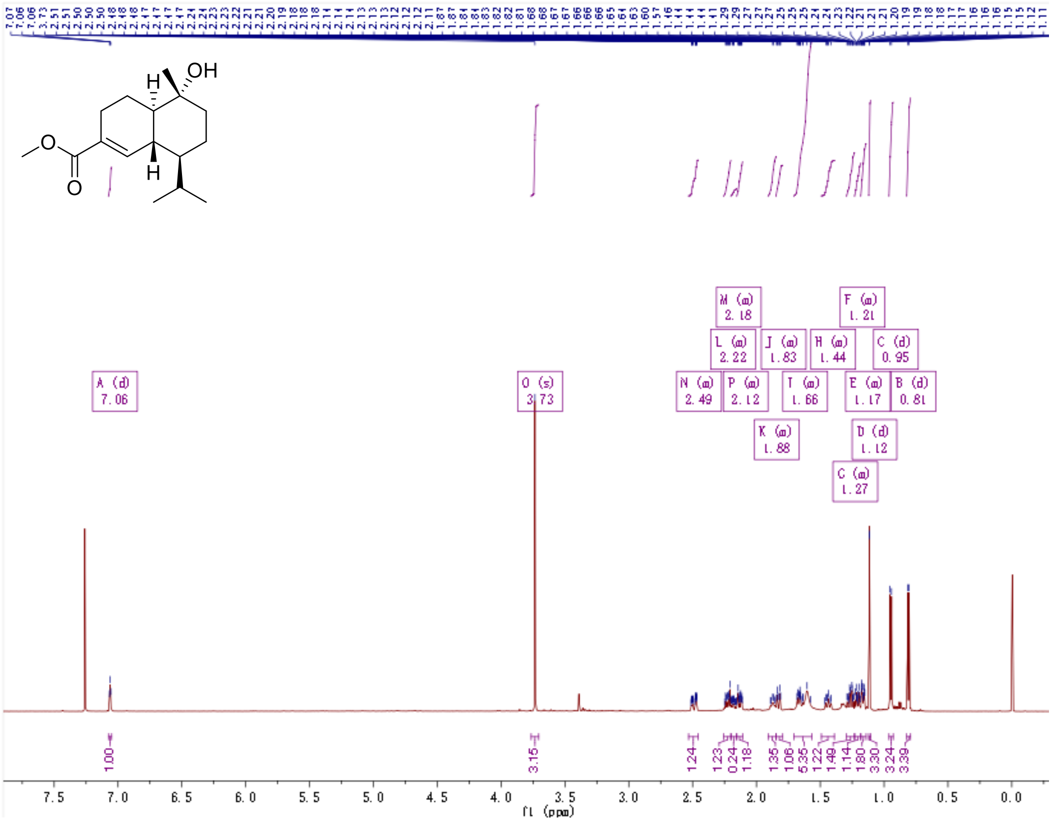

Figure S51. ^{1}H NMR spectrum of compound **6** in $CDCl_3$ (600 MHz)

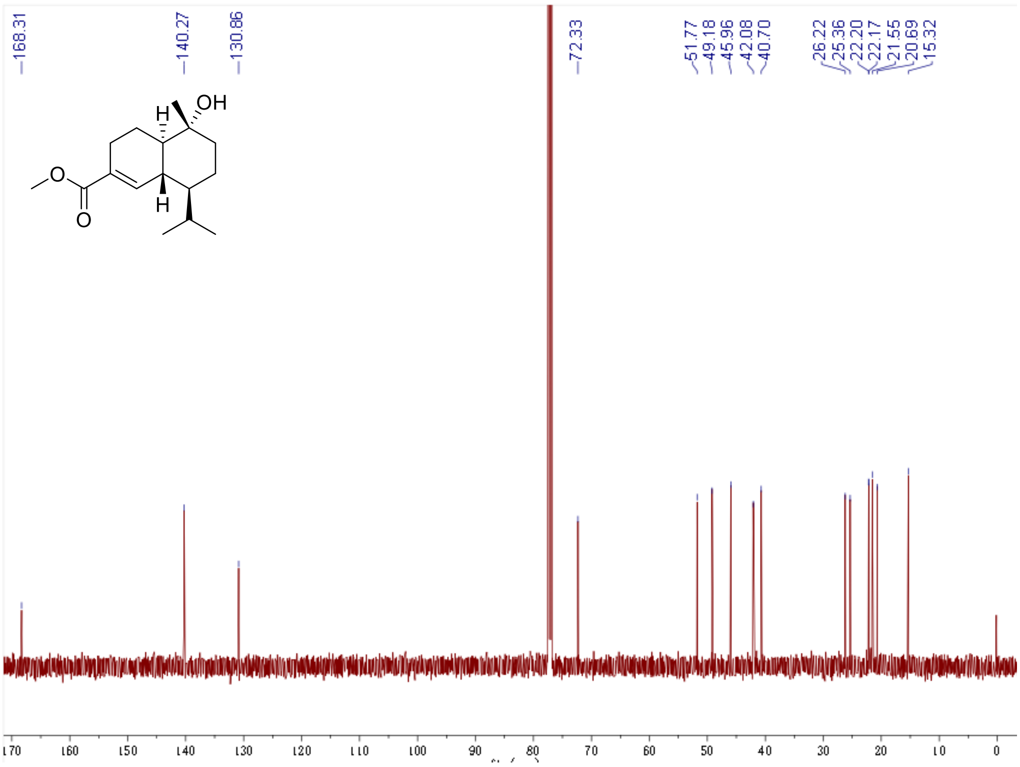

Figure S52. ^{13}C NMR spectrum of compound **6** in $CDCl_3$ (150 MHz)

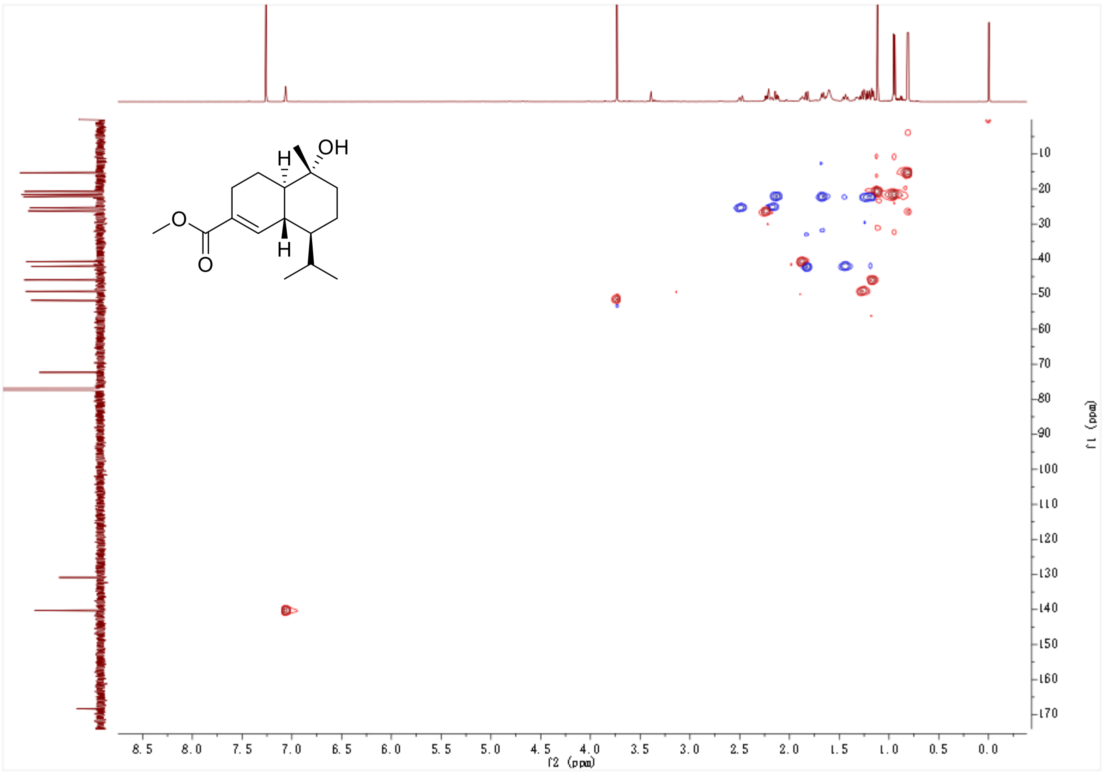

Figure S53. HSQC spectrum of compound **6** in $CDCl_3$

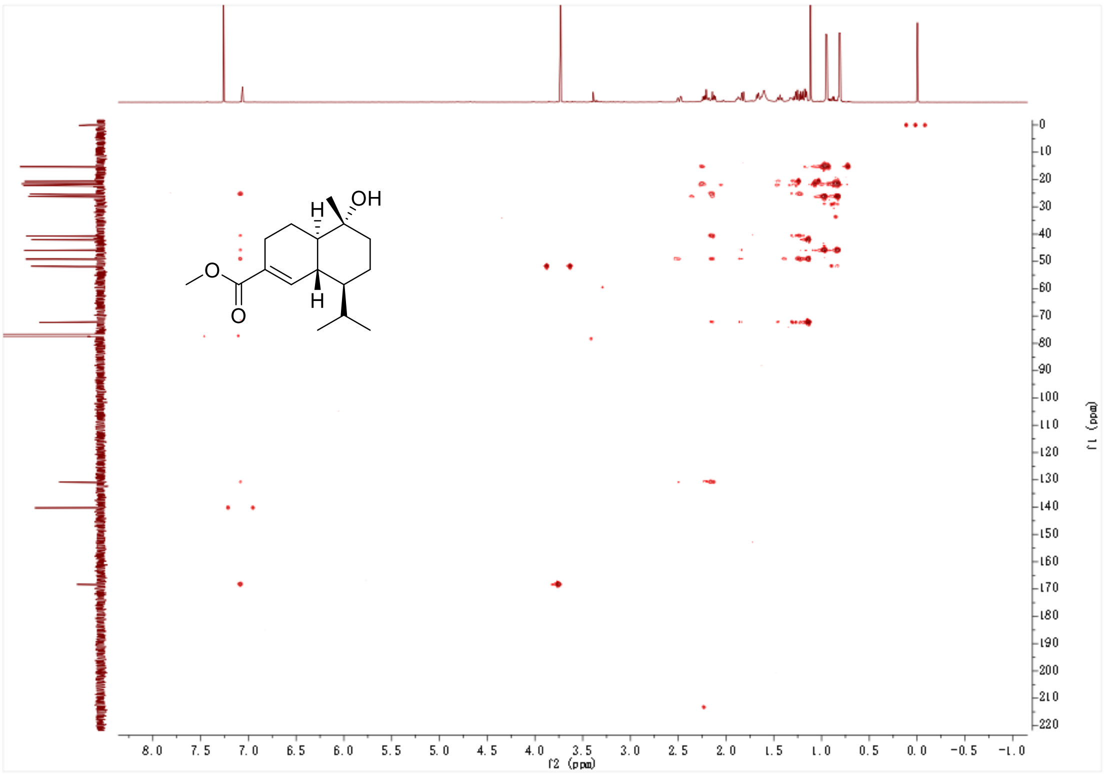

Figure S54. HMBC spectrum of compound **6** in $CDCl_3$

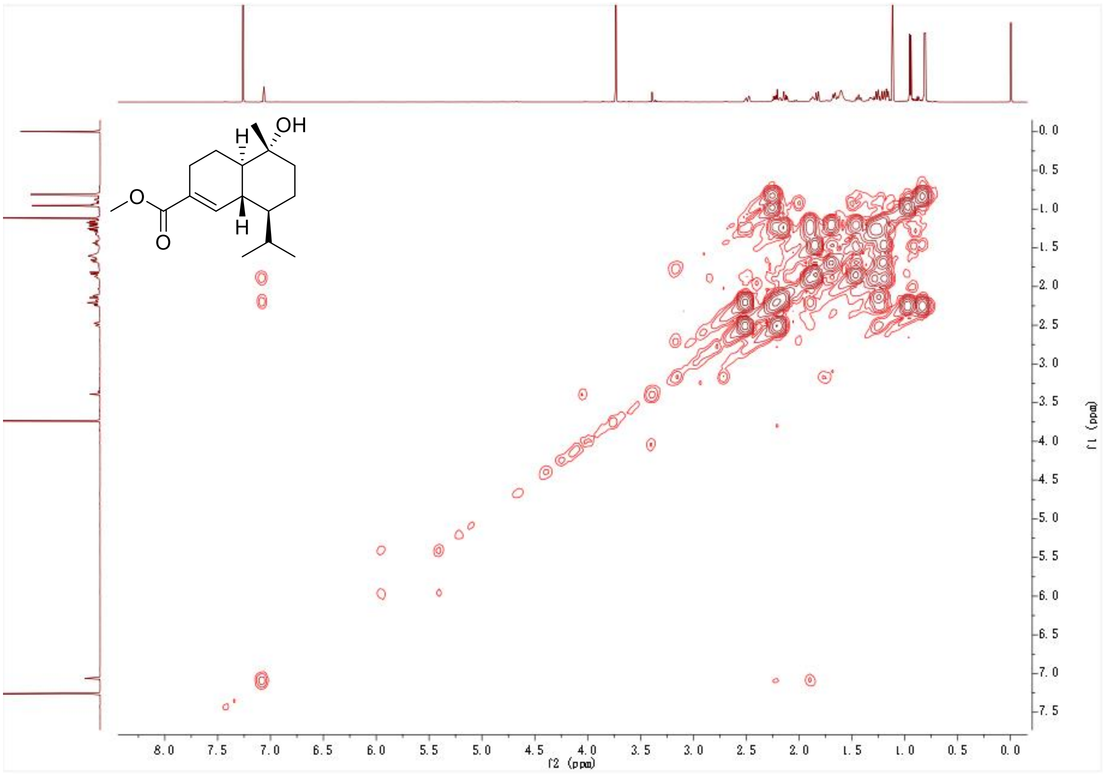

Figure S55. ^{1}H-^{1}H COSY spectrum of compound **6** in $CDCl_3$

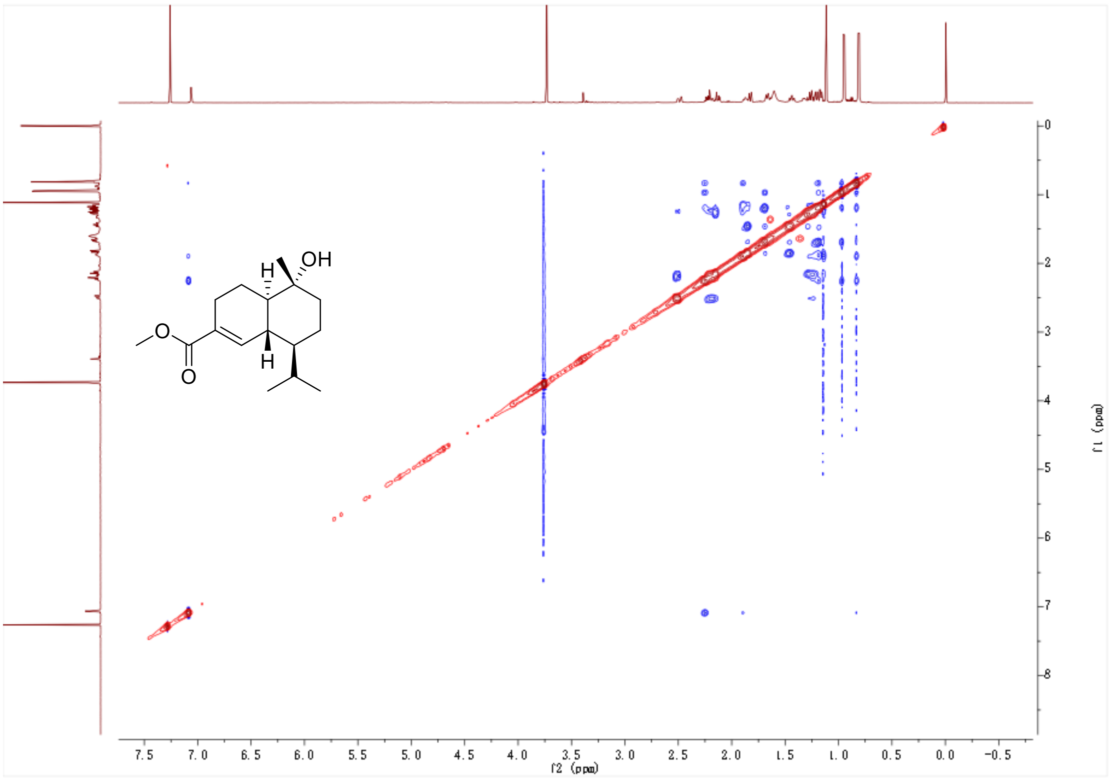

Figure S56. NOESY spectrum of compound **6** in $CDCl_3$

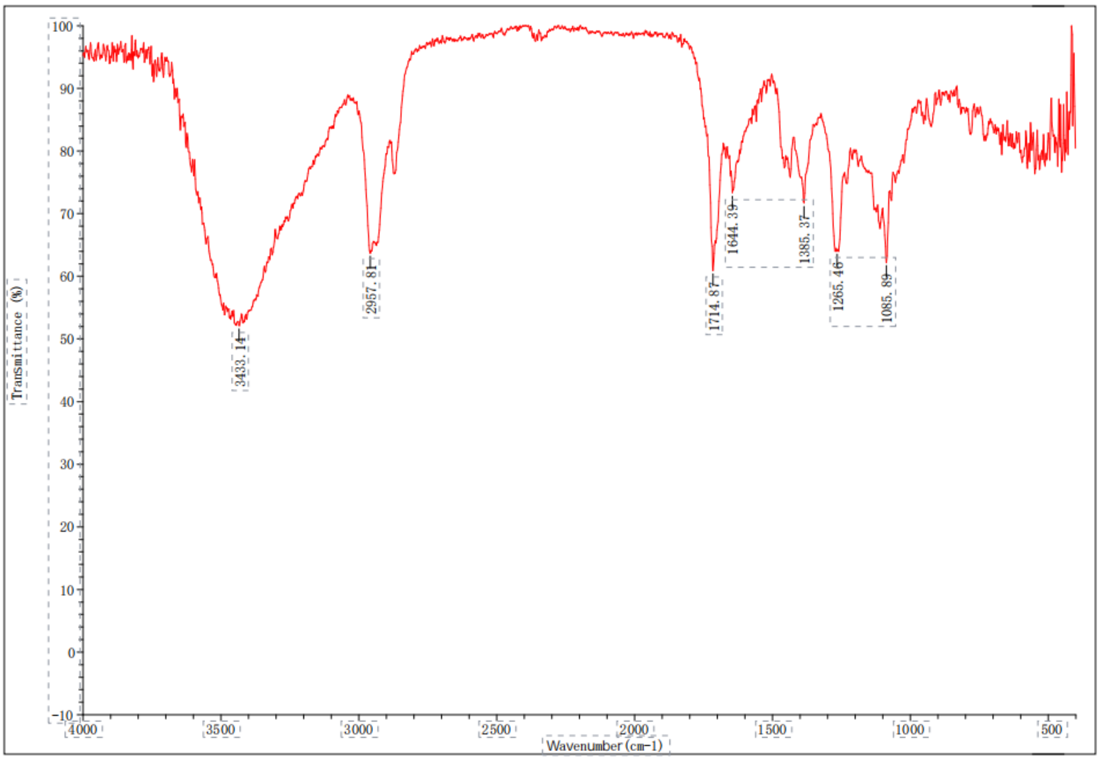

Figure S57. IR spectrum of compound **6**

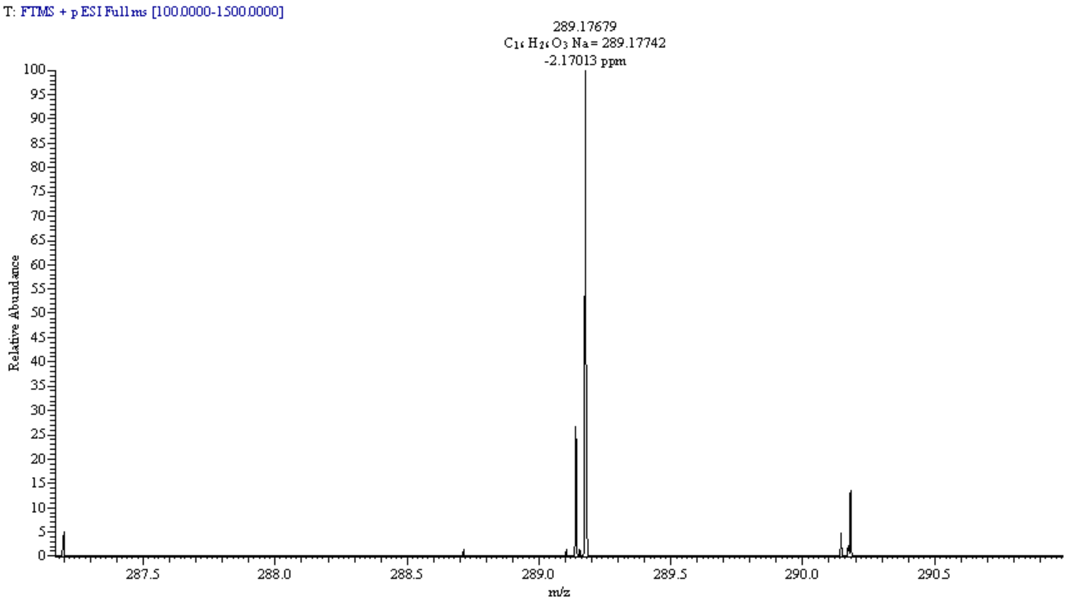

Figure S58. HRESIMS spectrum of compound **6**

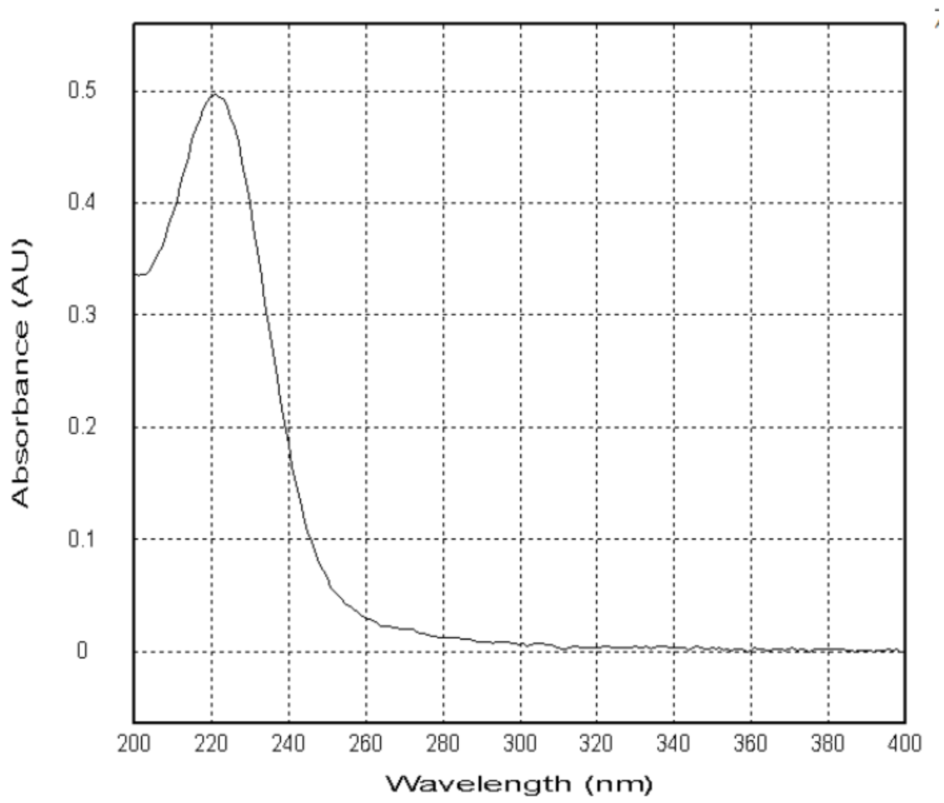

Figure S59. UV spectrum of compound **6**

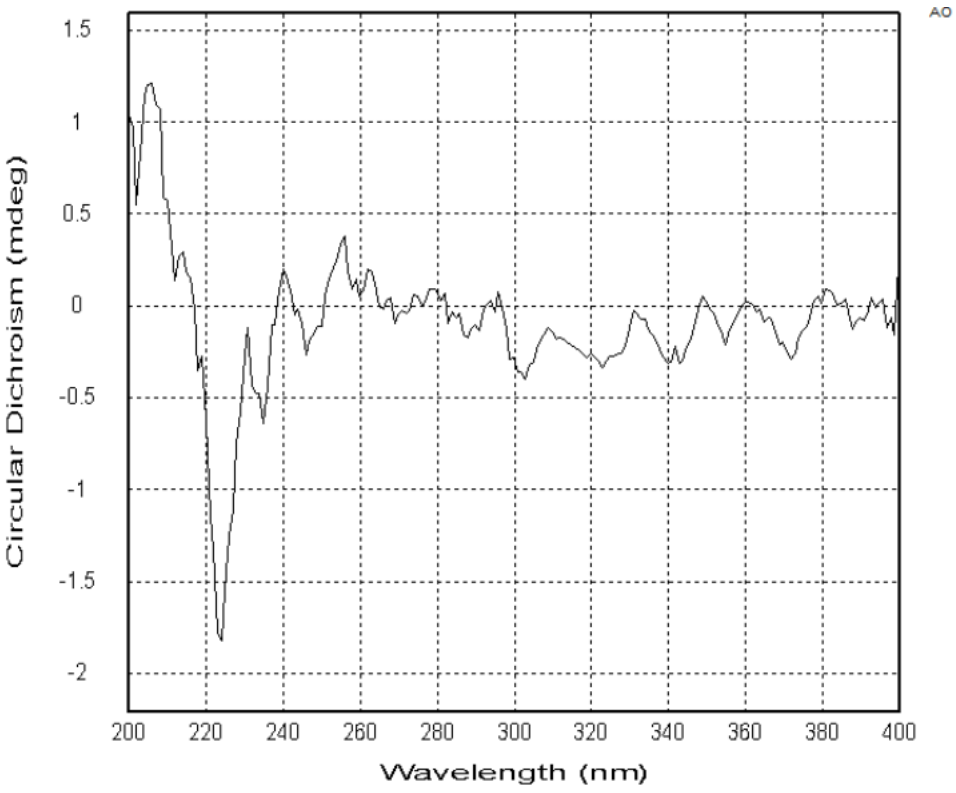

Figure S60. CD spectrum of compound **6**

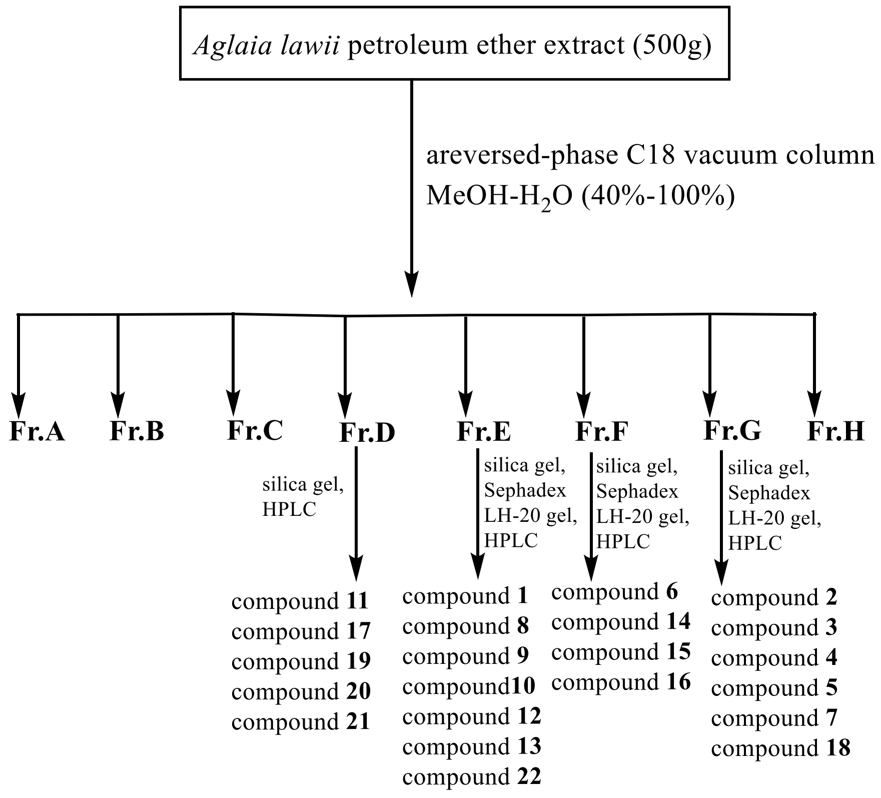

Figure S61. Compounds separation flowchart
